# Supplementary material for: Prospective observational study of the challenges in diagnosing common neonatal conditions in Nigeria and Kenya
Source: BMJ Open. 2022 Dec 6;12(12):e064575. doi: 10.1136/bmjopen-2022-064575 (PMC9730357; doi:10.1136/bmjopen-2022-064575)
Supplement: Supplementary data [file bmjopen-2022-064575supp001.pdf]

***Appendix 1**: Neonatal Nutrition Network Members*

Isa Abdulkadir (Ahmadu Bello University, Nigeria); Ismaela Abubakar (LSTM, UK); Abimbola E Akindolire (College of Medicine, University of Ibadan, Nigeria); Olusegun Akinyinka (College of Medicine, University of Ibadan, Nigeria); Stephen J Allen (LSTM, UK); Pauline EA Andang'o (Maseno University, Kenya); Graham Devereux (LSTM, UK); Chinyere Ezeaka (Lagos University Teaching Hospital, Nigeria); Beatrice N Ezenwa (Lagos University Teaching Hospital, Nigeria); Iretiola B Fajolu (Lagos University Teaching Hospital, Nigeria); Zainab O Imam (Lagos State University Teaching Hospital, Nigeria); Kevin Mortimer (LSTM, UK); Martha K Mwangome (KEMRI Wellcome Trust Research Programme, Kenya); Helen M Nabwera (LSTM, UK); Grace M Nalwa (Jaramogi Oginga Odinga Teaching and Referral Hospital, Kenya & Maseno University, Kenya); Walter Otieno (Jaramogi Oginga Odinga Teaching and Referral Hospital, Kenya & Maseno University, Kenya); Alison W Talbert (KEMRI Wellcome Trust Research Programme, Kenya); Nicholas D Embleton (Newcastle University, UK); Olukemi O Tongo (College of Medicine, University of Ibadan, Nigeria); Dominic D Umoru (Maitama District Hospital, Nigeria); Janneke van de Wijgert (University of Liverpool, UK); Melissa Gladstone (University of Liverpool, UK).

Appendix 2: Neonatal unit details

Location	Rural or urban	Secondary of tertiary level	No. of neonatal beds	Staffing
The Neonatal Unit, Lagos University Teaching Hospital (luth), Lagos, Nigeria	Urban	Tertiary	80 beds	- 4 consultant neonatologists<br>- 10 resident doctors<br>- 26 nurses<br>- 8 support staff
The Neonatal Unit of Ahmadu, Bello University Teaching Hospital Shika, Zaria, Kaduna, Nigeria	Urban	Tertiary	24 beds	- 3 neonatologists<br>- 5 senior registrars<br>- 4 registrars<br>- 18 nurses<br>- Support staff
Neonatal Unit, Maitama District Hospital, Abuja, Nigeria	Urban	Secondary	8 beds	- 3 neonatologists<br>- 2 medical Officers<br>- 2-3 nurses<br>- Support staff
Massey Street Children's Hospital, Lagos Island, Nigeria	Urban	Tertiary	56 beds	- 2 neonatologists<br>- 1 senior registrar<br>- 4 registrars<br>- 31 nursing staff
University College Hospital, Ibadan, Nigeria	Urban	Tertiary	38 beds	- 3 neonatologists<br>- 4 senior registrars<br>- 4 registrars<br>- House officers<br>- 50 nurses
Kilifi County Hospital, Kilifi Town, Kenya	Urban	Secondary	18 beds	- 2 paediatric consultants,<br>- 6 clinical officers<br>- 3-4 neonatal nurses
Jaramogi Oginga Odinga Teaching and Referral Hospital (JOOTRH), Kisumu City, Kenya	Urban	Tertiary	21 beds	- 2 paediatric consultants,<br>- 2 medical officers<br>- 1-2 medical officers<br>- 1-2 neonatal nurses

Appendix 3a: Ethical approval details from collaborating institutions

Institution	Protocol number
**Liverpool School of Tropical Medicine Research Ethics Committee (REC)**	18-0210
**The Lagos University Teaching Hospital Health Research Ethics Committee**	AMD/DCST/HREC/APP/2514
**The research and ethics committees at The Jaramogi Oginga Odinga Teaching and Referral Hospital**	ERC.IB/VOL.1/510
**University College Hospital Ibadan**	UI/EC/18/0446
**Massey Street Children's Hospital**	LSHSC/2222/VOL.VI^{B}/185
**Ahmadu Bello University Teaching Hospital**	ABUTH/HZ/HREC/D37/2018
**Maitama District Hospital**	FHREC/2018/01/108/19-09-18
**The Kenya Medical Research Institute-Scientific and Ethics Review Unit**	KEMRI/SERU/CGMR-C/120/3740

Appendix 3b: Confirmation of data access request from KEMRI- Wellcome Trust

1. REQUESTOR DETAILS

**Name**	Aimee Staunton				
**Contact details: address, phone and email**	97 Aigburth Road, Liverpool L17 4JU<br>Liverpool School of Tropical Medicine	**Phone**	07530351521	**Email**	264266@lstmed.ac.uk

2. STUDY DETAILS

**Title of Research Project**	Describing the diagnostic pathways for common neonatal conditions across seven neonatal units in sub-Saharan Africa	**Start & end dates**	June 2020- September 2020
**Ethical approval sought / planned (with dates)**	Ethics application covered by existing approved proposal:<br><br>Title of proposal: Improving the survival, growth and development of low birth weight newborns through better nutrition<br>LSTM Research Ethics Committee, reference number: 18-0210<br>PI: Prof Stephen Allen<br>Date of approval: 4th July 2018<br>End date for data collection: 31st August 2019		

3. INVESTIGATORS

Names of all investigators who will have access to data (begin with Principal Investigator)* include names of KWTRP collaborators where applicable.

Name	Role	Institution
Aimee Staunton	Principal Investigator (MSc student)	LSTM
Helen Nabwera	Project Supervisor	LSTM
Stephen Allen	Project Supervisor	LSTM

4. BRIEF DESCRIPTION TYPE OF DATA REQUIRED

The dataset collected by NeoNuNet to record diagnostic pathways, comprising all admissions to the seven network neonatal units over a six-month period between August 2018 and May 2019. This includes 2280 neonates in total.

Title: Improving the survival, growth and development of low birth weight newborns through better nutrition

PI: Dr Martha Mwangome

KEMRI/SERU/CGMT-C/120/3740

5. OBJECTIVES AND STUDY DETAILS INCLUDING TIME FRAME

1. To describe the population of neonates and the causes of admission to secondary and tertiary level neonatal units in sub-Saharan Africa using secondary data collected from seven neonatal units in Nigeria and Kenya by September 2020.
2. To describe how common neonatal conditions (asphyxia, respiratory disorders, abdominal conditions/ necrotizing enterocolitis and neonatal sepsis) are diagnosed in the seven neonatal units in Nigeria and Kenya by September 2020.
3. To identify any gaps or inaccuracy in the diagnostic pathways for common neonatal conditions in the seven neonatal units and explore the association with neonatal outcomes by September 2020.

6. BRIEF DESCRIPTION OF ANALYSIS PLANNED WITHIN KIDMS DATA ***specify if study specific data not KIDMS data**

Objective 1: To fulfil this objective, data will be analysed using SPSS to describe the population of neonates. The relative percentages of the different diagnoses will be calculated. Descriptive statistics for variables such as gestation, sex and birthweight will be generated and basic hypothesis testing will be carried out. This will help to describe the biodemographic characteristics of the population being studied.

Objective 2: To fulfil this objective, the diagnostic pathways that lead to the final diagnosis (birth asphyxia, respiratory disorders, abdominal conditions/ necrotizing enterocolitis (NEC) or neonatal sepsis) will be analysed. This will be

Conditions and limitations for data sharing

You understand and acknowledge that custodianship of the Data is held jointly by the Programme and the Ministry of Health in Kilifi, and will be retained by the Programme and the Ministry of Health in Kilifi in case of any data transfer to a Recipient.
You understand and acknowledge that the Data is experimental in nature, and that access to the Data is provided without any representations or warranties of any kind in relation to the Database or the Data.
You will only access the Database and use the Data for the Agreed Purpose and shall not use the Data in such a way that damage or distress is or is reasonably likely to be caused to any Data Subjects.
The Data relates directly to individual Data Subjects and is strictly confidential. Data from subjects will be shared in a fully anonymised form without access to personal identifiers, including names and/or specialised roles. Geographic identifiers for subjects will routinely be aggregated to the level of the sub location or similarly de-identified. Consideration of requests for data with greater granularity will be informed by the local importance of the research question and the ability to protect the confidentiality of individuals and communities.
You shall only disclose the Data to your Data Users. You shall take all reasonable measures to ensure that your Data Users shall not make copies of the whole or any part of the Data without your consent, and shall keep a written record of any such copies sufficient to permit you to fulfil your obligations under clauses 3 and 7 of this Agreement. You shall not transfer or disclose any part of the Data to any other person or body.
You understand and acknowledge that the Data is protected by copyright and other intellectual property rights.
You agree at all times to keep strictly confidential, and ensure that the Data Users keep confidential the information and Data pertaining to Data Subjects. In particular, You undertake not to use, or attempt to use, or permit anyone other than the Data Users to use the Data on its own or in conjunction with other data, to seek to discover the identity of any Data Subjects, to compromise or otherwise infringe the confidentiality of information on Data Subjects and their right to privacy.
You accept that the Data is protected by and subject to national and international laws, and that You are responsible for ensuring compliance with any applicable laws.
You agree that you will submit a summary report and any other information reasonably requested to the KEMRI Wellcome Trust Research Programme for the purposes of monitoring data use on the understanding that such information, data, results, and conclusions contained within will be treated as confidential information belonging to the Recipient.
This agreement is not transferable and You may not purport to assign it (in whole or in part) without the written consent of the KEMRI Wellcome Trust Research Programme.
If You commit a material breach of this Agreement or for any persistent breach of this Agreement, the KEMRI Wellcome Trust may terminate this Agreement immediately by notice in writing, without prejudice to its accrued rights and remedies.
You accept that it may be necessary for the KEMRI Wellcome Trust Research Programme to alter the terms of this Agreement from time to time in order to address new concerns. In this event, the KEMRI Wellcome Trust Research Programme will contact You to inform You of any changes and You agree that Your continued use of the Data shall be dependent on the parties entering into a variation of the Agreement.
If requested to do so by the Data Governance Committee, the KEMRI Wellcome Trust Research Programme may terminate this Agreement at any time by giving one month's notice in writing to You.
Your duty to protect the confidentiality of the Data received under this agreement shall survive termination of this Agreement and shall continue in full force and effect indefinitely.
Upon termination of this Agreement you will permanently delete all copies of the Data from your computer systems and storage devices and will ensure that all Your Data Users shall also permanently delete such copies. You may retain aggregate information on the proviso that such aggregate information does not allow any individuals or groups of individuals who are the subject of the Data to be identified with reasonable effort.
You may publish Your results arising from the use of the Data for the Agreed Purpose providing the Data itself is not disclosed. Aggregate or generic information generated from the Data may be published on the provisos that: i) such aggregate or generic information does not allow Data Subjects or groups of Data Subjects to be identified with reasonable effort; ii) no damage or distress is or is reasonably likely to be caused to any Data Subjects or groups of Data Subjects; iii) the Data will not be used in any way that could reasonably be expected to lead to ethnic stigmatisation; and iv) no attempt will be made to identify the Data Subjects.
You agree to acknowledge the KEMRI Wellcome Trust Research Programme or appropriate Programme researchers (via authorship or an acknowledgment statement) in any work based in whole or part on the Data in the following way :*"This paper/publication has used data and/or biological samples provided by the KEMRI Wellcome Trust Research programme in accordance with the consent provided by participants and approved by the KEMRI Ethics Review Committee".*
You agree that if your application is approved, information about the proposed research use can be posted on the KEMRI Wellcome Trust Research Programme's public website. The information may include Your name and institution, the title of the project, and a non-technical summary of the research question.
You recognise that nothing in this Agreement shall operate to transfer to You any intellectual property rights relating to the Data. You have the right to develop intellectual property based on comparisons with Your own data.

Definitions in the agreement above:
"Recipient" means the principal researcher named above; "Recipient Institution" means the organisation named above at which the Recipient is employed, affiliated or enrolled; "Data Users" means those officers, employees and students of the Recipient Institution, who work directly with the Recipient and have a need to use the Data for the performance of their work with respect to the Agreed Purpose, and have agreed to comply with this Agreement, as named in the application; "Database" the KIDMS database containing clinical and demographic and health surveillance data on Data Subjects; "Data Subjects" the individuals who have contributed their data to the Database; "Data" the data within the Database; "Agreed Purpose" means the medical research purpose(s) approved by the Data Governance Committee in writing; "Publications" means, without limitation, articles published in print journals, electronic journals, reviews, books, posters and other written and verbal presentations of research.

Statement of agreement: I agree to the above conditions and limitations for sharing data as requested in this application:

Name:____AIMEE STAUNTON____________________
Date:____04/06/20____________________

Signature:____________________

Signed on behalf of KEMRI-Wellcome Trust Research Programme:

....

Title:...Professor Philip Bejon..

Position:...Programme Executive Director...

Signature: ....

Date:......29/Jun/2020...

Appendix 4: Data from heatmaps in Figures 1-4 showing diagnostic criteria use in common neonatal conditions.

4a. Suspected sepsis

**NEONATAL SEPSIS**																
	CRITERIA NUMBER															
NNU	1.	2.	3.	4.	5.	6.	7.	8.	9.	10.	11.	12.	13	14.	15.	16.
1	1.00	1.00	1.00	1.00	1.00	1.00	0.00	1.00	1.00	1.00	0.24	0.00	0.00	0.99	0.46	0.01
2	0.99	0.98	0.89	0.98	1.00	0.98	0.79	0.35	0.38	0.38	0.02	0.01	0.01	0.02	0.01	0.01
3	1.00	1.00	1.00	1.00	1.00	1.00	0.12	0.97	0.99	0.99	0.01	0.00	0.00	0.15	0.03	0.01
4	1.00	1.00	0.88	0.98	0.99	0.93	0.59	0.96	0.92	0.92	0.89	0.84	0.13	0.72	0.27	0.26
5	1.00	1.00	1.00	1.00	1.00	0.96	0.96	1.00	0.96	0.96	0.08	0.04	0.04	0.02	0.00	0.00
6	1.00	1.00	1.00	1.00	1.00	0.70	0.98	0.59	1.00	1.00	1.00	0.89	0.98	1.00	0.72	0.54
7	1.00	1.00	0.89	0.99	1.00	0.99	0.73	0.96	0.76	0.74	0.70	0.36	0.35	0.75	0.37	0.19

4b. Respiratory conditions

**RESPIRATORY CONDITIONS**															
	CRITERIA NUMBER														
NNU	1	2.	3.	4.	5.	6.	7.	8.	9.	10.	11.	12.	13.	14.	15.
1	1.00	1.00	1.00	1.00	1.00	0.99	1.00	1.00	1.00	1.00	1.00	1.00	0.51	0.50	0.97
2	0.93	0.88	0.95	0.91	0.93	0.98	0.99	0.99	0.99	0.99	0.99	0.99	0.10	0.04	0.78
3	1.00	1.00	1.00	1.00	1.00	1.00	1.00	1.00	1.00	1.00	1.00	1.00	0.47	0.37	1.00
4	1.00	0.98	0.97	0.98	0.99	0.96	1.00	1.00	0.99	0.96	1.00	0.99	0.26	0.26	0.85
5	0.56	1.00	0.89	1.00	1.00	1.00	1.00	1.00	1.00	1.00	1.00	1.00	0.06	0.06	1.00
6	1.00	0.98	0.99	0.99	0.99	0.99	0.99	0.99	0.99	0.99	0.99	0.99	0.14	0.14	0.99
7	1.00	1.00	1.00	1.00	1.00	1.00	1.00	1.00	1.00	0.96	0.87	0.96	0.39	0.39	0.96

*4*c. Birth asphyxia

BIRTH ASPHYXIA						
	CRITERIA NUMBER					
NNU	1.	2.	3.	4.	5.	6.
1	1.00	0.02	1.00	0.63	1.00	0.00
2	0.75	0.75	0.84	0.07	0.72	0.04
3	1.00	0.95	1.00	0.10	1.00	0.00
4	0.99	0.88	0.96	0.77	0.98	0.23
5	1.00	0.85	0.88	0.78	0.98	0.39
6	0.91	0.73	0.93	0.95	1.00	0.84
7	0.91	0.76	0.73	0.49	0.95	0.51

4d. Abdominal conditions

ABDOMINAL CONDITIONS																						
	CRITERIA NUMBER																					
NNU	1.	2.	3.	4.	5.	6.	7.	8.	9.	10.	11.	12.	13.	14.	15.	16.	17.	18.	19.	20.	21.	22
1	1.00	1.00	1.00	1.00	1.00	1.00	1.00	1.00	1.00	1.00	1.00	1.00	1.00	1.00	1.00	0.33	0.33	0.33	0.33	0.33	0.33	0.33
2	1.00	1.00	1.00	1.00	1.00	1.00	1.00	1.00	1.00	1.00	1.00	0.75	0.75	0.75	0.75	0.00	0.75	0.75	0.75	0.75	0.75	0.75
3	1.00	0.67	0.83	1.00	1.00	1.00	1.00	1.00	1.00	1.00	1.00	1.00	1.00	1.00	1.00	0.67	0.67	0.67	0.50	0.67	0.67	0.67
4	1.00	0.94	0.78	0.88	0.97	0.94	1.00	0.81	0.94	1.00	1.00	0.97	0.94	0.94	0.97	0.97	0.75	0.72	0.69	0.75	0.78	0.72
5	0.67	0.67	0.67	0.67	0.67	0.67	0.67	0.67	0.67	0.67	0.67	0.67	0.67	0.67	0.67	0.67	0.00	0.00	0.00	0.00	0.00	0.00
6	1.00	1.00	1.00	1.00	1.00	1.00	1.00	1.00	1.00	1.00	1.00	1.00	0.93	0.93	0.93	0.93	0.93	1.00	1.00	1.00	0.93	0.93
7	1.00	1.00	1.00	1.00	1.00	1.00	0.89	0.89	0.89	1.00	1.00	0.67	0.56	0.56	0.56	0.78	0.56	0.33	0.44	0.56	0.56	0.44

1. For Appendix 4 data: 1.00 means in 100% of cases the diagnostic criterion was used by clinicians
