# Supplementary material for: Prospective observational study of the challenges in diagnosing common neonatal conditions in Nigeria and Kenya
Source: BMJ Open. 2022 Dec 6;12(12):e064575. doi: 10.1136/bmjopen-2022-064575 (PMC9730357; doi:10.1136/bmjopen-2022-064575)
Supplement: Author's manuscript [file bmjopen-2022-064575.draft_revisions.pdf]

BMJ Open

BMJ Open is committed to open peer review. As part of this commitment we make the peer review history of every article we publish publicly available.

When an article is published we post the peer reviewers' comments and the authors' responses online. We also post the versions of the paper that were used during peer review. These are the versions that the peer review comments apply to.

The versions of the paper that follow are the versions that were submitted during the peer review process. They are not the versions of record or the final published versions. They should not be cited or distributed as the published version of this manuscript.

BMJ Open is an open access journal and the full, final, typeset and author-corrected version of record of the manuscript is available on our site with no access controls, subscription charges or pay-per-view fees (http://bmjopen.bmj.com).

If you have any questions on BMJ Open's open peer review process please email info.bmjopen@bmj.com

BMJ Open

Challenges in diagnosing common neonatal conditions in Nigeria and Kenya

Journal:	*BMJ Open*
Manuscript ID	bmjopen-2022-064575
Article Type:	Original research
Date Submitted by the Author:	31-May-2022

Complete List of Authors:	Staunton, Aimee; Liverpool School of Tropical Medicine,<br>Nabwera, Helen; Liverpool School of Tropical Medicine, International Public Health; Alder Hey Children's NHS Foundation Trust<br>Allen, Stephen ; Liverpool School of Tropical Medicine, Child Health; Alder Hey Children's NHS Foundation Trust<br>Tongo, Olukemi; University College Hospital Ibadan<br>Akindolire, Abimbola ; University College Hospital Ibadan<br>Abdulkadir, Isa; Ahmadu Bello University Teaching Hospital<br>Ezeaka, Chinyere; University of Lagos College of Medicine, Paediatrics<br>Ezenwa, Beatrice; University of Lagos College of Medicine, Paediatrics<br>Fajolu, Iretiola; College of Medicine University of Lagos, Department of Paediatrics; Lagos University Teaching Hospital<br>Imam, Zainab; Lagos State University Teaching Hospital<br>Umoru , Dominic ; Maitama District Hospital<br>Otieno, Walter; Maseno University; Jaramogi Oginga Odinga Teaching and Referral Hospital, Kisumu, Kenya<br>Nalwa, Grace; Maseno University, Department of Paediatrics and Child Health, ; Jaramogi Oginga Odinga Teaching and Referral Hospital, Kisumu, Kenya<br>Olwala, Macrine; Jaramogi Oginga Odinga Teaching and Referral Hospital, Kisumu, Kenya<br>Talbert, Alison; KEMRI-Wellcome Trust Research Programme<br>Andang'o, Pauline; Maseno University<br>Mwangome, MK; KEMRI-Wellcome Trust Research Programme,<br>Abubakar, Ismaela; Liverpool School of Tropical Medicine<br>Embleton, Nicholas; Newcastle University; Newcastle Upon Tyne Hospitals NHS Trust

Keywords:	Health policy < HEALTH SERVICES ADMINISTRATION & MANAGEMENT, Public health < INFECTIOUS DISEASES, NEONATOLOGY, Paediatric gastroenterology < PAEDIATRICS

SCHOLARONE™
Manuscripts

I, the Submitting Author has the right to grant and does grant on behalf of all authors of the Work (as defined in the below author licence), an exclusive licence and/or a non-exclusive licence for contributions from authors who are: i) UK Crown employees; ii) where BMJ has agreed a CC-BY licence shall apply, and/or iii) in accordance with the terms applicable for US Federal Government officers or employees acting as part of their official duties; on a worldwide, perpetual, irrevocable, royalty-free basis to BMJ Publishing Group Ltd ("BMJ") its licensees and where the relevant Journal is co-owned by BMJ to the co-owners of the Journal, to publish the Work in this journal and any other BMJ products and to exploit all rights, as set out in our licence.

The Submitting Author accepts and understands that any supply made under these terms is made by BMJ to the Submitting Author unless you are acting as an employee on behalf of your employer or a postgraduate student of an affiliated institution which is paying any applicable article publishing charge ("APC") for Open Access articles. Where the Submitting Author wishes to make the Work available on an Open Access basis (and intends to pay the relevant APC), the terms of reuse of such Open Access shall be governed by a Creative Commons licence – details of these licences and which Creative Commons licence will apply to this Work are set out in our licence referred to above.

Other than as permitted in any relevant BMJ Author's Self Archiving Policies, I confirm this Work has not been accepted for publication elsewhere, is not being considered for publication elsewhere and does not duplicate material already published. I confirm all authors consent to publication of this Work and authorise the granting of this licence.

For peer review only

Challenges in diagnosing common neonatal conditions in Nigeria and Kenya

Aimee Staunton* [1], Helen M Nabwera[1,2], Stephen J Allen[1,2], Olukemi O Tongo[3], Abimbola E Akindolire[3], Isa Abdulkadir[4], V. Chinyere Ezeaka[5], Beatrice N Ezenwa[5], Iretiola B Fajolu[5,6], Zainab O Imam[6], Dominic D Umoru[7], Walter Otieno[8,9], Grace M Nalwa[8,9], Macrine Olwala[9], Alison W Talbert[10], Pauline EA Andang'o[8], Martha K Mwangome[10], Ismaela Abubakar[1], Nicholas D Embleton[11,12], on behalf of the Neonatal Nutrition Network (NeoNuNet)**

1. Liverpool School of Tropical Medicine, Pembroke place, Liverpool, L3 5QA, UK
2. Alder Hey Children's Hospital NHS Trust, E Prescot Rd, Liverpool L14 5AB, UK
3. University College Hospital, Queen Elizabeth Road, Ibadan, Nigeria
4. Ahmadu Bello University Teaching Hospital, Shika, Zaria, Nigeria
5. College of Medicine, University of Lagos, Idi-Araba, Lagos, Nigeria; Lagos University Teaching Hospital, Idi-Araba, Lagos, Nigeria.
6. Lagos State University Teaching Hospital, Ikeja, Lagos, Nigeria
7. Maitama District Hospital, 61 Aguiyi Ironsi St, Maitama, Abuja, Nigeria
8. Maseno University, Maseno, Kenya
9. Jaramogi Oginga Odinga Teaching and Referral Hospital, Jomo Kenyatta Highway Kaloleni Kisumu KE, Central, Kenya
10. KEMRI-Wellcome Trust Research Programme, PO Box 230-80108, Kilifi, Kenya
11. Newcastle University, Newcastle upon Tyne NE1 7RU, UK
12. The Newcastle upon Tyne Hospitals NHS Foundation Trust, High Heaton, NE7 7DN, UK

**Corresponding author, contact: aimee.staunton@ntlworld.com*

***Full Neonatal Nutrition Network members list in Appendix 1*

Short title: Diagnostic challenges for neonatal conditions in Nigeria and Kenya

Key terms: Diagnosis, neonatal conditions, Nigeria, Kenya

Number of tables: 2

Number of figures: 1

For peer review only - http://bmjopen.bmj.com/site/about/guidelines.xhtml

Abstract

Objectives: Accurate and timely diagnosis of common neonatal conditions is crucial for reducing neonatal deaths. In low- and middle-income countries with limited resources, there is sparse information on how neonatal diagnoses are made. The aim of this study was to describe the diagnostic criteria used for common conditions in neonatal units (NNUs) in Nigeria and Kenya.

Methods: Standard case report forms for suspected sepsis, respiratory disorders, birth asphyxia and abdominal conditions were co-developed by the Neonatal Nutrition Network* collaborators. Over 6 months, clinicians completed forms for all admissions to seven NNUs in Nigeria and Kenya.

Results: Amongst 2851 neonatal admissions, 1230 (43.1%) had suspected sepsis, 874 (30.6%) respiratory conditions, 587 (20.6%) birth asphyxia and 71 (2.5%) abdominal conditions. For all conditions, clinical criteria were used consistently across the NNUs with sparse use of laboratory and radiological criteria.

Conclusion: Our findings highlight the reliance on clinical criteria and extremely limited use of diagnostic technologies for common conditions in NNUs in sub-Saharan Africa. This has implications for the management of neonatal conditions which often have overlapping clinical features. Strategies for implementation of diagnostic pathways and investment in affordable and sustainable diagnostics are needed to improve care for these vulnerable infants.

* https://www.lstmed.ac.uk/nnu

For peer review only

What is already known on this topic:

1. The reliable diagnosis of common neonatal conditions is crucial to reduce mortality.

2. Clinicians in LMICs face complex challenges in diagnosing common neonatal illnesses.

What this study adds:

1. Clinicians adopted a syndromic approach to diagnosis with limited access to laboratory and radiological diagnostic technologies.

How this might affect research, practice, or policy:

1. There is a critical need for sustainable and affordable diagnostic tests suitable for use in resource limited settings for neonates and this must be a key priority of future healthcare quality improvement initiatives.

2. Our findings will inform the development and implementation of national plans and integrated context-relevant guidelines to increase access to appropriate diagnostics and improve neonatal care in low- resource settings.

For peer review only

For peer review only - http://bmjopen.bmj.com/site/about/guidelines.xhtml

Background

The majority of the 2.4 million global neonatal deaths (< 28 days) in 2019 were due to three largely preventable or treatable conditions: complications of preterm birth, intrapartum-related events and sepsis (1). Accurate and timely diagnosis of these common conditions is crucial for reducing mortality (1, 2). The clinical assessments and decisions from presentation to diagnosis, referred to as diagnostic pathways, are known to influence outcomes (3). Although access to diagnostics is included in one of the six WHO health system building blocks, there are limited data on diagnostic pathways and how these influence outcomes in neonatal care in low-and middle-income countries (LMICs) (4-6).

In Nigeria and Kenya, concerted actions are required to reduce neonatal mortality (7). Progress in reducing neonatal mortality rates (NMR; the number of deaths occurring 0-<28 days per 1000 live births) has been slow in both countries (8). Between 1990 and 2019, NMR in Nigeria decreased by 28% from 50 to 36 per 1000 and in Kenya by 25% from 28 to 21 per 1000 (1). Consequently, both countries are unlikely to meet the sustainable development goal (SDG 3.2) for neonatal mortality and are thus classified as priority countries in the Countdown to 2030 initiative (9).

In the context of robust health systems, national or regional guidelines may support the standardisation and accuracy of diagnoses as a means of improving care and reducing mortality in highly vulnerable hospitalised infants (10, 11). Whilst both countries have policies on newborn health, only Kenya had national guidelines for the management of common neonatal conditions at the time of the study (12, 13). However, there is limited information on the impact of guidelines in low-resource settings, where challenges with resources for health are greatest (14).

Encouraged by international initiatives to improve access to diagnostics and the sparse information on how common neonatal diagnoses are made (5, 15-17), we sought to describe

For peer review only - http://bmjopen.bmj.com/site/about/guidelines.xhtml

the diagnostic criteria used by clinicians in seven neonatal units (NNUs) in Nigeria and Kenya participating in the Neonatal Nutrition Network (NeoNuNet https://www.lstmed.ac.uk/nnu).

Methods

Design and setting

In this observational study, we collected information from five NNUs in Nigeria and two in Kenya; five providing tertiary level care, two providing district level care and all but one situated in major cities (18). All NNUs provided supportive feeding, intravenous fluids, antibiotics, phototherapy and bubble continuous positive airway pressure (CPAP). Two out of seven NNUs provide free neonatal healthcare services.

Data Collection

Case report forms were developed for birth asphyxia, respiratory disorders, abdominal conditions, and suspected sepsis with reference to international and local guidelines. Respiratory conditions included respiratory distress syndrome, transient tachypnoea of the newborn, pneumonia and meconium aspiration. Abdominal conditions included necrotizing enterocolitis (NEC), dysmotility, septic ileus and focal intestinal perforation. Suspected sepsis was defined as clinical assessment resulting in starting or changing antibiotic treatment. Over a 6-month period between September 2018 and April 2019, clinical staff completed case report forms for all admissions recording a single episode for each condition in each infant. Demographic and clinical information was extracted from the main NeoNuNet database. For out-born infants in whom birthweight was not known, weight on admission was used instead of birthweight. Data clerks entered anonymised data into a password protected REDCap database hosted by the Liverpool School of Tropical Medicine (LSTM) (10, 19).

Patient and Public Involvement

Study participants and the public were not involved in the design or undertaking of the study.

For peer review only

For peer review only - http://bmjopen.bmj.com/site/about/guidelines.xhtml

Data management and analysis

Data was imported to SPSS version 25 (20) for analysis. Categorical variables were presented as frequencies and percentages. Diagnostic criteria were presented using heatmaps (Microsoft Office 16 Excel). Each criterion was classified as either used (with either a positive or negative finding) or not used. Criteria were grouped into clinical, laboratory, clinical/laboratory, and radiological criteria. The 'COUNTIFS' function was used to calculate the proportion of cases in which the criterion was used, and a divergent colour scheme was applied to the heatmap cells.

Results

Over the 6 months' data collection period, 2851 neonates were admitted to the seven NNUs. The majority of newborns were male (1626; 57.1%), most had been delivered in a health facility (2600; 91.2%) and just over half were vaginal unassisted deliveries (1586; 55.7%). Overall, 1261 (45.3%) were preterm (<37 weeks' gestation), 1405 (49.3%) were low birth weight (<2.50kg) and 473 (16.6%) infants died (Table 1). The proportions of very preterm (VPT, <32 weeks' gestation) or very low birth weight (VLBW, <1500g) infants varied considerably across the NNUs (Table 2).

Table 1: Newborn characteristics of 2851 admissions[1]

Variable	
Male gender[2]	1626 (57.1)
Place of delivery	
• Health facility	2600 (91.2)
• Home	165 (5.8)
• Other	86 (3.0)
Mode of delivery[3]	
• Vaginal unassisted	1586 (55.7)
• Caesarean section	1160 (40.7)

For peer review only

• Vaginal assisted/ instrumental	101 (3.5)
Gestational age in weeks[4] Median (IQR)	37.0 (33.0-39.0)
Gestational age category[4]	
• Extreme preterm (<28 weeks)	122 (4.3)
• Very preterm (28-<32 weeks)	378 (13.3)
• Moderately preterm (32-<37 weeks)	761 (26.7)
Birth weight or weight on admission in kg[5] Median (IQR)	2.50 (1.67-31.7)
Birth weight or weight on admission category[5] (n; %)	
• ELBW (<1000g)	116 (4.1)
• VLBW (1000-1499g)	393 (13.8)
• LBW (1500-2499g)	896 (31.5)
Antibiotics given after birth[6]	1294 (45.4)
Final infant outcome[7]	
• Discharged with no morbidities	2170 (76.1)
• Discharged with morbidities	89 (3.1)
• Transferred out	43 (1.5)
• Absconded/ discharged against medical advice	55 (1.9)
• Died	473 (16.6)

Notes

1. Variables are shown as number (%) unless otherwise stated
2. Gender missing in 2
3. Mode of delivery missing in 4
4. Gestational age missing for 68
5. Birthweight and weight on admission missing in 3
6. Prophylactic antibiotics missing in 36
7. Final infant outcome missing in 22

For peer review only

Table 2: Admission characteristics and frequency of common conditions according to neonatal unit[1]

**Condition**	Kenya		Nigeria					**Total**
	1	2	3	4	5	6	7	
Total admissions	291	613	181	732	384	238	412	2851
Extreme or very preterm (<32 weeks' gestation)	52 (17.9)	94 (16.3)	9 (5.1)	124 (17.4)	142 (37.1)	69 (29.1)	10 (2.5)	500 (27.8)[2]
Extreme or very low birth/admission weight (<1500g)	60 (20.6)	107 (17.5)	13 (3.3)	104 (14.2)	128 (33.3)	69 (29.0)	28 (6.8)	509 (28.5)[3]
Suspected sepsis	132 (45.4)	421 (68.7)	68 (37.6)	405 (53.3)	49 (12.8)	46 (19.3)	109 (26.5)	1230 (43.1)
Respiratory conditions	78 (26.8)	364 (59.4)	19 (10.5)	210 (28.7)	18 (4.7)	162 (68.1)	23 (5.6)	874 (30.7)
Birth asphyxia	48 (16.5)	189 (30.8)	20 (11.0)	190 (26.0)	41 (10.7)	44 (18.5)	55 (13.3)	587 (20.6)
Abdominal conditions	3 (1.0)	4 (0.7)	6 (3.3)	32 (4.4)	3 (0.8)	14 (5.9)	9 (2.2)	71 (2.5)

Notes:

1. Data are shown as number (%)
2. Gestation was missing for 35 infants in unit 2, 5 in unit 3, 20 in unit 4, 1 each in units 5 and 6 and 6 in unit 7
3. Birth/admission weight was missing for 2 infants in unit 2 and 1 infant in unit 7

For peer review only - http://bmjopen.bmj.com/site/about/guidelines.xhtml

Diagnostic criteria

Overall, forms were completed for 1230 (43.1%) newborns diagnosed with suspected sepsis, 874 (30.6%) with respiratory conditions, 587 (20.6%) with birth asphyxia and 71 (2.5%) with abdominal conditions (Table 2). The frequency of these diagnoses varied considerably between NNUs including those with similar proportions of VPT or VLBW infants. Overall, across all NNUs, diagnoses were based mainly on clinical criteria and a full blood count with limited use of other laboratory assays or radiological investigations (*Fig 1a-1d*).

For the diagnosis of suspected neonatal sepsis, clinical criteria were used uniformly in all NNUs (*Fig 1a*). The exceptions were hypotension where use varied from 0% to 98% (Appendix 3a). Assessment of glucose intolerance, often using a point of care test, and parameters from a full blood count were the most used laboratory criteria. However, other laboratory parameters requiring biochemical analyses and microbiology were used much less frequently overall.

Similarly, for respiratory conditions, clinical criteria were used uniformly across NNUs (*Fig 1b; appendix 3b*). In marked contrast to the limited use of laboratory analyses, the use of chest Xray appeared to be good overall but with some variability between NNUs.

The diagnosis of birth asphyxia was based on clinical evidence of encephalopathy and exclusion of other aetiologies. Evidence of multiorgan dysfunction was also used although this may have been based on the clinical components (acute kidney injury, respiratory distress, circulatory collapse and disseminated intravascular coagulation) rather than laboratory analyses. In contrast, use of cranial ultrasound scan varied widely ranging from 0-84% (Fig 1c; appendix 3c).

For the infants with an abdominal condition, of which about half were diagnosed as NEC (36/71, 50.7%), a range of clinical criteria were used in nearly all cases in most NNUs. Basic

For peer review only - http://bmjopen.bmj.com/site/about/guidelines.xhtml

laboratory criteria derived from a full blood count were also frequently used but use of parameters of metabolic acidosis was more variable. Overall, radiology criteria were used less frequently (*Fig 1d).*

Discussion

This study enabled us to explore diagnostic pathways and identify key challenges in the diagnosis of common neonatal conditions in the context of limited resources. Clinicians were generally constrained to a symptoms-based, clinical orientated diagnostic approach. This is consistent with the widely available WHO Pocket Book which encourages a syndromic approach and is a common source of diagnostic guidelines (21). However, the Pocket Book is targeted at first level referral hospitals, whereas greater access to diagnostics is required for higher-level care.

The consistent use of a wide range of clinical criteria across the NNUs was a positive finding suggesting uniformity in clinical practice. This is likely supported by use of national neonatal clinical guidelines although these were available in Kenya only. National paediatric diagnostic guidelines have been found to optimise practice in high-income settings; however, this may be more challenging in LMICs where diagnostic resources and skilled workforce remain inadequate making consistent adherence to clinical guidelines difficult (22, 23). This is further complicated by high staff turnover due to poor working conditions, healthcare strikes and political instability (24, 25).

In marked contrast with clinical criteria, the limited use of laboratory investigations beyond a full blood count, shows that there were challenges implementing standardised diagnostic pathways. There was obvious overlap between conditions, so that investigations such as blood culture were used infrequently in both suspected sepsis and respiratory illness. Although

For peer review only - http://bmjopen.bmj.com/site/about/guidelines.xhtml

some laboratory procedures were available in individual hospitals, in our experience, this is often short-term due to dependency on donor or research support. The limited use of laboratory investigations within the NNUs may be due to a lack of affordable and quality assured laboratory and specimen transport services, which are common barriers in LMICs (5, 26). This is likely to have implications on the accuracy of diagnoses, since a syndromic diagnostic approach has been linked to lower specificity, thereby resulting in more false positive cases (27). The lack of laboratory diagnostic support in our study would likely result in the overdiagnosis of neonatal sepsis. A direct consequence is the overuse of antibiotics in this population, which could drive antimicrobial resistance(28). Use of laboratory investigations to complement clinical assessment, as well as antibiotic stewardship programmes, would likely help (29).

Respiratory conditions such as respiratory distress syndrome were typically diagnosed using clinical criteria but also supported by chest X rays which are critically important in distinguishing the cause of respiratory distress, which includes a number of pathologies with overlapping clinical signs (30). The diagnosis of birth asphyxia was also based mainly on clinical criteria and with limited use of cranial ultrasound. In addition to diagnosis, laboratory support with determining acid-base balance and blood electrolytes is required in the selection of infants for treatment approaches such as therapeutic hypothermia and monitoring treatment response (31).

In contrast to the use of X ray in respiratory conditions, abdominal Xray, a key investigation for the diagnosis of conditions such as NEC, was only used consistently in one unit. The limited use may be due to more severe illness in infants with suspected NEC and the lack of mobile X ray machines. Previous research in LMIC settings has reported that diagnosis is impaired by a lack of abdominal X rays, since even when X ray machines are available and functioning, they are often too expensive for patients and families to afford (32). This is likely here since

For peer review only - http://bmjopen.bmj.com/site/about/guidelines.xhtml

only two out of the seven NNUs provide free of charge neonatal health services. This means that diagnosis is very difficult for conditions such as NEC where specific X ray findings are critically important (33).

Given the consistent use of clinical criteria across the NNUs, the limited use of laboratory and radiological investigations likely contributed to the marked differences between units in the frequencies of diagnoses. This would appear to be less likely due to differences in case mix, since variability in the frequency of diagnoses occurred between NNUs with similar proportions of small and preterm newborns in whom these conditions are common. These challenges highlight the need for sustainable and affordable diagnostic tests suitable for use in resource limited settings for accurate and timely diagnosis and to reliably assess the frequency of the main causes of neonatal mortality. This should include point-of-care diagnostic tests designed for use in LMICs which can provide rapid results and reduce the workload for laboratory services (34, 35).

Limitations

Most of the network NNUs were urban and tertiary level which are often inaccessible to disadvantaged families due to higher access costs. It is therefore likely that the families in our study could better afford some investigations than many in Nigeria and Kenya limiting the generalisability of our findings. Differences in case mix between the NNUs and in the experience of clinicians in making diagnoses limit the interpretation of our findings (10, 36).

Conclusions

Our findings demonstrate that diagnostic pathways for common conditions in NNUs in Nigeria and Kenya were hindered by the limited use of laboratory and radiological technologies. Improving capacity to diagnose common conditions rapidly and reliably in hospitalised newborns should be a key priority of future healthcare quality improvement initiatives. The

For peer review only - http://bmjopen.bmj.com/site/about/guidelines.xhtml

development of national plans to increase the availability, accessibility, and affordability of point of care and other diagnostic investigations, is paramount since the need for out-of-pocket health spending hinders compliance to clinical guidelines (5). Our findings will inform the development and implementation of integrated context-relevant best practice guidelines for neonatal care in low- resource settings.

Acknowledgements

We would like to thank our colleagues who contributed to the clinical care and collection of data at the Network neonatal units. We would also like to thank colleagues at the Nigerian Society of Neonatal Medicine, the Kenya Paediatric Association and the Ministries of Health in Nigeria and Kenya, who provided us with support and advice as we were setting up this study. We thank the mothers and families immensely for participating in this study with their infants. This study is published with the permission of the Director of Kenya Medical Research Institute.

Contributorship statement

Stephen J Allen conceived the study; all authors designed the study; Aimee Staunton analysed the data; Aimee Staunton, Stephen J Allen and Helen M Nabwera prepared the manuscript. Aimee Staunton is the corresponding author responsible for submission, peer review and publication processes. All authors read, helped to critically revise and approved the final manuscript version. All authors agree to be accountable for the accuracy and integrity of the manuscript and to answer any questions in relation to the work. The work was completed on behalf of the Neonatal Nutrition Network, all members of which are listed in Appendix 1. Members not listed as authors are non-author contributors who helped with the initial study proposal.

For peer review only - http://bmjopen.bmj.com/site/about/guidelines.xhtml

Open access

This is an open access article distributed in accordance with the Creative Commons Attribution 4.0 Unported (CCBY4.0) license, which permits others to copy, redistribute, remix, transform and build upon this work for any purpose, provided the original work is properly cited, a link to the licence is given, and indication of whether changes were made.

Data sharing

A minimal anonymized dataset from the Network has been shared (15). Requests for access to further data should be addressed to the corresponding author.

Ethics approval

Ethical approval was granted by the Research Ethics Committees at LSTM (18–0210), Lagos University Teaching Hospital Health REC (AMD/DCST/HREC/APP/2514), the Jaramogi Oginga Odinga Teaching and Referral Hospital (ERC.IB/VOL.1/510), University College Hospital Ibadan (UI/EC/18/0446), Massey Street Children's Hospital (LSHSC/2222/VOL.VI[B]/185), Ahmadu Bello University Teaching Hospital (ABUTH/HZ/HREC/D37/2018), Maitama District Hospital (FHREC/2018/01/108/19-09-18), the Kenya Medical Research Institute-Scientific and Ethics Review Unit (KEMRI/SERU/CGMR-C/120/3740) (10) (Appendices 2a-2b).

Funding

This project was funded by a grant from the MRC Confidence in Global Nutrition and Health Research scheme (grant reference MC_PC_MR/R019789/1); the funders had no role in the design or conduction of the study.

Conflict of Interests Statement

None to declare.

For peer review only - http://bmjopen.bmj.com/site/about/guidelines.xhtml

References

1. United Nations Inter-agency Group for Child Mortality Estimation (UN IGME). Levels and Trend in Child Mortality Report. 2020.
2. Healthy Newborn Network. Explore Countries- Nigeria. healthynewbornnetwork.org.2017 [Available from: https://www.healthynewbornnetwork.org/country/nigeria/.
3. National Academies of Sciences Engineering and Medicine. Improving diagnosis in health care: National Academies Press; 2015.
4. World Health Organization. Everybody's business-strengthening health systems to improve health outcomes: WHO's framework for action. 2007.
5. Fleming K, Horton S, Wilson L, et al. The Lancet Commission on diagnostics: transforming access to diagnostics. *The Lancet*. 2021;398:1997-2050.
6. Pius S, Bello M. Neonatal Septicaemia in Poor Resource Settings. *Pediatric Infect Dis*. 2017;2:2537-0282.
7. The World Bank. World Bank Country and Lending Groups. worldbank.org.2018 [Available from: https://datahelpdesk.worldbank.org/knowledgebase/articles/906519.
8. World Health Organization. Progress Report: Reaching every newborn national 2020 milestones: country progress, plans and moving forward. 2018.
9. Boerma T, Requejo J, Victora C, et al. Countdown to 2030: tracking progress towards universal coverage for reproductive, maternal, newborn, and child health. *The Lancet*. 2018;391:1538-1548.
10. Nabwera HM, Wang D, Tongo OO, et al. Burden of disease and risk factors for mortality amongst hospitalized newborns in Nigeria and Kenya. *PloS one*. 2021;16: e0244109.
11. Bhutta ZA, Giuliani F, Haroon A, et al. Standardisation of neonatal clinical practice. *BJOG*. 2013;120:56-63.
12. Federal Ministry of Health. Nigeria Every Newborn Action Plan: A plan to end preventable newborn deaths in Nigeria. 2016.
13. Ministry of Health. Health ministry launches policy to accelerate gains in child health. 2018 [Available from: http://www.health.go.ke/health-ministry-launches-policytoaccelerate-gains-in-child-health-nairobi-kenya-november-19-2018/.
14. Ballot DE, Chirwa TF, Cooper PA. Determinants of survival in very low birth weight neonates in a public sector hospital in Johannesburg. *BMC Pediatr.* 2010;10:30.

For peer review only - http://bmjopen.bmj.com/site/about/guidelines.xhtml

15. Engel N, Wachter K, Pai M, et al. Addressing the challenges of diagnostics demand and supply: insights from an online global health discussion platform. *BMJ Glob Health*. 2016;1:e000132.
16. Welcome MO. The Nigerian health care system: Need for integrating adequate medical intelligence and surveillance systems. *J Pharm Bioallied Sci*. 2011;3:478.
17. English M, Gathara D, Nzinga J, et al. Lessons from a Health Policy and Systems Research programme exploring the quality and coverage of newborn care in Kenya. *BMJ Glob Health*. 2020;5:e001937.
18. Liverpool School of Tropical Medicine. About the Neonatal Nutrition Network [Available from: https://www.lstmed.ac.uk/research/departments/clinicalsciences/neonatal-nutritionnetwork/about-the-neonatal-nutrition.
19. Harris PA, Taylor R, Minor BL, et al. The REDCap consortium: Building an international community of software platform partners. *J Biomed Inform.* 2019;95:103208.
20. IMB Corp. IBM SPSS statistics for Windows, version 20.0. 2011.
21. World Health Organization. Pocket book of hospital care for children: guidelines for the management of common childhood illnesses. 2013.
22. Akenroye AT, Baskin MN, Samnaliev M, et al. Impact of a bronchiolitis guideline on ED resource use and cost: a segmented time-series analysis. *Pediatrics*. 2014;133:227-234.
23. Lopez AA, Aslanova R, Bridger N, et al. Antibiotic Use for Inpatient Bronchiolitis: Did National Guidelines Impact Practice at a Pediatric Hospital? *Hospital Pediatrics*. 2020;10:147-152.
24. Irimu G, Ogero M, Mbevi G, et al. Tackling health professionals' strikes: an essential part of health system strengthening in Kenya. *BMJ Glob Health*. 2018;3:e001136.
25. UNICEF. Country Office Annual Report- Kenya. unicef.org; 2018.
26. Majors CE, Smith CA, Natoli ME. Point-of-care diagnostics to improve maternal and neonatal health in low-resource settings. *Lab on a Chip*. 2017;17:3351-3387.
27. Mabey D, Vos T. Syndromic approaches to disease management. *The Lancet*. 1997;349:26-28.
28. Ellington LE, Gilman RH, Chavez MA, et al. Lung ultrasound as a diagnostic tool for radiographically-confirmed pneumonia in low resource settings. *Respiratory Medicine*. 2017;128:57-64.
29. Camacho-Gonzalez A, Spearman PW, Stoll BJ. Neonatal infectious diseases:

For peer review only - http://bmjopen.bmj.com/site/about/guidelines.xhtml

evaluation of neonatal sepsis. *Pediatric Clinics*. 2013;60:367-389.

30. Gallacher DJ, Hart K, Kotecha S. Common respiratory conditions of the newborn. *Eur Respir* J. 2016;12:30-42.
31. Lemyre B, Chau V. Hypothermia for newborns with hypoxic-ischemic encephalopathy. *Paediatrics & child health*. 2018;4:285-295.
32. Filkins M, Halliday S, Daniels B, et al. Implementing Diagnostic Imaging Services in a Rural Setting of Extreme Poverty: Five Years of X-ray and Ultrasound Service Delivery in Achham, Nepal. *Journal of Global Radiology*. 2015;1:1-2.
33. Gephart SM, Gordon PV, Penn AH, et al. Changing the paradigm of defining, detecting, and diagnosing NEC: Perspectives on Bell's stages and biomarkers for NEC. *Semin Pediatr Surg*. 2018;27:3-10.
34. Schito M, Peter TF, Cavanaugh S, et al. Opportunities and Challenges for CostEfficient Implementation of New Point-of-Care Diagnostics for HIV and Tuberculosis. *J Infect Dis*. 2012;205:169-180.
35. Drain PK, Hyle EP, Noubary F, et al. Diagnostic point-of-care tests in resourcelimited settings. *Lancet Infect Dis*. 2014;14:239-249.
36. Murphy GAV, Gathara D, Abuya N, et al. What capacity exists to provide essential inpatient care to small and sick newborns in a high mortality urban setting? - A cross sectional study in Nairobi City County, Kenya. *PloS one*. 2018;13:e0196585.

For peer review only - http://bmjopen.bmj.com/site/about/guidelines.xhtml

Figure 1: Heatmaps showing the diagnostic criteria used for common neonatal conditions

Figure 1a: Suspected neonatal sepsis

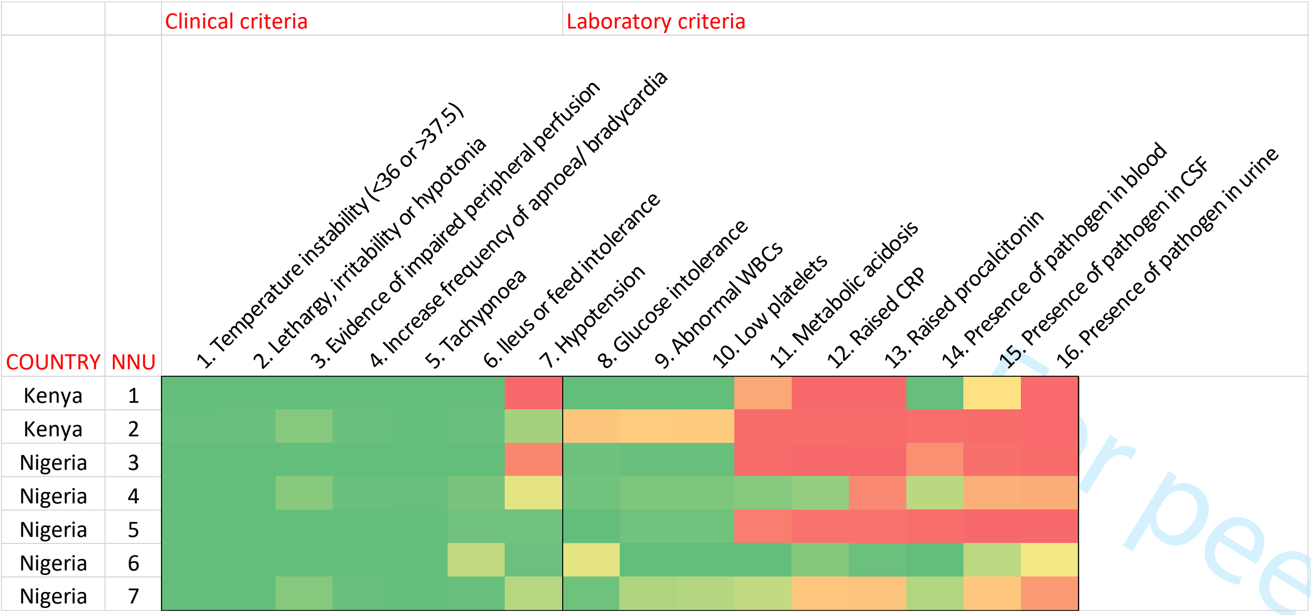

Figure 1c: Birth asphyxia

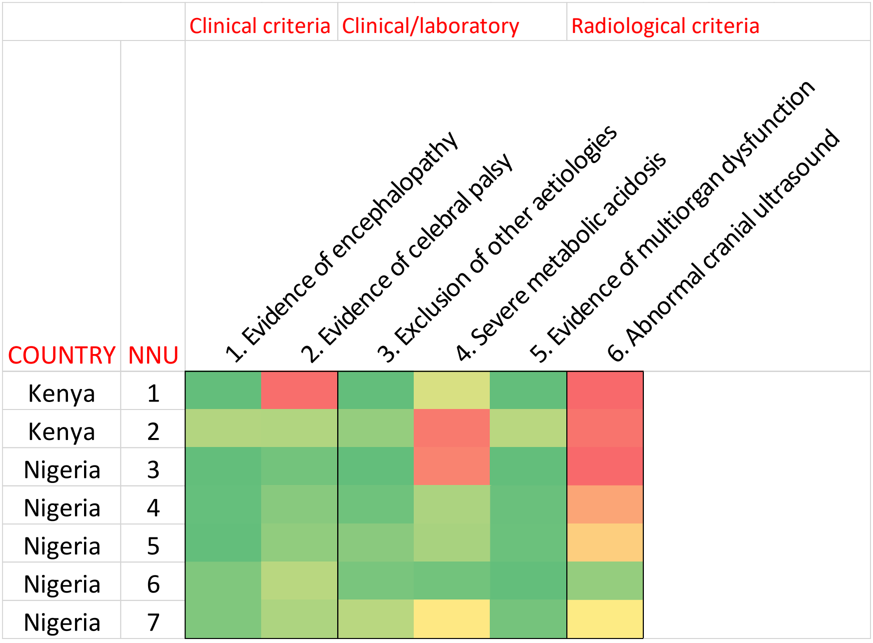

Figure 1b: Respiratory conditions

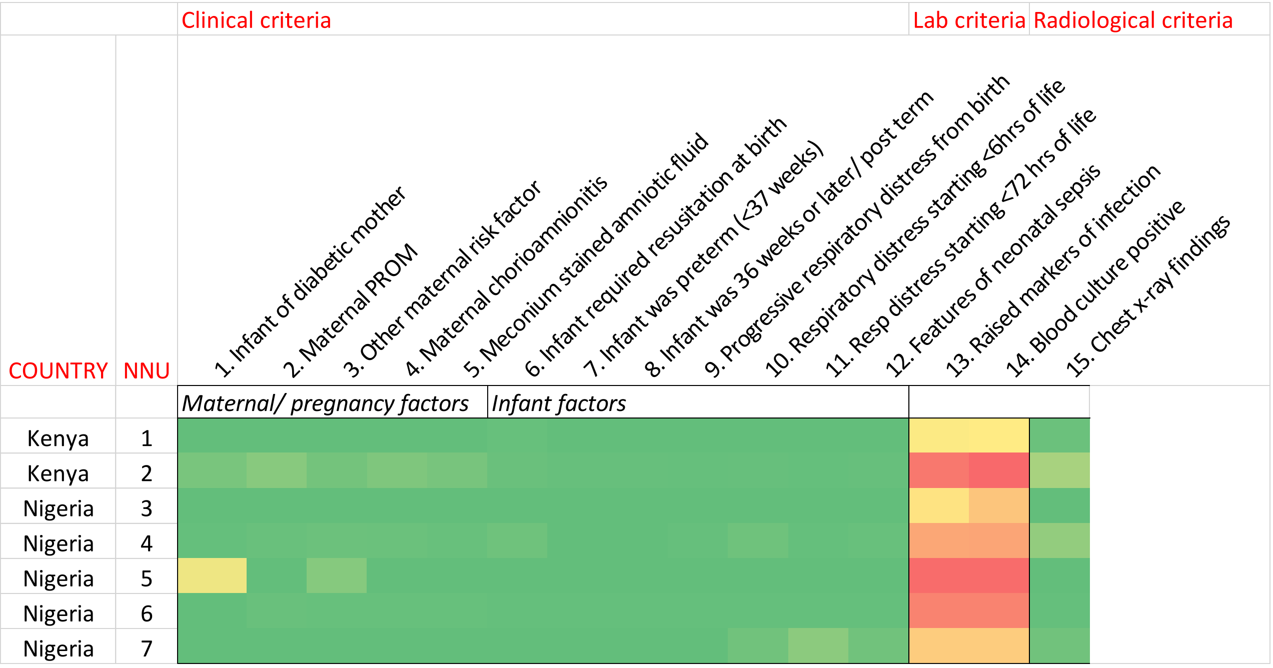

Figure 1d: Abdominal conditions

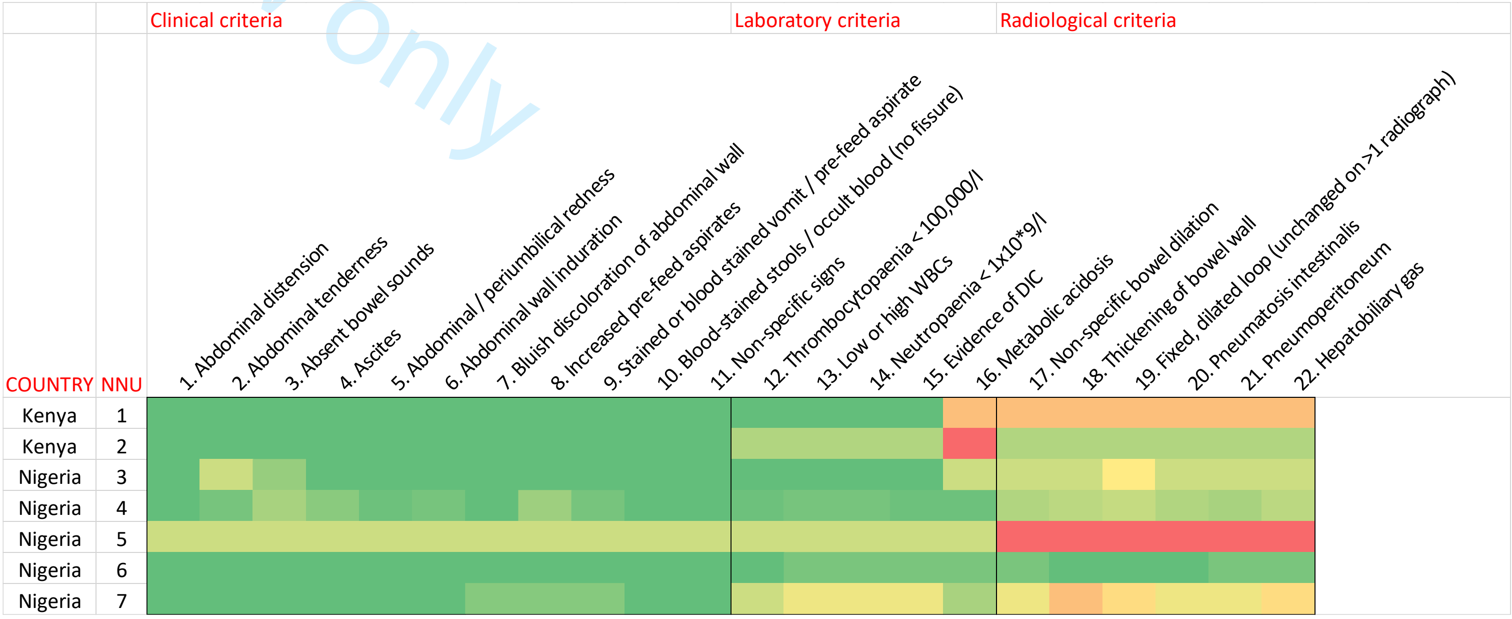

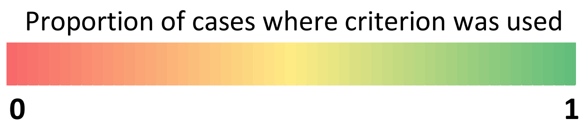

For peer review only

Appendices

Appendix 1: *Neonatal Nutrition Network Members*

Isa Abdulkadir (Ahmadu Bello University, Nigeria); Ismaela Abubakar (LSTM, UK); Abimbola E Akindolire (College of Medicine, University of Ibadan, Nigeria); Olusegun Akinyinka (College of Medicine, University of Ibadan, Nigeria); Stephen J Allen (LSTM, UK); Pauline EA Andang'o (Maseno University, Kenya); Graham Devereux (LSTM, UK); Chinyere Ezeaka (Lagos University Teaching Hospital, Nigeria); Beatrice N Ezenwa (Lagos University Teaching Hospital, Nigeria); Iretiola B Fajolu (Lagos University Teaching Hospital, Nigeria); Zainab O Imam (Lagos State University Teaching Hospital, Nigeria); Kevin Mortimer (LSTM, UK); Martha K Mwangome (KEMRI Wellcome Trust Research Programme, Kenya); Helen M Nabwera (LSTM, UK); Grace M Nalwa (Jaramogi Oginga Odinga Teaching and Referral Hospital, Kenya & Maseno University, Kenya); Walter Otieno (Jaramogi Oginga Odinga Teaching and Referral Hospital, Kenya & Maseno University, Kenya); Alison W Talbert (KEMRI Wellcome Trust Research Programme, Kenya); Nicholas D Embleton (Newcastle University, UK); Olukemi O Tongo (College of Medicine, University of Ibadan, Nigeria); Dominic D Umoru (Maitama District Hospital, Nigeria); Janneke van de Wijgert (University of Liverpool, UK); Melissa Gladstone (University of Liverpool, UK).

For peer review only

Appendix 2a: Ethical approval details from collaborating institutions

**Institution**	**Protocol number**
**Liverpool School of Tropical Medicine Research Ethics Committee (REC)**	18-0210
**The Lagos University Teaching Hospital Health Research Ethics Committee**	AMD/DCST/HREC/APP/2514
**The research and ethics committees at The Jaramogi Oginga Odinga Teaching and Referral Hospital**	ERC.IB/VOL.1/510
**University College Hospital Ibadan**	UI/EC/18/0446
**Massey Street Children's Hospital**	LSHSC/2222/VOL.VI^{B}/185
**Ahmadu Bello University Teaching Hospital**	ABUTH/HZ/HREC/D37/2018
**Maitama District Hospital**	FHREC/2018/01/108/19-09-18
**The Kenya Medical Research Institute-Scientific and Ethics Review Unit**	KEMRI/SERU/CGMR-C/120/3740

For peer review only

Appendix 2b: Confirmation of data access request from KEMRI- Wellcome Trust

1. REQUESTOR DETAILS					
**Name**	Aimee Staunton				
**Contact details: address, phone and email**	97 Aigburth Road Liverpool L17 4JU Liverpool School of Tropical Medicine	**Phone**	07530351521	**Email**	264266@lstmed.ac.uk

2. STUDY DETAILS			
**Title of Research Project**	Describing the diagnostic pathways for common neonatal conditions across seven neonatal units in sub-Saharan Africa	**Start & end dates**	June 2020- September 2020
**Ethical approval sought / planned (with dates)**	Ethics application covered by existing approved proposal:<br><br>Title of proposal: Improving the survival, growth and development of low birth weight newborns through better nutrition<br>LSTM Research Ethics Committee, reference number: 18-0210<br>PI: Prof Stephen Allen<br>Date of approval: 4th July 2018<br>End date for data collection: 31st August 2019		

3. INVESTIGATORS

Names of all investigators who will have access to data (begin with Principal Investigator)* include names of KWTRP collaborators where applicable.

Name	Role	Institution
Aimee Staunton	Principal Investigator (MSc student)	LSTM
Helen Nabwera	Project Supervisor	LSTM
Stephen Allen	Project Supervisor	LSTM

4. BRIEF DESCRIPTION TYPE OF DATA REQUIRED

The dataset collected by NeoNuNet to record diagnostic pathways, comprising all admissions to the seven network neonatal units over a six-month period between August 2018 and May 2019. This includes 2280 neonates in total.

Title: Improving the survival, growth and development of low birth weight newborns through better nutrition

PI: Dr Martha Mwangome

KEMRI/SERU/CGMT-C/120/3740

5. OBJECTIVES AND STUDY DETAILS INCLUDING TIME FRAME

1. To describe the population of neonates and the causes of admission to secondary and tertiary level neonatal units in sub-Saharan Africa using secondary data collected from seven neonatal units in Nigeria and Kenya by September 2020.

2. To describe how common neonatal conditions (asphyxia, respiratory disorders, abdominal conditions/ necrotizing enterocolitis and neonatal sepsis) are diagnosed in the seven neonatal units in Nigeria and Kenya by September 2020.

3. To identify any gaps or inaccuracy in the diagnostic pathways for common neonatal conditions in the seven neonatal units and explore the association with neonatal outcomes by September 2020.

6. BRIEF DESCRIPTION OF ANALYSIS PLANNED WITHIN KIDMS DATA ***specify if study specific data not KIDMS data**

Objective 1: To fulfil this objective, data will be analysed using SPSS to describe the population of neonates. The relative percentages of the different diagnoses will be calculated. Descriptive statistics for variables such as gestation, sex and birthweight will be generated and basic hypothesis testing will be carried out. This will help to describe the biodemographic characteristics of the population being studied.

Objective 2: To fulfil this objective, the diagnostic pathways that lead to the final diagnosis (birth asphyxia, respiratory disorders, abdominal conditions/ necrotizing enterocolitis (NEC) or neonatal sepsis) will be analysed. This will be

Conditions and limitations for data sharing

You understand and acknowledge that custodianship of the Data is held jointly by the Programme and the Ministry of Health in Kilifi, and will be retained by the Programme and the Ministry of Health in Kilifi in case of any data transfer to a Recipient.
You understand and acknowledge that the Data is experimental in nature, and that access to the Data is provided without any representations or warranties of any kind in relation to the Database or the Data.
You will only access the Database and use the Data for the Agreed Purpose and shall not use the Data in such a way that damage or distress is or is reasonably likely to be caused to any Data Subjects.
The Data relates directly to individual Data Subjects and is strictly confidential. Data from subjects will be shared in a fully anonymised form without access to personal identifiers, including names and/or specialised roles. Geographic identifiers for subjects will routinely be aggregated to the level of the sub location or similarly de-identified. Consideration of requests for data with greater granularity will be informed by the local importance of the research question and the ability to protect the confidentiality of individuals and communities.
You shall only disclose the Data to your Data Users. You shall take all reasonable measures to ensure that your Data Users shall not make copies of the whole or any part of the Data without your consent, and shall keep a written record of any such copies sufficient to permit you to fulfil your obligations under clauses 3 and 7 of this Agreement. You shall not transfer or disclose any part of the Data to any other person or body.
You understand and acknowledge that the Data is protected by copyright and other intellectual property rights.
You agree at all times to keep strictly confidential, and ensure that the Data Users keep confidential the information and Data pertaining to Data Subjects. In particular, You undertake not to use, or attempt to use, or permit anyone other than the Data Users to use the Data on its own or in conjunction with other data, to seek to discover the identity of any Data Subjects, to compromise or otherwise infringe the confidentiality of information on Data Subjects and their right to privacy.
You accept that the Data is protected by and subject to national and international laws, and that You are responsible for ensuring compliance with any applicable laws.
You agree that you will submit a summary report and any other information reasonably requested to the KEMRI Wellcome Trust Research Programme for the purposes of monitoring data use on the understanding that such information, data, results, and conclusions contained within will be treated as confidential information belonging to the Recipient.
This agreement is not transferable and You may not purport to assign it (in whole or in part) without the written consent of the KEMRI Wellcome Trust Research Programme.
If You commit a material breach of this Agreement or for any persistent breach of this Agreement, the KEMRI Wellcome Trust may terminate this Agreement immediately by notice in writing, without prejudice to its accrued rights and remedies.
You accept that it may be necessary for the KEMRI Wellcome Trust Research Programme to alter the terms of this Agreement from time to time in order to address new concerns. In this event, the KEMRI Wellcome Trust Research Programme will contact You to inform You of any changes and You agree that Your continued use of the Data shall be dependent on the parties entering into a variation of the Agreement.
If requested to do so by the Data Governance Committee, the KEMRI Wellcome Trust Research Programme may terminate this Agreement at any time by giving one month's notice in writing to You.
Your duty to protect the confidentiality of the Data received under this agreement shall survive termination of this Agreement and shall continue in full force and effect indefinitely.
Upon termination of this Agreement you will permanently delete all copies of the Data from your computer systems and storage devices and will ensure that all Your Data Users shall also permanently delete such copies. You may retain aggregate information on the proviso that such aggregate information does not allow any individuals or groups of individuals who are the subject of the Data to be identified with reasonable effort.
You may publish Your results arising from the use of the Data for the Agreed Purpose providing the Data itself is not disclosed. Aggregate or generic information generated from the Data may be published on the provisos that: i) such aggregate or generic information does not allow Data Subjects or groups of Data Subjects to be identified with reasonable effort; ii) no damage or distress is or is reasonably likely to be caused to any Data Subjects or groups of Data Subjects; iii) the Data will not be used in any way that could reasonably be expected to lead to ethnic stigmatisation; and iv) no attempt will be made to identify the Data Subjects.
You agree to acknowledge the KEMRI Wellcome Trust Research Programme or appropriate Programme researchers (via authorship or an acknowledgment statement) in any work based in whole or part on the Data in the following way :*"This paper/publication has used data and/or biological samples provided by the KEMRI Wellcome Trust Research programme in accordance with the consent provided by participants and approved by the KEMRI Ethics Review Committee"*.
You agree that if your application is approved, information about the proposed research use can be posted on the KEMRI Wellcome Trust Research Programme's public website. The information may include Your name and institution, the title of the project, and a non-technical summary of the research question.
You recognise that nothing in this Agreement shall operate to transfer to You any intellectual property rights relating to the Data. You have the right to develop intellectual property based on comparisons with Your own data.

Definitions in the agreement above:
"Recipient" means the principal researcher named above; "Recipient Institution" means the organisation named above at which the Recipient is employed, affiliated or enrolled; "Data Users" means those officers, employees and students of the Recipient Institution, who work directly with the Recipient and have a need to use the Data for the performance of their work with respect to the Agreed Purpose, and have agreed to comply with this Agreement, as named in the application; "Database" the KIDMS database containing clinical and demographic and health surveillance data on Data Subjects; "Data Subjects" the individuals who have contributed their data to the Database; "Data" the data within the Database; "Agreed Purpose" means the medical research purpose(s) approved by the Data Governance Committee in writing; "Publications" means, without limitation, articles published in print journals, electronic journals, reviews, books, posters and other written and verbal presentations of research.

Statement of agreement: I agree to the above conditions and limitations for sharing data as requested in this application:

Name:____AIMEE STAUNTON________________________
Date:____04/06/20____________________
Signature:____[signature]____________________

Signed on behalf of KEMRI-Wellcome Trust Research Programme:

....

Title:...Professor Philip Bejon...

Position:...Programme Executive Director...

Signature: ....

Date:.......29/Jun/2020...

For peer review only

Appendix 3: Data from heatmaps in Figures 1-4 showing diagnostic criteria use in common neonatal conditions.

3a. Suspected sepsis

For peer review only

NEONATAL SEPSIS

	CRITERIA NUMBER															
NNU	1.	2.	3.	4.	5.	6.	7.	8.	9.	10.	11.	12.	13	14.	15.	16.
1	1.00	1.00	1.00	1.00	1.00	1.00	0.00	1.00	1.00	1.00	0.24	0.00	0.00	0.99	0.46	0.01
2	0.99	0.98	0.89	0.98	1.00	0.98	0.79	0.35	0.38	0.38	0.02	0.01	0.01	0.02	0.01	0.01
3	1.00	1.00	1.00	1.00	1.00	1.00	0.12	0.97	0.99	0.99	0.01	0.00	0.00	0.15	0.03	0.01
4	1.00	1.00	0.88	0.98	0.99	0.93	0.59	0.96	0.92	0.92	0.89	0.84	0.13	0.72	0.27	0.26
5	1.00	1.00	1.00	1.00	1.00	0.96	0.96	1.00	0.96	0.96	0.08	0.04	0.04	0.02	0.00	0.00
6	1.00	1.00	1.00	1.00	1.00	0.70	0.98	0.59	1.00	1.00	1.00	0.89	0.98	1.00	0.72	0.54
7	1.00	1.00	0.89	0.99	1.00	0.99	0.73	0.96	0.76	0.74	0.70	0.36	0.35	0.75	0.37	0.19

3b. Respiratory conditions

RESPIRATORY CONDITIONS															
	CRITERIA NUMBER														
NNU	1	2.	3.	4.	5.	6.	7.	8.	9.	10.	11.	12.	13.	14.	15.
1	1.00	1.00	1.00	1.00	1.00	0.99	1.00	1.00	1.00	1.00	1.00	1.00	0.51	0.50	0.97
2	0.93	0.88	0.95	0.91	0.93	0.98	0.99	0.99	0.99	0.99	0.99	0.99	0.10	0.04	0.78
3	1.00	1.00	1.00	1.00	1.00	1.00	1.00	1.00	1.00	1.00	1.00	1.00	0.47	0.37	1.00
4	1.00	0.98	0.97	0.98	0.99	0.96	1.00	1.00	0.99	0.96	1.00	0.99	0.26	0.26	0.85
5	0.56	1.00	0.89	1.00	1.00	1.00	1.00	1.00	1.00	1.00	1.00	1.00	0.06	0.06	1.00
6	1.00	0.98	0.99	0.99	0.99	0.99	0.99	0.99	0.99	0.99	0.99	0.99	0.14	0.14	0.99
7	1.00	1.00	1.00	1.00	1.00	1.00	1.00	1.00	1.00	0.96	0.87	0.96	0.39	0.39	0.96

For peer review only

3c. Birth asphyxia

BIRTH ASPHYXIA						
	CRITERIA NUMBER					
NNU	1.	2.	3.	4.	5.	6.
1	1.00	0.02	1.00	0.63	1.00	0.00
2	0.75	0.75	0.84	0.07	0.72	0.04
3	1.00	0.95	1.00	0.10	1.00	0.00
4	0.99	0.88	0.96	0.77	0.98	0.23
5	1.00	0.85	0.88	0.78	0.98	0.39
6	0.91	0.73	0.93	0.95	1.00	0.84
7	0.91	0.76	0.73	0.49	0.95	0.51

For peer review only

3d. Abdominal conditions

ABDOMINAL CONDITIONS																						
	CRITERIA NUMBER																					
NNU	1.	2.	3.	4.	5.	6.	7.	8.	9.	10.	11.	12.	13.	14.	15.	16.	17.	18.	19.	20.	21.	22
1	1.00	1.00	1.00	1.00	1.00	1.00	1.00	1.00	1.00	1.00	1.00	1.00	1.00	1.00	1.00	0.33	0.33	0.33	0.33	0.33	0.33	0.33
2	1.00	1.00	1.00	1.00	1.00	1.00	1.00	1.00	1.00	1.00	1.00	0.75	0.75	0.75	0.75	0.00	0.75	0.75	0.75	0.75	0.75	0.75
3	1.00	0.67	0.83	1.00	1.00	1.00	1.00	1.00	1.00	1.00	1.00	1.00	1.00	1.00	1.00	0.67	0.67	0.67	0.50	0.67	0.67	0.67
4	1.00	0.94	0.78	0.88	0.97	0.94	1.00	0.81	0.94	1.00	1.00	0.97	0.94	0.94	0.97	0.97	0.75	0.72	0.69	0.75	0.78	0.72
5	0.67	0.67	0.67	0.67	0.67	0.67	0.67	0.67	0.67	0.67	0.67	0.67	0.67	0.67	0.67	0.67	0.00	0.00	0.00	0.00	0.00	0.00
6	1.00	1.00	1.00	1.00	1.00	1.00	1.00	1.00	1.00	1.00	1.00	1.00	0.93	0.93	0.93	0.93	0.93	1.00	1.00	1.00	0.93	0.93
7	1.00	1.00	1.00	1.00	1.00	1.00	0.89	0.89	0.89	1.00	1.00	0.67	0.56	0.56	0.56	0.78	0.56	0.33	0.44	0.56	0.56	0.44

For peer review only

The RECORD statement – checklist of items, extended from the STROBE statement, that should be reported in observational studies using routinely collected health data.

	Item No.	STROBE items	Location in manuscript where items are reported	RECORD items	Location in manuscript where items are reported
**Title and abstract**					
	1	(a) Indicate the study's design with a commonly used term in the title or the abstract (b) Provide in the abstract an informative and balanced summary of what was done and what was found	Page 1-2	RECORD 1.1: The type of data used should be specified in the title or abstract. When possible, the name of the databases used should be included.<br><br>RECORD 1.2: If applicable, the geographic region and timeframe within which the study took place should be reported in the title or abstract.<br><br>RECORD 1.3: If linkage between databases was conducted for the study, this should be clearly stated in the title or abstract.	Page 2
**Introduction**					
Background rationale	2	Explain the scientific background and rationale for the investigation being reported	Page 4-5		
Objectives	3	State specific objectives, including any prespecified hypotheses	Page 4-5		
**Methods**					
Study Design	4	Present key elements of study design early in the paper	Page 5-6		
Setting	5	Describe the setting, locations, and relevant dates, including periods of recruitment, exposure, follow-up, and data collection	Page 5-6		

For peer review only

Participants	6	*(a) Cohort study* - Give the eligibility criteria, and the sources and methods of selection of participants. Describe methods of follow-up<br>*Case-control study* - Give the eligibility criteria, and the sources and methods of case ascertainment and control selection. Give the rationale for the choice of cases and controls<br>*Cross-sectional study* - Give the eligibility criteria, and the sources and methods of selection of participants<br><br>*(b) Cohort study* - For matched studies, give matching criteria and number of exposed and unexposed<br>*Case-control study* - For matched studies, give matching criteria and the number of controls per case		RECORD 6.1: The methods of study population selection (such as codes or algorithms used to identify subjects) should be listed in detail. If this is not possible, an explanation should be provided.<br><br>RECORD 6.2: Any validation studies of the codes or algorithms used to select the population should be referenced. If validation was conducted for this study and not published elsewhere, detailed methods and results should be provided.<br><br>RECORD 6.3: If the study involved linkage of databases, consider use of a flow diagram or other graphical display to demonstrate the data linkage process, including the number of individuals with linked data at each stage.	Page 5
Variables	7	Clearly define all outcomes, exposures, predictors, potential confounders, and effect modifiers. Give diagnostic criteria, if applicable.	Page 6	RECORD 7.1: A complete list of codes and algorithms used to classify exposures, outcomes, confounders, and effect modifiers should be provided. If these cannot be reported, an explanation should be provided.	n/a
Data sources/ measurement	8	For each variable of interest, give sources of data and details of methods of assessment (measurement).<br>Describe comparability of assessment methods if there is more than one group	Page 5-6		

For peer review only

Bias	9	Describe any efforts to address potential sources of bias	Page 5		
Study size	10	Explain how the study size was arrived at	Page 5		
Quantitative variables	11	Explain how quantitative variables were handled in the analyses. If applicable, describe which groupings were chosen, and why	Page 6		
Statistical methods	12	(a) Describe all statistical methods, including those used to control for confounding<br>(b) Describe any methods used to examine subgroups and interactions<br>(c) Explain how missing data were addressed<br>(d) *Cohort study* - If applicable, explain how loss to follow-up was addressed<br>*Case-control study* - If applicable, explain how matching of cases and controls was addressed<br>*Cross-sectional study* - If applicable, describe analytical methods taking account of sampling strategy<br>(e) Describe any sensitivity analyses	Page 6		
Data access and cleaning methods		..		RECORD 12.1: Authors should describe the extent to which the investigators had access to the database population used to create the study population.	Page 5-6

For peer review only

				RECORD 12.2: Authors should provide information on the data cleaning methods used in the study.	
Linkage		..		RECORD 12.3: State whether the study included person-level, institutional-level, or other data linkage across two or more databases. The methods of linkage and methods of linkage quality evaluation should be provided.	Page 5
**Results**					
Participants	13	(a) Report the numbers of individuals at each stage of the study (*e.g.*, numbers potentially eligible, examined for eligibility, confirmed eligible, included in the study, completing follow-up, and analysed)<br>(b) Give reasons for non-participation at each stage.<br>(c) Consider use of a flow diagram	Page 6-7	RECORD 13.1: Describe in detail the selection of the persons included in the study (*i.e.*, study population selection) including filtering based on data quality, data availability and linkage. The selection of included persons can be described in the text and/or by means of the study flow diagram.	Page 5
Descriptive data	14	(a) Give characteristics of study participants (*e.g.*, demographic, clinical, social) and information on exposures and potential confounders<br>(b) Indicate the number of participants with missing data for each variable of interest<br>(c) *Cohort study* - summarise follow-up time (*e.g.*, average and total amount)	Page 6-7<br>Table 1-2		
Outcome data	15	*Cohort study* - Report numbers of outcome events or summary measures over time<br>*Case-control study* - Report numbers in each exposure	n/a		

For peer review only

		category, or summary measures of exposure<br>*Cross-sectional study* - Report numbers of outcome events or summary measures			
Main results	16	(a) Give unadjusted estimates and, if applicable, confounder-adjusted estimates and their precision (e.g., 95% confidence interval). Make clear which confounders were adjusted for and why they were included<br>(b) Report category boundaries when continuous variables were categorized<br>(c) If relevant, consider translating estimates of relative risk into absolute risk for a meaningful time period	n/a		
Other analyses	17	Report other analyses done—e.g., analyses of subgroups and interactions, and sensitivity analyses	Page 6-8		
**Discussion**					
Key results	18	Summarise key results with reference to study objectives	Page 8-10		
Limitations	19	Discuss limitations of the study, taking into account sources of potential bias or imprecision. Discuss both direction and magnitude of any potential bias	Page 10	RECORD 19.1: Discuss the implications of using data that were not created or collected to answer the specific research question(s). Include discussion of misclassification bias, unmeasured confounding, missing data, and changing eligibility over time, as they pertain to the study being reported.	n/a
Interpretation	20	Give a cautious overall interpretation of results considering objectives,	Page 11		

For peer review only

		limitations, multiplicity of analyses, results from similar studies, and other relevant evidence			
Generalisability	21	Discuss the generalisability (external validity) of the study results	Page 10		
**Other Information**					
Funding	22	Give the source of funding and the role of the funders for the present study and, if applicable, for the original study on which the present article is based	Page 6		
Accessibility of protocol, raw data, and programming code		..		RECORD 22.1: Authors should provide information on how to access any supplemental information such as the study protocol, raw data, or programming code.	Appendices Page 12

*Reference: Benchimol EI, Smeeth L, Guttmann A, Harron K, Moher D, Petersen I, Sørensen HT, von Elm E, Langan SM, the RECORD Working Committee. The REporting of studies Conducted using Observational Routinely-collected health Data (RECORD) Statement. *PLoS Medicine* 2015; in press.

*Checklist is protected under Creative Commons Attribution (CC BY) license.

For peer review only

BMJ Open

A prospective observational study of the challenges in diagnosing common neonatal conditions in Nigeria and Kenya

Journal:	*BMJ Open*
Manuscript ID	bmjopen-2022-064575.R1
Article Type:	Original research
Date Submitted by the Author:	16-Sep-2022
Complete List of Authors:	Staunton, Aimee; Liverpool School of Tropical Medicine,<br>Nabwera, Helen; Liverpool School of Tropical Medicine, International Public Health; Alder Hey Children's NHS Foundation Trust<br>Allen, Stephen ; Liverpool School of Tropical Medicine, Child Health; Alder Hey Children's NHS Foundation Trust<br>Tongo, Olukemi; University College Hospital Ibadan<br>Akindolire, Abimbola ; University College Hospital Ibadan<br>Abdulkadir, Isa; Ahmadu Bello University Teaching Hospital<br>Ezeaka, Chinyere; University of Lagos College of Medicine, Paediatrics<br>Ezenwa, Beatrice; University of Lagos College of Medicine, Paediatrics<br>Fajolu, Iretiola; College of Medicine University of Lagos, Department of Paediatrics; Lagos University Teaching Hospital<br>Imam, Zainab; Lagos State University Teaching Hospital<br>Umoru , Dominic ; Maitama District Hospital<br>Otieno, Walter; Maseno University; Jaramogi Oginga Odinga Teaching and Referral Hospital, Kisumu, Kenya<br>Nalwa, Grace; Maseno University, Department of Paediatrics and Child Health, ; Jaramogi Oginga Odinga Teaching and Referral Hospital, Kisumu, Kenya<br>Olwala, Macrine; Jaramogi Oginga Odinga Teaching and Referral Hospital, Kisumu, Kenya<br>Talbert, Alison; KEMRI-Wellcome Trust Research Programme<br>Andang'o, Pauline; Maseno University<br>Mwangome, MK; KEMRI-Wellcome Trust Research Programme,<br>Abubakar, Ismaela; Liverpool School of Tropical Medicine<br>Embleton, Nicholas; Newcastle University; Newcastle Upon Tyne Hospitals NHS Trust
**Primary Subject Heading**:	Public health
Secondary Subject Heading:	Diagnostics, Global health, Paediatrics, Public health
Keywords:	Health policy < HEALTH SERVICES ADMINISTRATION & MANAGEMENT, Public health < INFECTIOUS DISEASES, NEONATOLOGY, Paediatric gastroenterology < PAEDIATRICS

SCHOLARONE™
Manuscripts

I, the Submitting Author has the right to grant and does grant on behalf of all authors of the Work (as defined in the below author licence), an exclusive licence and/or a non-exclusive licence for contributions from authors who are: i) UK Crown employees; ii) where BMJ has agreed a CC-BY licence shall apply, and/or iii) in accordance with the terms applicable for US Federal Government officers or employees acting as part of their official duties; on a worldwide, perpetual, irrevocable, royalty-free basis to BMJ Publishing Group Ltd ("BMJ") its licensees and where the relevant Journal is co-owned by BMJ to the co-owners of the Journal, to publish the Work in this journal and any other BMJ products and to exploit all rights, as set out in our licence.

The Submitting Author accepts and understands that any supply made under these terms is made by BMJ to the Submitting Author unless you are acting as an employee on behalf of your employer or a postgraduate student of an affiliated institution which is paying any applicable article publishing charge ("APC") for Open Access articles. Where the Submitting Author wishes to make the Work available on an Open Access basis (and intends to pay the relevant APC), the terms of reuse of such Open Access shall be governed by a Creative Commons licence – details of these licences and which Creative Commons licence will apply to this Work are set out in our licence referred to above.

Other than as permitted in any relevant BMJ Author's Self Archiving Policies, I confirm this Work has not been accepted for publication elsewhere, is not being considered for publication elsewhere and does not duplicate material already published. I confirm all authors consent to publication of this Work and authorise the granting of this licence.

For peer review only

A prospective observational study of the challenges in diagnosing common neonatal conditions in Nigeria and Kenya

Aimee Staunton* [1], Helen M Nabwera[1,2], Stephen J Allen[1,2], Olukemi O Tongo[3], Abimbola E Akindolire[3], Isa Abdulkadir[4], V. Chinyere Ezeaka[5], Beatrice N Ezenwa[5], Iretiola B Fajolu[5,6], Zainab O Imam[6], Dominic D Umoru[7], Walter Otieno[8,9], Grace M Nalwa[8,9], Macrine Olwala[9], Alison W Talbert[10], Pauline EA Andang'o[8], Martha K Mwangome[10], Ismaela Abubakar[1], Nicholas D Embleton[11,12], on behalf of the Neonatal Nutrition Network (NeoNuNet)**

1. Liverpool School of Tropical Medicine, Pembroke place, Liverpool, L3 5QA, UK
2. Alder Hey Children's Hospital NHS Trust, E Prescot Rd, Liverpool L14 5AB, UK
3. University College Hospital, Queen Elizabeth Road, Ibadan, Nigeria
4. Ahmadu Bello University Teaching Hospital, Shika, Zaria, Nigeria
5. College of Medicine, University of Lagos, Idi-Araba, Lagos, Nigeria; Lagos University Teaching Hospital, Idi-Araba, Lagos, Nigeria.
6. Lagos State University Teaching Hospital, Ikeja, Lagos, Nigeria
7. Maitama District Hospital, 61 Aguiyi Ironsi St, Maitama, Abuja, Nigeria
8. Maseno University, Maseno, Kenya
9. Jaramogi Oginga Odinga Teaching and Referral Hospital, Jomo Kenyatta Highway Kaloleni Kisumu KE, Central, Kenya
10. KEMRI-Wellcome Trust Research Programme, PO Box 230-80108, Kilifi, Kenya
11. Newcastle University, Newcastle upon Tyne NE1 7RU, UK
12. The Newcastle upon Tyne Hospitals NHS Foundation Trust, High Heaton, NE7 7DN, UK

**Corresponding author, contact: aimee.staunton@ntlworld.com*

***Full Neonatal Nutrition Network members list in Appendix 1*

Short title: A prospective observational study of the challenges in diagnosing common neonatal conditions in Nigeria and Kenya

Number of tables: *2*

Number of figures: *1*

For peer review only - http://bmjopen.bmj.com/site/about/guidelines.xhtml

For peer review only

Abstract

Objectives: Accurate and timely diagnosis of common neonatal conditions is crucial for reducing neonatal deaths. In low- and middle-income countries with limited resources, there is sparse information on how neonatal diagnoses are made. The aim of this study was to describe the diagnostic criteria used for common conditions in neonatal units (NNUs) in Nigeria and Kenya.

Design: Prospective observational study. Standard case report forms for suspected sepsis, respiratory disorders, birth asphyxia and abdominal conditions were co-developed by the Neonatal Nutrition Network* collaborators. Clinicians completed forms for all admissions to their NNU. Key data was displayed using heatmaps.

Setting: Five NNUs in Nigeria and two in Kenya comprising the Neonatal Nutrition Network.

Participants: 2851 neonates, which included all neonates admitted to the seven NNUs over a six month period.

Results: 1230 (43.1%) neonates had suspected sepsis, 874 (30.6%) respiratory conditions, 587 (20.6%) birth asphyxia and 71 (2.5%) abdominal conditions. For all conditions and across all NNUs, clinical criteria were used consistently with sparse use of laboratory and radiological criteria.

Conclusion: Our findings highlight the reliance on clinical criteria and extremely limited use of diagnostic technologies for common conditions in NNUs in sub-Saharan Africa. This has implications for the management of neonatal conditions which often have overlapping clinical features. Strategies for implementation of diagnostic pathways and investment in affordable and sustainable diagnostics are needed to improve care for these vulnerable infants.

* https://www.lstmed.ac.uk/nnu

For peer review only - http://bmjopen.bmj.com/site/about/guidelines.xhtml

Strengths and limitations

Strengths

- The use of heatmaps to display diagnostic pathways highlights key patterns in the diagnostic criteria used by clinicians.
- Meticulous data collection, designed and supervised by local clinicians and international researchers, has provided fresh insights into diagnostic practice in this under researched population.

Limitations

- Most network NNUs were urban, tertiary level facilities serving families that could likely better afford investigations so that our findings may not be generalisable to NNUs serving poorer populations.

For peer review only

For peer review only - http://bmjopen.bmj.com/site/about/guidelines.xhtml

Background

The majority of the 2.4 million global neonatal deaths (< 28 days) in 2019 were due to three largely preventable or treatable conditions: complications of preterm birth, intrapartum-related events and sepsis (1). Accurate and timely diagnosis of these common conditions is crucial for reducing mortality (1, 2). The clinical assessments and decisions from presentation to diagnosis, referred to as diagnostic pathways, are known to influence outcomes (3). Although access to diagnostics is included in one of the six WHO health system building blocks, there are limited data on diagnostic pathways and how these influence outcomes in neonatal care in low-and middle-income countries (LMICs) (4-6).

In Nigeria and Kenya, concerted actions are required to reduce neonatal mortality (7). Progress in reducing neonatal mortality rates (NMR; the number of deaths occurring 0-<28 days per 1000 live births) has been slow in both countries (8). Between 1990 and 2019, NMR in Nigeria decreased by 28% from 50 to 36 per 1000 and in Kenya by 25% from 28 to 21 per 1000 (1). Consequently, both countries are unlikely to meet the sustainable development goal (SDG 3.2) for neonatal mortality and are thus classified as priority countries in the Countdown to 2030 initiative (9).

In the context of robust health systems, national or regional guidelines may support the standardisation and accuracy of diagnoses as a means of improving care and reducing mortality in highly vulnerable hospitalised infants (10, 11). Whilst both countries have policies on newborn health, only Kenya had national guidelines for the management of common neonatal conditions at the time of the study (12, 13). However, there is limited information on the impact of guidelines in low-resource settings, where challenges with resources for health are greatest (14).

Encouraged by international efforts to improve access to diagnostics and the sparse information on how common neonatal diagnoses are made (5, 15-17), we sought to describe

For peer review only - http://bmjopen.bmj.com/site/about/guidelines.xhtml

the diagnostic criteria used by clinicians in seven neonatal units (NNUs) in Nigeria and Kenya participating in the Neonatal Nutrition Network (NeoNuNet https://www.lstmed.ac.uk/nnu). Network members can be found in appendix 1.

Methods

Design and setting

In this prospective observational study, we collected information from five NNUs in Nigeria and two in Kenya; five providing tertiary level care, two providing district level care and all but one situated in major cities (18). All NNUs provided supportive feeding, intravenous fluids, antibiotics, phototherapy and bubble continuous positive airway pressure (CPAP). Two out of seven NNUs provide free neonatal healthcare services. Further details of the NNUs are available in appendix 2.

Data Collection

Case report forms were developed for birth asphyxia, respiratory disorders, abdominal conditions, and suspected sepsis with reference to international and local guidelines. Respiratory conditions included respiratory distress syndrome, transient tachypnoea of the newborn, pneumonia and meconium aspiration. Abdominal conditions included necrotizing enterocolitis (NEC), dysmotility, septic ileus and focal intestinal perforation. Suspected sepsis was defined as clinical assessment resulting in starting or changing antibiotic treatment. Over a 6-month period in each NNU between September 2018 and April 2019, clinicians completed case report forms for all admissions and each episode of illness. A new episode occurred if the infant has been symptom free for 48 hours or more after any previous episode. Only the first episode of each illness was included in this analysis. Demographic and clinical information was extracted from the main NeoNuNet database. For out-born infants in whom birthweight was not known, weight on admission was used instead of birthweight. Completed case report forms were reviewed by a senior clinician. Data clerks entered anonymised data into a

For peer review only - http://bmjopen.bmj.com/site/about/guidelines.xhtml

password protected REDCap database hosted by the Liverpool School of Tropical Medicine (LSTM) (10, 19). Ethical approval was granted (appendix 3).

Patient and Public Involvement

Study participants and the public were not involved in the design or undertaking of the study.

Data management and analysis

Data was imported to SPSS version 25 (20) for analysis. Categorical variables were presented as frequencies and percentages. Diagnostic criteria were presented using heatmaps (Microsoft Office 16 Excel). Each criterion was classified as either used (with either a positive or negative finding) or not used. Criteria were grouped into clinical, laboratory, clinical/laboratory, and radiological criteria. The 'COUNTIFS' function was used to calculate the proportion of cases in which the criterion was used, and a divergent colour scheme was applied to the heatmap cells.

Results

Over the 6 months' data collection period, 2851 neonates were admitted to the seven NNUs. The majority of newborns were male (1626; 57.1%), most had been delivered in a health facility (2600; 91.2%) and just over half were vaginal unassisted deliveries (1586; 55.7%). Overall, 1261 (45.3%) were preterm (<37 weeks' gestation), 1405 (49.3%) were low birth weight (<2.50kg) and 473 (16.6%) infants died (Table 1). The proportions of very preterm (VPT, <32 weeks' gestation) or very low birth weight (VLBW, <1500g) infants varied considerably across the NNUs (Table 2).

For peer review only - http://bmjopen.bmj.com/site/about/guidelines.xhtml

Table 1: Newborn characteristics of 2851 admissions[1]

**Variable**	
Male gender[2]	1626 (57.1)
Place of delivery	
• Health facility	2600 (91.2)
• Home	165 (5.8)
• Other	86 (3.0)
Mode of delivery[3]	
• Vaginal unassisted	1586 (55.7)
• Caesarean section	1160 (40.7)
• Vaginal assisted/ instrumental	101 (3.5)
Gestational age in weeks[4] Median (IQR)	37.0 (33.0-39.0)
Gestational age category[4]	
• Extreme preterm (<28 weeks)	122 (4.3)
• Very preterm (28-<32 weeks)	378 (13.3)
• Moderately preterm (32-<37 weeks)	761 (26.7)
Birth weight or weight on admission in kg[5] Median (IQR)	2.50 (1.67-31.7)
Birth weight or weight on admission category[5] (n; %)	
• ELBW (<1000g)	116 (4.1)
• VLBW (1000-1499g)	393 (13.8)
• LBW (1500-2499g)	896 (31.5)
Antibiotics given after birth[6]	1294 (45.4)
Final infant outcome[7]	
• Discharged with no morbidities	2170 (76.1)
• Discharged with morbidities	89 (3.1)
• Transferred out	43 (1.5)
• Absconded/ discharged against medical advice	55 (1.9)
• Died	473 (16.6)

Notes

1. Variables are shown as number (%) unless otherwise stated
2. Gender missing in 2
3. Mode of delivery missing in 4

For peer review only

For peer review only - http://bmjopen.bmj.com/site/about/guidelines.xhtml

4. Gestational age missing for 68
5. Birthweight and weight on admission missing in 3
6. Prophylactic antibiotics missing in 36
7. Final infant outcome missing in 21

For peer review only

For peer review only - http://bmjopen.bmj.com/site/about/guidelines.xhtml

Table 2: Admission characteristics and frequency of common conditions according to neonatal unit[1,2]

Condition	Kenya		Nigeria					Total
	1	2	3	4	5	6	7	
Total admissions	291	613	181	732	384	238	412	2851
Extreme or very preterm (<32 weeks' gestation)	52 (17.9)	94 (16.3)	9 (5.1)	124 (17.4)	142 (37.1)	69 (29.1)	10 (2.5)	500 (27.8)[3]
Extreme or very low birth/admission weight (<1500g)	60 (20.6)	107 (17.5)	13 (3.3)	104 (14.2)	128 (33.3)	69 (29.0)	28 (6.8)	509 (28.5)[4]
Suspected sepsis	132 (45.4)	421 (68.7)	68 (37.6)	405 (53.3)	49 (12.8)	46 (19.3)	109 (26.5)	1230 (43.1)
Respiratory conditions	78 (26.8)	364 (59.4)	19 (10.5)	210 (28.7)	18 (4.7)	162 (68.1)	23 (5.6)	874 (30.7)
Birth asphyxia	48 (16.5)	189 (30.8)	20 (11.0)	190 (26.0)	41 (10.7)	44 (18.5)	55 (13.3)	587 (20.6)
Abdominal conditions	3 (1.0)	4 (0.7)	6 (3.3)	32 (4.4)	3 (0.8)	14 (5.9)	9 (2.2)	71 (2.5)

Notes:

1. Data are shown as number (%)
2. More than one condition was recorded in many infants
3. Gestation was missing for 35 infants in unit 2, 5 in unit 3, 20 in unit 4, 1 each in units 5 and 6 and 6 in unit 7
4. Birth/admission weight was missing for 2 infants in unit 2 and 1 infant in unit 7

For peer review only

Diagnostic criteria

Overall, forms were filled in for 1230 (43.1%) newborns diagnosed with suspected sepsis, 874 (30.6%) with respiratory conditions, 587 (20.6%) with birth asphyxia and 71 (2.5%) with abdominal conditions (Table 2). The frequency of these diagnoses varied considerably between NNUs including those with similar proportions of VPT or VLBW infants. Overall, across all NNUs, diagnoses were based mainly on clinical criteria and a full blood count with limited use of other laboratory assays or radiological investigations (*Fig 1a-1d*).

For the diagnosis of suspected neonatal sepsis, clinical criteria were used uniformly in all NNUs (*Fig 1a*). The exceptions were hypotension where use varied from 0% to 98% (Appendix 4a). Assessment of glucose intolerance, often using a point of care test, and parameters from a full blood count were the most used laboratory criteria. However, other laboratory parameters requiring biochemical analyses and microbiology were used much less frequently overall.

Similarly, for respiratory conditions, clinical criteria were used uniformly across NNUs (*Fig 1b; appendix 4b*). In marked contrast to the limited use of laboratory analyses, the use of chest Xray appeared to be good overall but with some variability between NNUs.

The diagnosis of birth asphyxia was based on clinical evidence of encephalopathy and exclusion of other aetiologies. Evidence of multiorgan dysfunction was also used although this may have been based on the clinical components (acute kidney injury, respiratory distress, circulatory collapse and disseminated intravascular coagulation) rather than laboratory analyses. In contrast, use of cranial ultrasound scan varied widely ranging from 0-84% (Fig 1c; appendix 4c).

For peer review only - http://bmjopen.bmj.com/site/about/guidelines.xhtml

For the infants with an abdominal condition, of which about half were diagnosed as NEC (36/71, 50.7%), a range of clinical criteria were used in nearly all cases in most NNUs. Basic laboratory criteria derived from a full blood count were also frequently used but use of parameters of metabolic acidosis was more variable. Overall, radiology criteria were used less frequently (*Fig 1d, appendix 4d).*

Discussion

This study enabled us to explore diagnostic pathways and identify key challenges in the diagnosis of common neonatal conditions in the context of limited resources. Clinicians were generally constrained to a symptoms-based, clinical orientated diagnostic approach. This is consistent with the widely available WHO Pocket Book which encourages a syndromic approach and is a common source of diagnostic guidelines (21). However, the Pocket Book is targeted at first level referral hospitals, whereas greater access to diagnostics is required for higher-level care. Furthermore, for some neonatal conditions such as respiratory distress syndrome, a clinical diagnosis may be appropriate; however, given the overlapping features between neonatal conditions and the potential lack of adequate clinical history for out-born patients in particular, laboratory and radiological investigations are important to support accurate diagnosis.

The consistent use of a wide range of clinical criteria across the NNUs was a positive finding suggesting uniformity in clinical practice. This is likely supported by use of national neonatal clinical guidelines although these were available in Kenya only. National paediatric diagnostic guidelines have been found to optimise practice in high-income settings; however, this may be more challenging in LMICs where diagnostic resources and skilled workforce remain inadequate making consistent adherence to clinical guidelines difficult (22, 23). This is further complicated by political instability, healthcare strikes and high staff turnover due to poor

For peer review only - http://bmjopen.bmj.com/site/about/guidelines.xhtml

working conditions which may result in gaps in training on appropriate use of diagnostic technologies when they are available (24, 25).

In marked contrast with clinical criteria, the limited use of laboratory investigations beyond a full blood count, shows that there were challenges implementing standardised diagnostic pathways. There was obvious overlap between conditions, so that investigations such as blood culture were used infrequently in both suspected sepsis and respiratory illness. Although some laboratory procedures were available in individual hospitals, in our experience, this is often short-term due to dependency on donor or research support. The limited use of laboratory investigations within the NNUs may be due to a lack of affordable and quality assured laboratory and specimen transport services, which are common barriers in LMICs (5, 26). This is likely to have implications on the accuracy of diagnoses, since a syndromic diagnostic approach has been linked to lower specificity, thereby resulting in more false positive cases (27). The lack of laboratory diagnostic support in our study would likely result in the overdiagnosis of neonatal sepsis. A direct consequence is the overuse of antibiotics in this population, which could drive antimicrobial resistance (28). Use of laboratory investigations to complement clinical assessment, as well as antibiotic stewardship programmes, would likely help (29).

For peer review only

Respiratory conditions such as respiratory distress syndrome were typically diagnosed using clinical criteria but also supported by chest X rays which are critically important in distinguishing the cause of respiratory distress, which includes a number of pathologies with overlapping clinical signs (30). The diagnosis of birth asphyxia was also based mainly on clinical criteria and with limited use of cranial ultrasound. In addition to diagnosis, laboratory support with determining acid-base balance and blood electrolytes is required in the selection

For peer review only - http://bmjopen.bmj.com/site/about/guidelines.xhtml

of infants for treatment approaches such as therapeutic hypothermia and monitoring treatment response (31).

In contrast to the use of X ray in respiratory conditions, abdominal Xray, a key investigation for the diagnosis of conditions such as NEC, was only used consistently in one unit. The limited use may be due to more severe illness in infants with suspected NEC and the lack of mobile X ray machines. Previous research in LMIC settings has reported that diagnosis is impaired by a lack of abdominal X rays, since even when X ray machines are available and functioning, they are often too expensive for patients and families to afford (32). This is likely here since only two out of the seven NNUs provide free of charge neonatal health services. This means that diagnosis is very difficult for conditions such as NEC where specific X ray findings are critically important (33).

Given the consistent use of clinical criteria across the NNUs, the limited use of laboratory and radiological investigations likely contributed to the marked differences between units in the frequencies of diagnoses. This would appear to be less likely due to differences in case mix, since variability in the frequency of diagnoses occurred between NNUs with similar proportions of small and preterm newborns in whom these conditions are common. These challenges highlight the need for sustainable and affordable diagnostic tests suitable for use in resource limited settings for accurate and timely diagnosis and to reliably assess the frequency of the main causes of neonatal mortality. This should include point-of-care diagnostic tests designed for use in LMICs which can provide rapid results and reduce the workload for laboratory services (34, 35).

Conclusions

Our findings demonstrate that diagnostic pathways for common conditions in NNUs in Nigeria and Kenya were hindered by the limited use of laboratory and radiological technologies.

For peer review only - http://bmjopen.bmj.com/site/about/guidelines.xhtml

Improving capacity to diagnose common conditions rapidly and reliably in hospitalised newborns should be a key priority of future healthcare quality improvement initiatives. The development of national plans to increase the availability, accessibility, and affordability of point of care and other diagnostic investigations is paramount since the need for out-of-pocket health spending hinders compliance to clinical guidelines (5). This will be dependent on infrastructure strengthening and appropriate staff training and education. Our findings will inform the development and implementation of integrated context-relevant best practice guidelines for neonatal care in low- resource settings. Further research is necessary to examine the impact of such guidelines on neonatal health in these settings.

Acknowledgements

We would like to thank our colleagues who contributed to the clinical care and collection of data at the Network neonatal units. We would also like to thank colleagues at the Nigerian Society of Neonatal Medicine, the Kenya Paediatric Association and the Ministries of Health in Nigeria and Kenya, who provided us with support and advice as we were setting up this study. We thank the mothers and families immensely for participating in this study with their infants. This study is published with the permission of the Director of Kenya Medical Research Institute.

Contributorship statement

Stephen J Allen conceived the study. Aimee Staunton, Stephen J Allen, Helen M Nabwera, Olukemi O Tongo, Abimbola E Akindolire, Isa Abdulkadir, V. Chinyere Ezeaka, Beatrice N Ezenwa, Iretiola B Fajolu, Zainab O Imam, Dominic D Umoru, Walter Otieno, Grace M Nalwa, Macrine Olwala, Alison W Talbert, Pauline EA Andandg'o, Martha K Mwangome, Ismaela Abubakar and Nicholas D Embleton designed the study and each read, helped to critically revise and approved the final manuscript version and agree to be accountable for the accuracy and integrity of the manuscript and to answer any questions in relation to the work. Aimee

For peer review only - http://bmjopen.bmj.com/site/about/guidelines.xhtml

Staunton analysed the data. Aimee Staunton, Stephen J Allen and Helen M Nabwera prepared the manuscript. Aimee Staunton is the corresponding author responsible for submission, peer review and publication processes.

Open access

This is an open access article distributed in accordance with the Creative Commons Attribution 4.0 Unported (CCBY4.0) license, which permits others to copy, redistribute, remix, transform and build upon this work for any purpose, provided the original work is properly cited, a link to the licence is given, and indication of whether changes were made.

Data sharing

Further data from the heatmaps is available in appendix 4. Requests for access to further data should be addressed to the corresponding author.

Ethics approval

Ethical approval was granted by the Research Ethics Committees at LSTM (18–0210), Lagos University Teaching Hospital Health REC (AMD/DCST/HREC/APP/2514), the Jaramogi Oginga Odinga Teaching and Referral Hospital (ERC.IB/VOL.1/510), University College Hospital Ibadan (UI/EC/18/0446), Massey Street Children's Hospital (LSHSC/2222/VOL.VI[B]/185), Ahmadu Bello University Teaching Hospital (ABUTH/HZ/HREC/D37/2018), Maitama District Hospital (FHREC/2018/01/108/19-09-18), the Kenya Medical Research Institute-Scientific and Ethics Review Unit (KEMRI/SERU/CGMR-C/120/3740) (10) (Appendices 3a-3b).

For peer review only - http://bmjopen.bmj.com/site/about/guidelines.xhtml

Funding

This project was funded by a grant from the MRC Confidence in Global Nutrition and Health Research scheme (grant reference MC_PC_MR/R019789/1); the funders had no role in the design or conduction of the study.

Conflict of Interests Statement

None to declare.

For peer review only

For peer review only - http://bmjopen.bmj.com/site/about/guidelines.xhtml

References

1. United Nations Inter-agency Group for Child Mortality Estimation (UN IGME). Levels and Trend in Child Mortality Report. 2020.
2. Healthy Newborn Network. Explore Countries- Nigeria. healthynewbornnetwork.org.2017 [Available from: https://www.healthynewbornnetwork.org/country/nigeria/.
3. National Academies of Sciences Engineering and Medicine. Improving diagnosis in health care: National Academies Press; 2015.
4. World Health Organization. Everybody's business-strengthening health systems to improve health outcomes: WHO's framework for action. 2007.
5. Fleming K, Horton S, Wilson L, et al. The Lancet Commission on diagnostics: transforming access to diagnostics. *The Lancet*. 2021;398:1997-2050.
6. Pius S, Bello M. Neonatal Septicaemia in Poor Resource Settings. *Pediatric Infect Dis*. 2017;2:2537-0282.
7. The World Bank. World Bank Country and Lending Groups. worldbank.org.2018 [Available from: https://datahelpdesk.worldbank.org/knowledgebase/articles/906519.
8. World Health Organization. Progress Report: Reaching every newborn national 2020 milestones: country progress, plans and moving forward. 2018.
9. Boerma T, Requejo J, Victora C, et al. Countdown to 2030: tracking progress towards universal coverage for reproductive, maternal, newborn, and child health. *The Lancet*. 2018;391:1538-1548.
10. Nabwera HM, Wang D, Tongo OO, et al. Burden of disease and risk factors for mortality amongst hospitalized newborns in Nigeria and Kenya. *PloS one*. 2021;16: e0244109.
11. Bhutta ZA, Giuliani F, Haroon A, et al. Standardisation of neonatal clinical practice. *BJOG*. 2013;120:56-63.
12. Federal Ministry of Health. Nigeria Every Newborn Action Plan: A plan to end preventable newborn deaths in Nigeria. 2016.
13. Ministry of Health. Health ministry launches policy to accelerate gains in child health. 2018 [Available from: http://www.health.go.ke/health-ministry-launches-policytoaccelerate-gains-in-child-health-nairobi-kenya-november-19-2018/.
14. Ballot DE, Chirwa TF, Cooper PA. Determinants of survival in very low birth weight neonates in a public sector hospital in Johannesburg. *BMC Pediatr.* 2010;10:30.

For peer review only - http://bmjopen.bmj.com/site/about/guidelines.xhtml

15. Engel N, Wachter K, Pai M, et al. Addressing the challenges of diagnostics demand and supply: insights from an online global health discussion platform. *BMJ Glob Health*. 2016;1:e000132.
16. Welcome MO. The Nigerian health care system: Need for integrating adequate medical intelligence and surveillance systems. *J Pharm Bioallied Sci*. 2011;3:478.
17. English M, Gathara D, Nzinga J, et al. Lessons from a Health Policy and Systems Research programme exploring the quality and coverage of newborn care in Kenya. *BMJ Glob Health*. 2020;5:e001937.
18. Liverpool School of Tropical Medicine. About the Neonatal Nutrition Network [Available from: https://www.lstmed.ac.uk/research/departments/clinicalsciences/neonatal-nutritionnetwork/about-the-neonatal-nutrition.
19. Harris PA, Taylor R, Minor BL, et al. The REDCap consortium: Building an international community of software platform partners. *J Biomed Inform.* 2019;95:103208.
20. IMB Corp. IBM SPSS statistics for Windows, version 20.0. 2011.
21. World Health Organization. Pocket book of hospital care for children: guidelines for the management of common childhood illnesses. 2013.
22. Akenroye AT, Baskin MN, Samnaliev M, et al. Impact of a bronchiolitis guideline on ED resource use and cost: a segmented time-series analysis. *Pediatrics*. 2014;133:227-234.
23. Lopez AA, Aslanova R, Bridger N, et al. Antibiotic Use for Inpatient Bronchiolitis: Did National Guidelines Impact Practice at a Pediatric Hospital? *Hospital Pediatrics*. 2020;10:147-152.
24. Irimu G, Ogero M, Mbevi G, et al. Tackling health professionals' strikes: an essential part of health system strengthening in Kenya. *BMJ Glob Health*. 2018;3:e001136.
25. UNICEF. Country Office Annual Report- Kenya. unicef.org; 2018.
26. Majors CE, Smith CA, Natoli ME. Point-of-care diagnostics to improve maternal and neonatal health in low-resource settings. *Lab on a Chip*. 2017;17:3351-3387.
27. Mabey D, Vos T. Syndromic approaches to disease management. *The Lancet*. 1997;349:26-28.
28. Ellington LE, Gilman RH, Chavez MA, et al. Lung ultrasound as a diagnostic tool for radiographically-confirmed pneumonia in low resource settings. *Respiratory Medicine*. 2017;128:57-64.
29. Camacho-Gonzalez A, Spearman PW, Stoll BJ. Neonatal infectious diseases:

For peer review only

evaluation of neonatal sepsis. *Pediatric Clinics*. 2013;60:367-389.

30. Gallacher DJ, Hart K, Kotecha S. Common respiratory conditions of the newborn. *Eur Respir* J. 2016;12:30-42.
31. Lemyre B, Chau V. Hypothermia for newborns with hypoxic-ischemic encephalopathy. *Paediatrics & child health*. 2018;4:285-295.
32. Filkins M, Halliday S, Daniels B, et al. Implementing Diagnostic Imaging Services in a Rural Setting of Extreme Poverty: Five Years of X-ray and Ultrasound Service Delivery in Achham, Nepal. *Journal of Global Radiology*. 2015;1:1-2.
33. Gephart SM, Gordon PV, Penn AH, et al. Changing the paradigm of defining, detecting, and diagnosing NEC: Perspectives on Bell's stages and biomarkers for NEC. *Semin Pediatr Surg*. 2018;27:3-10.
34. Schito M, Peter TF, Cavanaugh S, et al. Opportunities and Challenges for CostEfficient Implementation of New Point-of-Care Diagnostics for HIV and Tuberculosis. *J Infect Dis*. 2012;205:169-180.
35. Drain PK, Hyle EP, Noubary F, et al. Diagnostic point-of-care tests in resourcelimited settings. *Lancet Infect Dis*. 2014;14:239-249.

For peer review only

For peer review only - http://bmjopen.bmj.com/site/about/guidelines.xhtml

Figure 1: Heatmaps showing the diagnostic criteria used for common neonatal conditions

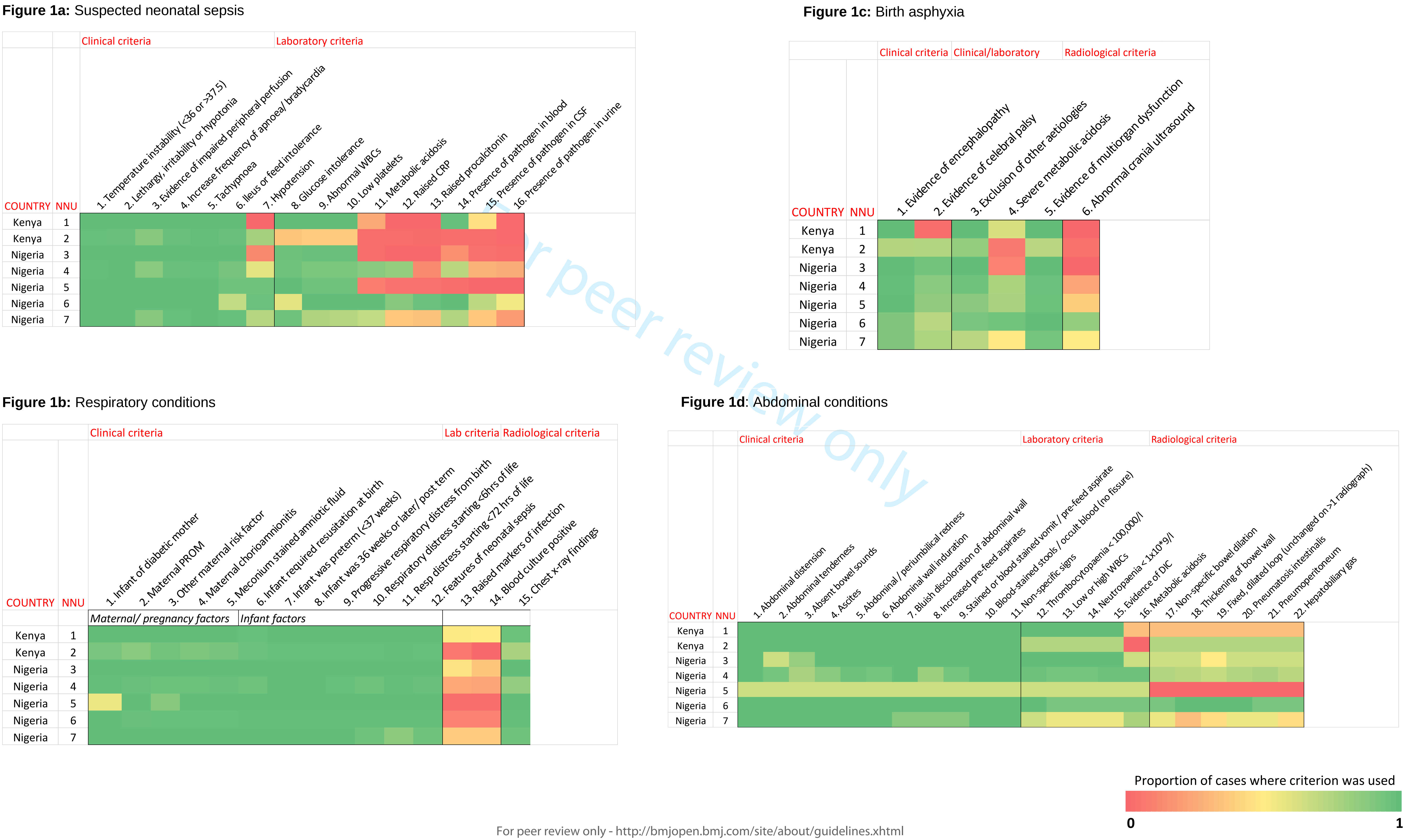

For peer review only

Appendix 1*: Neonatal Nutrition Network Members*

Isa Abdulkadir (Ahmadu Bello University, Nigeria); Ismaela Abubakar (LSTM, UK); Abimbola E Akindolire (College of Medicine, University of Ibadan, Nigeria); Olusegun Akinyinka (College of Medicine, University of Ibadan, Nigeria); Stephen J Allen (LSTM, UK); Pauline EA Andang'o (Maseno University, Kenya); Graham Devereux (LSTM, UK); Chinyere Ezeaka (Lagos University Teaching Hospital, Nigeria); Beatrice N Ezenwa (Lagos University Teaching Hospital, Nigeria); Iretiola B Fajolu (Lagos University Teaching Hospital, Nigeria); Zainab O Imam (Lagos State University Teaching Hospital, Nigeria); Kevin Mortimer (LSTM, UK); Martha K Mwangome (KEMRI Wellcome Trust Research Programme, Kenya); Helen M Nabwera (LSTM, UK); Grace M Nalwa (Jaramogi Oginga Odinga Teaching and Referral Hospital, Kenya & Maseno University, Kenya); Walter Otieno (Jaramogi Oginga Odinga Teaching and Referral Hospital, Kenya & Maseno University, Kenya); Alison W Talbert (KEMRI Wellcome Trust Research Programme, Kenya); Nicholas D Embleton (Newcastle University, UK); Olukemi O Tongo (College of Medicine, University of Ibadan, Nigeria); Dominic D Umoru (Maitama District Hospital, Nigeria); Janneke van de Wijgert (University of Liverpool, UK); Melissa Gladstone (University of Liverpool, UK).

For peer review only

Appendix 2: Neonatal unit details

Location	Rural or urban	Secondary of tertiary level	No. of neonatal beds	Staffing
The Neonatal Unit, Lagos University Teaching Hospital (luth), Lagos, Nigeria	Urban	Tertiary	80 beds	- 4 consultant neonatologists<br>- 10 resident doctors<br>- 26 nurses<br>- 8 support staff
The Neonatal Unit of Ahmadu, Bello University Teaching Hospital Shika, Zaria, Kaduna, Nigeria	Urban	Tertiary	24 beds	- 3 neonatologists<br>- 5 senior registrars<br>- 4 registrars<br>- 18 nurses<br>- Support staff
Neonatal Unit, Maitama District Hospital, Abuja, Nigeria	Urban	Secondary	8 beds	- 3 neonatologists<br>- 2 medical Officers<br>- 2-3 nurses<br>- Support staff
Massey Street Children's Hospital, Lagos Island, Nigeria	Urban	Tertiary	56 beds	- 2 neonatologists<br>- 1 senior registrar<br>- 4 registrars<br>- 31 nursing staff
University College Hospital, Ibadan, Nigeria	Urban	Tertiary	38 beds	- 3 neonatologists<br>- 4 senior registrars<br>- 4 registrars<br>- House officers<br>- 50 nurses
Kilifi County Hospital, Kilifi Town, Kenya	Urban	Secondary	18 beds	- 2 paediatric consultants,<br>- 6 clinical officers<br>- 3-4 neonatal nurses
Jaramogi Oginga Odinga Teaching and Referral Hospital (JOOTRH), Kisumu City, Kenya	Urban	Tertiary	21 beds	- 2 paediatric consultants,<br>- 2 medical officers<br>- 1-2 medical officers<br>- 1-2 neonatal nurses

For peer review only

Appendix 3a: Ethical approval details from collaborating institutions

Institution	Protocol number
**Liverpool School of Tropical Medicine Research Ethics Committee (REC)**	18-0210
**The Lagos University Teaching Hospital Health Research Ethics Committee**	AMD/DCST/HREC/APP/2514
**The research and ethics committees at The Jaramogi Oginga Odinga Teaching and Referral Hospital**	ERC.IB/VOL.1/510
**University College Hospital Ibadan**	UI/EC/18/0446
**Massey Street Children's Hospital**	LSHSC/2222/VOL.VI^{B}/185
**Ahmadu Bello University Teaching Hospital**	ABUTH/HZ/HREC/D37/2018
**Maitama District Hospital**	FHREC/2018/01/108/19-09-18
**The Kenya Medical Research Institute-Scientific and Ethics Review Unit**	KEMRI/SERU/CGMR-C/120/3740

For peer review only

Appendix 3b: Confirmation of data access request from KEMRI- Wellcome Trust

**1. REQUESTOR DETAILS**					
**Name**	Aimee Staunton				
**Contact details: address, phone and email**	97 Aigburth Road Liverpool L17 4JU Liverpool School of Tropical Medicine	**Phone**	07530351521	**Email**	264266@lstmed.ac.uk

**2. STUDY DETAILS**			
**Title of Research Project**	Describing the diagnostic pathways for common neonatal conditions across seven neonatal units in sub-Saharan Africa	**Start & end dates**	June 2020-September 2020
**Ethical approval sought / planned (with dates)**	Ethics application covered by existing approved proposal:<br><br>Title of proposal: Improving the survival, growth and development of low birth weight newborns through better nutrition<br>LSTM Research Ethics Committee, reference number: 18-0210<br>PI: Prof Stephen Allen<br>Date of approval: 4th July 2018<br>End date for data collection: 31st August 2019		

3. INVESTIGATORS

Names of all investigators who will have access to data (begin with Principal Investigator)* include names of KWTRP collaborators where applicable.

**Name**	**Role**	**Institution**
Aimee Staunton	Principal Investigator (MSc student)	LSTM
Helen Nabwera	Project Supervisor	LSTM
Stephen Allen	Project Supervisor	LSTM

4. BRIEF DESCRIPTION TYPE OF DATA REQUIRED

The dataset collected by NeoNuNet to record diagnostic pathways, comprising all admissions to the seven network neonatal units over a six-month period between August 2018 and May 2019. This includes 2280 neonates in total.

Title: Improving the survival, growth and development of low birth weight newborns through better nutrition

PI: Dr Martha Mwangome

KEMRI/SERU/CGMT-C/120/3740

5. OBJECTIVES AND STUDY DETAILS INCLUDING TIME FRAME

1. To describe the population of neonates and the causes of admission to secondary and tertiary level neonatal units in sub-Saharan Africa using secondary data collected from seven neonatal units in Nigeria and Kenya by September 2020.
2. To describe how common neonatal conditions (asphyxia, respiratory disorders, abdominal conditions/ necrotizing enterocolitis and neonatal sepsis) are diagnosed in the seven neonatal units in Nigeria and Kenya by September 2020.
3. To identify any gaps or inaccuracy in the diagnostic pathways for common neonatal conditions in the seven neonatal units and explore the association with neonatal outcomes by September 2020.

6. BRIEF DESCRIPTION OF ANALYSIS PLANNED WITHIN KIDMS DATA ***specify if study specific data not KIDMS data**

Objective 1: To fulfil this objective, data will be analysed using SPSS to describe the population of neonates. The relative percentages of the different diagnoses will be calculated. Descriptive statistics for variables such as gestation, sex and birthweight will be generated and basic hypothesis testing will be carried out. This will help to describe the biodemographic characteristics of the population being studied.

Objective 2: To fulfil this objective, the diagnostic pathways that lead to the final diagnosis (birth asphyxia, respiratory disorders, abdominal conditions/ necrotizing enterocolitis (NEC) or neonatal sepsis) will be analysed. This will be

Conditions and limitations for data sharing

You understand and acknowledge that custodianship of the Data is held jointly by the Programme and the Ministry of Health in Kilifi, and will be retained by the Programme and the Ministry of Health in Kilifi in case of any data transfer to a Recipient.
You understand and acknowledge that the Data is experimental in nature, and that access to the Data is provided without any representations or warranties of any kind in relation to the Database or the Data.
You will only access the Database and use the Data for the Agreed Purpose and shall not use the Data in such a way that damage or distress is or is reasonably likely to be caused to any Data Subjects.
The Data relates directly to individual Data Subjects and is strictly confidential. Data from subjects will be shared in a fully anonymised form without access to personal identifiers, including names and/or specialised roles. Geographic identifiers for subjects will routinely be aggregated to the level of the sub location or similarly de-identified. Consideration of requests for data with greater granularity will be informed by the local importance of the research question and the ability to protect the confidentiality of individuals and communities.
You shall only disclose the Data to your Data Users. You shall take all reasonable measures to ensure that your Data Users shall not make copies of the whole or any part of the Data without your consent, and shall keep a written record of any such copies sufficient to permit you to fulfil your obligations under clauses 3 and 7 of this Agreement. You shall not transfer or disclose any part of the Data to any other person or body.
You understand and acknowledge that the Data is protected by copyright and other intellectual property rights.
You agree at all times to keep strictly confidential, and ensure that the Data Users keep confidential the information and Data pertaining to Data Subjects. In particular, You undertake not to use, or attempt to use, or permit anyone other than the Data Users to use the Data on its own or in conjunction with other data, to seek to discover the identity of any Data Subjects, to compromise or otherwise infringe the confidentiality of information on Data Subjects and their right to privacy.
You accept that the Data is protected by and subject to national and international laws, and that You are responsible for ensuring compliance with any applicable laws.
You agree that you will submit a summary report and any other information reasonably requested to the KEMRI Wellcome Trust Research Programme for the purposes of monitoring data use on the understanding that such information, data, results, and conclusions contained within will be treated as confidential information belonging to the Recipient.
This agreement is not transferable and You may not purport to assign it (in whole or in part) without the written consent of the KEMRI Wellcome Trust Research Programme.
If You commit a material breach of this Agreement or for any persistent breach of this Agreement, the KEMRI Wellcome Trust may terminate this Agreement immediately by notice in writing, without prejudice to its accrued rights and remedies.
You accept that it may be necessary for the KEMRI Wellcome Trust Research Programme to alter the terms of this Agreement from time to time in order to address new concerns. In this event, the KEMRI Wellcome Trust Research Programme will contact You to inform You of any changes and You agree that Your continued use of the Data shall be dependent on the parties entering into a variation of the Agreement.
If requested to do so by the Data Governance Committee, the KEMRI Wellcome Trust Research Programme may terminate this Agreement at any time by giving one month's notice in writing to You.
Your duty to protect the confidentiality of the Data received under this agreement shall survive termination of this Agreement and shall continue in full force and effect indefinitely.
Upon termination of this Agreement you will permanently delete all copies of the Data from your computer systems and storage devices and will ensure that all Your Data Users shall also permanently delete such copies. You may retain aggregate information on the proviso that such aggregate information does not allow any individuals or groups of individuals who are the subject of the Data to be identified with reasonable effort.
You may publish Your results arising from the use of the Data for the Agreed Purpose providing the Data itself is not disclosed. Aggregate or generic information generated from the Data may be published on the provisos that: i) such aggregate or generic information does not allow Data Subjects or groups of Data Subjects to be identified with reasonable effort; ii) no damage or distress is or is reasonably likely to be caused to any Data Subjects or groups of Data Subjects; iii) the Data will not be used in any way that could reasonably be expected to lead to ethnic stigmatisation; and iv) no attempt will be made to identify the Data Subjects.
You agree to acknowledge the KEMRI Wellcome Trust Research Programme or appropriate Programme researchers (via authorship or an acknowledgment statement) in any work based in whole or part on the Data in the following way :*"This paper/publication has used data and/or biological samples provided by the KEMRI Wellcome Trust Research programme in accordance with the consent provided by participants and approved by the KEMRI Ethics Review Committee"*.
You agree that if your application is approved, information about the proposed research use can be posted on the KEMRI Wellcome Trust Research Programme's public website. The information may include Your name and institution, the title of the project, and a non-technical summary of the research question.
You recognise that nothing in this Agreement shall operate to transfer to You any intellectual property rights relating to the Data. You have the right to develop intellectual property based on comparisons with Your own data.

Definitions in the agreement above:
"Recipient" means the principal researcher named above; "Recipient Institution" means the organisation named above at which the Recipient is employed, affiliated or enrolled; "Data Users" means those officers, employees and students of the Recipient Institution, who work directly with the Recipient and have a need to use the Data for the performance of their work with respect to the Agreed Purpose, and have agreed to comply with this Agreement, as named in the application; "Database" the KIDMS database containing clinical and demographic and health surveillance data on Data Subjects; "Data Subjects" the individuals who have contributed their data to the Database; "Data" the data within the Database; "Agreed Purpose" means the medical research purpose(s) approved by the Data Governance Committee in writing; "Publications" means, without limitation, articles published in print journals, electronic journals, reviews, books, posters and other written and verbal presentations of research.

Statement of agreement: I agree to the above conditions and limitations for sharing data as requested in this application:

Name:____AIMEE STAUNTON____________________________
Date:____04/06/20____________________
Signature:____________________

Signed on behalf of KEMRI-Wellcome Trust Research Programme:

....

Title:...Professor Philip Bejon..

Position:...Programme Executive Director...

Signature: ......[illegible]..

Date:...........29/Jun/2020...

For peer review only

Appendix 4: Data from heatmaps in Figures 1-4 showing diagnostic criteria use in common neonatal conditions.

4a. Suspected sepsis

NEONATAL SEPSIS																
	CRITERIA NUMBER															
NNU	1.	2.	3.	4.	5.	6.	7.	8.	9.	10.	11.	12.	13	14.	15.	16.
1	1.00	1.00	1.00	1.00	1.00	1.00	0.00	1.00	1.00	1.00	0.24	0.00	0.00	0.99	0.46	0.01
2	0.99	0.98	0.89	0.98	1.00	0.98	0.79	0.35	0.38	0.38	0.02	0.01	0.01	0.02	0.01	0.01
3	1.00	1.00	1.00	1.00	1.00	1.00	0.12	0.97	0.99	0.99	0.01	0.00	0.00	0.15	0.03	0.01
4	1.00	1.00	0.88	0.98	0.99	0.93	0.59	0.96	0.92	0.92	0.89	0.84	0.13	0.72	0.27	0.26
5	1.00	1.00	1.00	1.00	1.00	0.96	0.96	1.00	0.96	0.96	0.08	0.04	0.04	0.02	0.00	0.00
6	1.00	1.00	1.00	1.00	1.00	0.70	0.98	0.59	1.00	1.00	1.00	0.89	0.98	1.00	0.72	0.54
7	1.00	1.00	0.89	0.99	1.00	0.99	0.73	0.96	0.76	0.74	0.70	0.36	0.35	0.75	0.37	0.19

For peer review only

4b. Respiratory conditions

RESPIRATORY CONDITIONS															
	CRITERIA NUMBER														
NNU	1	2.	3.	4.	5.	6.	7.	8.	9.	10.	11.	12.	13.	14.	15.
1	1.00	1.00	1.00	1.00	1.00	0.99	1.00	1.00	1.00	1.00	1.00	1.00	0.51	0.50	0.97
2	0.93	0.88	0.95	0.91	0.93	0.98	0.99	0.99	0.99	0.99	0.99	0.99	0.10	0.04	0.78
3	1.00	1.00	1.00	1.00	1.00	1.00	1.00	1.00	1.00	1.00	1.00	1.00	0.47	0.37	1.00
4	1.00	0.98	0.97	0.98	0.99	0.96	1.00	1.00	0.99	0.96	1.00	0.99	0.26	0.26	0.85
5	0.56	1.00	0.89	1.00	1.00	1.00	1.00	1.00	1.00	1.00	1.00	1.00	0.06	0.06	1.00
6	1.00	0.98	0.99	0.99	0.99	0.99	0.99	0.99	0.99	0.99	0.99	0.99	0.14	0.14	0.99
7	1.00	1.00	1.00	1.00	1.00	1.00	1.00	1.00	1.00	0.96	0.87	0.96	0.39	0.39	0.96

For peer review only

*4*c. Birth asphyxia

BIRTH ASPHYXIA						
	CRITERIA NUMBER					
NNU	1.	2.	3.	4.	5.	6.
1	1.00	0.02	1.00	0.63	1.00	0.00
2	0.75	0.75	0.84	0.07	0.72	0.04
3	1.00	0.95	1.00	0.10	1.00	0.00
4	0.99	0.88	0.96	0.77	0.98	0.23
5	1.00	0.85	0.88	0.78	0.98	0.39
6	0.91	0.73	0.93	0.95	1.00	0.84
7	0.91	0.76	0.73	0.49	0.95	0.51

For peer review only

4d. Abdominal conditions

ABDOMINAL CONDITIONS																						
	CRITERIA NUMBER																					
NNU	1.	2.	3.	4.	5.	6.	7.	8.	9.	10.	11.	12.	13.	14.	15.	16.	17.	18.	19.	20.	21.	22
1	1.00	1.00	1.00	1.00	1.00	1.00	1.00	1.00	1.00	1.00	1.00	1.00	1.00	1.00	1.00	0.33	0.33	0.33	0.33	0.33	0.33	0.33
2	1.00	1.00	1.00	1.00	1.00	1.00	1.00	1.00	1.00	1.00	1.00	0.75	0.75	0.75	0.75	0.00	0.75	0.75	0.75	0.75	0.75	0.75
3	1.00	0.67	0.83	1.00	1.00	1.00	1.00	1.00	1.00	1.00	1.00	1.00	1.00	1.00	1.00	0.67	0.67	0.67	0.50	0.67	0.67	0.67
4	1.00	0.94	0.78	0.88	0.97	0.94	1.00	0.81	0.94	1.00	1.00	0.97	0.94	0.94	0.97	0.97	0.75	0.72	0.69	0.75	0.78	0.72
5	0.67	0.67	0.67	0.67	0.67	0.67	0.67	0.67	0.67	0.67	0.67	0.67	0.67	0.67	0.67	0.67	0.00	0.00	0.00	0.00	0.00	0.00
6	1.00	1.00	1.00	1.00	1.00	1.00	1.00	1.00	1.00	1.00	1.00	1.00	0.93	0.93	0.93	0.93	0.93	1.00	1.00	1.00	0.93	0.93
7	1.00	1.00	1.00	1.00	1.00	1.00	0.89	0.89	0.89	1.00	1.00	0.67	0.56	0.56	0.56	0.78	0.56	0.33	0.44	0.56	0.56	0.44

1. For Appendix 4 data: 1.00 means in 100% of cases the diagnostic criterion was used by clinicians

For peer review only

The RECORD statement – checklist of items, extended from the STROBE statement, that should be reported in observational studies using routinely collected health data.

	Item No.	STROBE items	Location in manuscript where items are reported	RECORD items	Location in manuscript where items are reported
**Title and abstract**					
	1	(a) Indicate the study's design with a commonly used term in the title or the abstract (b) Provide in the abstract an informative and balanced summary of what was done and what was found	Page 1-2	RECORD 1.1: The type of data used should be specified in the title or abstract. When possible, the name of the databases used should be included.<br><br>RECORD 1.2: If applicable, the geographic region and timeframe within which the study took place should be reported in the title or abstract.<br><br>RECORD 1.3: If linkage between databases was conducted for the study, this should be clearly stated in the title or abstract.	Page 2
**Introduction**					
Background rationale	2	Explain the scientific background and rationale for the investigation being reported	Page 4-5		
Objectives	3	State specific objectives, including any prespecified hypotheses	Page 4-5		
**Methods**					
Study Design	4	Present key elements of study design early in the paper	Page 5-6		
Setting	5	Describe the setting, locations, and relevant dates, including periods of recruitment, exposure, follow-up, and data collection	Page 5-6		

For peer review only

Participants	6	*(a) Cohort study* - Give the eligibility criteria, and the sources and methods of selection of participants. Describe methods of follow-up<br>*Case-control study* - Give the eligibility criteria, and the sources and methods of case ascertainment and control selection. Give the rationale for the choice of cases and controls<br>*Cross-sectional study* - Give the eligibility criteria, and the sources and methods of selection of participants<br><br>*(b) Cohort study* - For matched studies, give matching criteria and number of exposed and unexposed<br>*Case-control study* - For matched studies, give matching criteria and the number of controls per case		RECORD 6.1: The methods of study population selection (such as codes or algorithms used to identify subjects) should be listed in detail. If this is not possible, an explanation should be provided.<br><br>RECORD 6.2: Any validation studies of the codes or algorithms used to select the population should be referenced. If validation was conducted for this study and not published elsewhere, detailed methods and results should be provided.<br><br>RECORD 6.3: If the study involved linkage of databases, consider use of a flow diagram or other graphical display to demonstrate the data linkage process, including the number of individuals with linked data at each stage.	Page 5
Variables	7	Clearly define all outcomes, exposures, predictors, potential confounders, and effect modifiers. Give diagnostic criteria, if applicable.	Page 6	RECORD 7.1: A complete list of codes and algorithms used to classify exposures, outcomes, confounders, and effect modifiers should be provided. If these cannot be reported, an explanation should be provided.	n/a
Data sources/ measurement	8	For each variable of interest, give sources of data and details of methods of assessment (measurement).<br>Describe comparability of assessment methods if there is more than one group	Page 5-6		

For peer review only - http://bmjopen.bmj.com/site/about/guidelines.xhtml

Bias	9	Describe any efforts to address potential sources of bias	Page 5		
Study size	10	Explain how the study size was arrived at	Page 5		
Quantitative variables	11	Explain how quantitative variables were handled in the analyses. If applicable, describe which groupings were chosen, and why	Page 6		
Statistical methods	12	(a) Describe all statistical methods, including those used to control for confounding<br>(b) Describe any methods used to examine subgroups and interactions<br>(c) Explain how missing data were addressed<br>(d) *Cohort study* - If applicable, explain how loss to follow-up was addressed<br>*Case-control study* - If applicable, explain how matching of cases and controls was addressed<br>*Cross-sectional study* - If applicable, describe analytical methods taking account of sampling strategy<br>(e) Describe any sensitivity analyses	Page 6		
Data access and cleaning methods		..		RECORD 12.1: Authors should describe the extent to which the investigators had access to the database population used to create the study population.	Page 5-6

For peer review only

				RECORD 12.2: Authors should provide information on the data cleaning methods used in the study.	
Linkage		..		RECORD 12.3: State whether the study included person-level, institutional-level, or other data linkage across two or more databases. The methods of linkage and methods of linkage quality evaluation should be provided.	Page 5
**Results**					
Participants	13	(a) Report the numbers of individuals at each stage of the study (*e.g.*, numbers potentially eligible, examined for eligibility, confirmed eligible, included in the study, completing follow-up, and analysed)<br>(b) Give reasons for non-participation at each stage.<br>(c) Consider use of a flow diagram	Page 6-7	RECORD 13.1: Describe in detail the selection of the persons included in the study (*i.e.*, study population selection) including filtering based on data quality, data availability and linkage. The selection of included persons can be described in the text and/or by means of the study flow diagram.	Page 5
Descriptive data	14	(a) Give characteristics of study participants (*e.g.*, demographic, clinical, social) and information on exposures and potential confounders<br>(b) Indicate the number of participants with missing data for each variable of interest<br>(c) *Cohort study* - summarise follow-up time (*e.g.*, average and total amount)	Page 6-7<br>Table 1-2		
Outcome data	15	*Cohort study* - Report numbers of outcome events or summary measures over time<br>*Case-control study* - Report numbers in each exposure	n/a		

For peer review only

		category, or summary measures of exposure<br>*Cross-sectional study* - Report numbers of outcome events or summary measures			
Main results	16	(a) Give unadjusted estimates and, if applicable, confounder-adjusted estimates and their precision (e.g., 95% confidence interval). Make clear which confounders were adjusted for and why they were included<br>(b) Report category boundaries when continuous variables were categorized<br>(c) If relevant, consider translating estimates of relative risk into absolute risk for a meaningful time period	n/a		
Other analyses	17	Report other analyses done—e.g., analyses of subgroups and interactions, and sensitivity analyses	Page 6-8		
**Discussion**					
Key results	18	Summarise key results with reference to study objectives	Page 8-10		
Limitations	19	Discuss limitations of the study, taking into account sources of potential bias or imprecision. Discuss both direction and magnitude of any potential bias	Page 10	RECORD 19.1: Discuss the implications of using data that were not created or collected to answer the specific research question(s). Include discussion of misclassification bias, unmeasured confounding, missing data, and changing eligibility over time, as they pertain to the study being reported.	n/a
Interpretation	20	Give a cautious overall interpretation of results considering objectives,	Page 11		

For peer review only

		limitations, multiplicity of analyses, results from similar studies, and other relevant evidence			
Generalisability	21	Discuss the generalisability (external validity) of the study results	Page 10		
**Other Information**					
Funding	22	Give the source of funding and the role of the funders for the present study and, if applicable, for the original study on which the present article is based	Page 6		
Accessibility of protocol, raw data, and programming code		..		RECORD 22.1: Authors should provide information on how to access any supplemental information such as the study protocol, raw data, or programming code.	Appendices Page 12

*Reference: Benchimol EI, Smeeth L, Guttmann A, Harron K, Moher D, Petersen I, Sørensen HT, von Elm E, Langan SM, the RECORD Working Committee. The REporting of studies Conducted using Observational Routinely-collected health Data (RECORD) Statement. *PLoS Medicine* 2015; in press.

*Checklist is protected under Creative Commons Attribution (CC BY) license.

For peer review only

BMJ Open

A prospective observational study of the challenges in diagnosing common neonatal conditions in Nigeria and Kenya

Journal:	*BMJ Open*
Manuscript ID	bmjopen-2022-064575.R2
Article Type:	Original research
Date Submitted by the Author:	16-Oct-2022
Complete List of Authors:	Staunton, Aimee; Liverpool School of Tropical Medicine,<br>Nabwera, Helen; Liverpool School of Tropical Medicine, International Public Health; Alder Hey Children's NHS Foundation Trust<br>Allen, Stephen ; Liverpool School of Tropical Medicine, Child Health; Alder Hey Children's NHS Foundation Trust<br>Tongo, Olukemi; University College Hospital Ibadan<br>Akindolire, Abimbola ; University College Hospital Ibadan<br>Abdulkadir, Isa; Ahmadu Bello University Teaching Hospital<br>Ezeaka, Chinyere; University of Lagos College of Medicine, Paediatrics<br>Ezenwa, Beatrice; University of Lagos College of Medicine, Paediatrics<br>Fajolu, Iretiola; College of Medicine University of Lagos, Department of Paediatrics; Lagos University Teaching Hospital<br>Imam, Zainab; Lagos State University Teaching Hospital<br>Umoru , Dominic ; Maitama District Hospital<br>Otieno, Walter; Maseno University; Jaramogi Oginga Odinga Teaching and Referral Hospital, Kisumu, Kenya<br>Nalwa, Grace; Maseno University, Department of Paediatrics and Child Health, ; Jaramogi Oginga Odinga Teaching and Referral Hospital, Kisumu, Kenya<br>Olwala, Macrine; Jaramogi Oginga Odinga Teaching and Referral Hospital, Kisumu, Kenya<br>Talbert, Alison; KEMRI-Wellcome Trust Research Programme<br>Andang'o, Pauline; Maseno University<br>Mwangome, MK; KEMRI-Wellcome Trust Research Programme,<br>Abubakar, Ismaela; Liverpool School of Tropical Medicine<br>Embleton, Nicholas; Newcastle University; Newcastle Upon Tyne Hospitals NHS Trust
**Primary Subject Heading**:	Public health
Secondary Subject Heading:	Diagnostics, Global health, Paediatrics, Public health
Keywords:	Health policy < HEALTH SERVICES ADMINISTRATION & MANAGEMENT, Public health < INFECTIOUS DISEASES, NEONATOLOGY, Paediatric gastroenterology < PAEDIATRICS

I, the Submitting Author has the right to grant and does grant on behalf of all authors of the Work (as defined in the below author licence), an exclusive licence and/or a non-exclusive licence for contributions from authors who are: i) UK Crown employees; ii) where BMJ has agreed a CC-BY licence shall apply, and/or iii) in accordance with the terms applicable for US Federal Government officers or employees acting as part of their official duties; on a worldwide, perpetual, irrevocable, royalty-free basis to BMJ Publishing Group Ltd ("BMJ") its licensees and where the relevant Journal is co-owned by BMJ to the co-owners of the Journal, to publish the Work in this journal and any other BMJ products and to exploit all rights, as set out in our licence.

The Submitting Author accepts and understands that any supply made under these terms is made by BMJ to the Submitting Author unless you are acting as an employee on behalf of your employer or a postgraduate student of an affiliated institution which is paying any applicable article publishing charge ("APC") for Open Access articles. Where the Submitting Author wishes to make the Work available on an Open Access basis (and intends to pay the relevant APC), the terms of reuse of such Open Access shall be governed by a Creative Commons licence – details of these licences and which Creative Commons licence will apply to this Work are set out in our licence referred to above.

Other than as permitted in any relevant BMJ Author's Self Archiving Policies, I confirm this Work has not been accepted for publication elsewhere, is not being considered for publication elsewhere and does not duplicate material already published. I confirm all authors consent to publication of this Work and authorise the granting of this licence.

For peer review only

A prospective observational study of the challenges in diagnosing common neonatal conditions in Nigeria and Kenya

Aimee Staunton* [1], Helen M Nabwera[1,2], Stephen J Allen[1,2], Olukemi O Tongo[3], Abimbola E Akindolire[3], Isa Abdulkadir[4], V. Chinyere Ezeaka[5], Beatrice N Ezenwa[5], Iretiola B Fajolu[5,6], Zainab O Imam[6], Dominic D Umoru[7], Walter Otieno[8,9], Grace M Nalwa[8,9], Macrine Olwala[9], Alison W Talbert[10], Pauline EA Andang'o[8], Martha K Mwangome[10], Ismaela Abubakar[1], Nicholas D Embleton[11,12], on behalf of the Neonatal Nutrition Network (NeoNuNet)**

1. Liverpool School of Tropical Medicine, Pembroke place, Liverpool, L3 5QA, UK
2. Alder Hey Children's Hospital NHS Trust, E Prescot Rd, Liverpool L14 5AB, UK
3. University College Hospital, Queen Elizabeth Road, Ibadan, Nigeria
4. Ahmadu Bello University Teaching Hospital, Shika, Zaria, Nigeria
5. College of Medicine, University of Lagos, Idi-Araba, Lagos, Nigeria; Lagos University Teaching Hospital, Idi-Araba, Lagos, Nigeria.
6. Lagos State University Teaching Hospital, Ikeja, Lagos, Nigeria
7. Maitama District Hospital, 61 Aguiyi Ironsi St, Maitama, Abuja, Nigeria
8. Maseno University, Maseno, Kenya
9. Jaramogi Oginga Odinga Teaching and Referral Hospital, Jomo Kenyatta Highway Kaloleni Kisumu KE, Central, Kenya
10. KEMRI-Wellcome Trust Research Programme, PO Box 230-80108, Kilifi, Kenya
11. Newcastle University, Newcastle upon Tyne NE1 7RU, UK
12. The Newcastle upon Tyne Hospitals NHS Foundation Trust, High Heaton, NE7 7DN, UK

**Corresponding author, contact: aimee.staunton@ntlworld.com*

***Full Neonatal Nutrition Network members list in Appendix 1*

Short title: A prospective observational study of the challenges in diagnosing common neonatal conditions in Nigeria and Kenya

Number of tables: *2*

Number of figures: *1*

For peer review only - http://bmjopen.bmj.com/site/about/guidelines.xhtml

For peer review only

Abstract

Objectives: Accurate and timely diagnosis of common neonatal conditions is crucial for reducing neonatal deaths. In low- and middle-income countries with limited resources, there is sparse information on how neonatal diagnoses are made. The aim of this study was to describe the diagnostic criteria used for common conditions in neonatal units (NNUs) in Nigeria and Kenya.

Design: Prospective observational study. Standard case report forms for suspected sepsis, respiratory disorders, birth asphyxia and abdominal conditions were co-developed by the Neonatal Nutrition Network* collaborators. Clinicians completed forms for all admissions to their NNU. Key data was displayed using heatmaps.

Setting: Five NNUs in Nigeria and two in Kenya comprising the Neonatal Nutrition Network.

Participants: 2851 neonates, which included all neonates admitted to the seven NNUs over a six month period.

Results: 1230 (43.1%) neonates had suspected sepsis, 874 (30.6%) respiratory conditions, 587 (20.6%) birth asphyxia and 71 (2.5%) abdominal conditions. For all conditions and across all NNUs, clinical criteria were used consistently with sparse use of laboratory and radiological criteria.

Conclusion: Our findings highlight the reliance on clinical criteria and extremely limited use of diagnostic technologies for common conditions in NNUs in sub-Saharan Africa. This has implications for the management of neonatal conditions which often have overlapping clinical features. Strategies for implementation of diagnostic pathways and investment in affordable and sustainable diagnostics are needed to improve care for these vulnerable infants.

* https://www.lstmed.ac.uk/nnu

For peer review only - http://bmjopen.bmj.com/site/about/guidelines.xhtml

Strengths and limitations

Strengths

- The use of heatmaps to display diagnostic pathways highlights key patterns in the diagnostic criteria used by clinicians.
- Meticulous data collection, designed and supervised by local clinicians and international researchers, has provided fresh insights into diagnostic practice in this under researched population.

Limitations

- Most network NNUs were urban, tertiary level facilities serving families that could likely better afford investigations so that our findings may not be generalisable to NNUs serving poorer populations.

For peer review only

Background

The majority of the 2.4 million global neonatal deaths (< 28 days) in 2019 were due to three largely preventable or treatable conditions: complications of preterm birth, intrapartum-related events and sepsis (1). Accurate and timely diagnosis of these common conditions is crucial for reducing mortality (1, 2). The clinical assessments and decisions from presentation to diagnosis, referred to as diagnostic pathways, are known to influence outcomes (3). Although access to diagnostics is included in one of the six WHO health system building blocks, there are limited data on diagnostic pathways and how these influence outcomes in neonatal care in low-and middle-income countries (LMICs) (4-6).

In Nigeria and Kenya, concerted actions are required to reduce neonatal mortality (7). Progress in reducing neonatal mortality rates (NMR; the number of deaths occurring 0-<28 days per 1000 live births) has been slow in both countries (8). Between 1990 and 2019, NMR in Nigeria decreased by 28% from 50 to 36 per 1000 and in Kenya by 25% from 28 to 21 per 1000 (1). Consequently, both countries are unlikely to meet the sustainable development goal (SDG 3.2) for neonatal mortality and are thus classified as priority countries in the Countdown to 2030 initiative (9).

In the context of robust health systems, national or regional guidelines may support the standardisation and accuracy of diagnoses as a means of improving care and reducing mortality in highly vulnerable hospitalised infants (10, 11). Whilst both countries have policies on newborn health, only Kenya had national guidelines for the management of common neonatal conditions at the time of the study (12, 13). However, there is limited information on the impact of guidelines in low-resource settings, where challenges with resources for health are greatest (14).

Encouraged by international efforts to improve access to diagnostics and the sparse information on how common neonatal diagnoses are made (5, 15-17), we sought to describe

For peer review only - http://bmjopen.bmj.com/site/about/guidelines.xhtml

the diagnostic criteria used by clinicians in seven neonatal units (NNUs) in Nigeria and Kenya participating in the Neonatal Nutrition Network (NeoNuNet https://www.lstmed.ac.uk/nnu). Network members can be found in appendix 1.

Methods

Design and setting

In this prospective observational study, we collected information from five NNUs in Nigeria and two in Kenya; five providing tertiary level care, two providing district level care and all but one situated in major cities (18). All NNUs provided supportive feeding, intravenous fluids, antibiotics, phototherapy and bubble continuous positive airway pressure (CPAP). Two out of seven NNUs provide free neonatal healthcare services. Further details of the NNUs are available in appendix 2.

Data Collection

Case report forms were developed for birth asphyxia, respiratory disorders, abdominal conditions, and suspected sepsis with reference to international and local guidelines. Respiratory conditions included respiratory distress syndrome, transient tachypnoea of the newborn, pneumonia and meconium aspiration. Abdominal conditions included necrotizing enterocolitis (NEC), dysmotility, septic ileus and focal intestinal perforation. Suspected sepsis was defined as clinical assessment resulting in starting or changing antibiotic treatment. Over a 6-month period in each NNU between September 2018 and April 2019, clinicians completed case report forms for all admissions and each episode of illness. A new episode occurred if the infant has been symptom free for 48 hours or more after any previous episode. Only the first episode of each illness was included in this analysis. Demographic and clinical information was extracted from the main NeoNuNet database. For out-born infants in whom birthweight was not known, weight on admission was used instead of birthweight. Completed case report forms were reviewed by a senior clinician. Data clerks

For peer review only

entered anonymised data into a password protected REDCap database hosted by the Liverpool School of Tropical Medicine (LSTM) (10, 19). Ethical approval was granted (appendix 3).

Patient and Public Involvement

Study participants and the public were not involved in the design or undertaking of the study.

Data management and analysis

Data was imported to SPSS version 25 (20) for analysis. Categorical variables were presented as frequencies and percentages. Diagnostic criteria were presented using heatmaps (Microsoft Office 16 Excel). Each criterion was classified as either used (with either a positive or negative finding) or not used. Criteria were grouped into clinical, laboratory, clinical/laboratory, and radiological criteria. The 'COUNTIFS' function was used to calculate the proportion of cases in which the criterion was used, and a divergent colour scheme was applied to the heatmap cells.

For peer review only

<u>Results</u>

Over the 6 months' data collection period, 2851 neonates were admitted to the seven NNUs. The majority of newborns were male (1626; 57.1%), most had been delivered in a health facility (2600; 91.2%) and just over half were vaginal unassisted deliveries (1586; 55.7%). Overall, 1261 (45.3%) were preterm (<37 weeks' gestation), 1405 (49.3%) were low birth weight (<2.50kg) and 473 (16.6%) infants died (Table 1). The proportions of very preterm (VPT, <32 weeks' gestation) or very low birth weight (VLBW, <1500g) infants varied considerably across the NNUs (Table 2).

For peer review only - http://bmjopen.bmj.com/site/about/guidelines.xhtml

Table 1: Newborn characteristics of 2851 admissions[1]

**Variable**	
Male gender[2]	1626 (57.1)
Place of delivery	
• Health facility	2600 (91.2)
• Home	165 (5.8)
• Other	86 (3.0)
Mode of delivery[3]	
• Vaginal unassisted	1586 (55.7)
• Caesarean section	1160 (40.7)
• Vaginal assisted/ instrumental	101 (3.5)
Gestational age in weeks[4] Median (IQR)	37.0 (33.0-39.0)
Gestational age category[4]	
• Extreme preterm (<28 weeks)	122 (4.3)
• Very preterm (28-<32 weeks)	378 (13.3)
• Moderately preterm (32-<37 weeks)	761 (26.7)
Birth weight or weight on admission in kg[5] Median (IQR)	2.50 (1.67-31.7)
Birth weight or weight on admission category[5] (n; %)	
• ELBW (<1000g)	116 (4.1)
• VLBW (1000-1499g)	393 (13.8)
• LBW (1500-2499g)	896 (31.5)
Antibiotics given after birth[6]	1294 (45.4)
Final infant outcome[7]	
• Discharged with no morbidities	2170 (76.1)
• Discharged with morbidities	89 (3.1)
• Transferred out	43 (1.5)
• Absconded/ discharged against medical advice	55 (1.9)
• Died	473 (16.6)

Notes

1. Variables are shown as number (%) unless otherwise stated
2. Gender missing in 2
3. Mode of delivery missing in 4

For peer review only - http://bmjopen.bmj.com/site/about/guidelines.xhtml

4. Gestational age missing for 68
5. Birthweight and weight on admission missing in 3
6. Prophylactic antibiotics missing in 36
7. Final infant outcome missing in 21

For peer review only

For peer review only - http://bmjopen.bmj.com/site/about/guidelines.xhtml

Table 2: Admission characteristics and frequency of common conditions according to neonatal unit[1,2]

Condition	Kenya		Nigeria					Total
	1	2	3	4	5	6	7	
Total admissions	291	613	181	732	384	238	412	2851
Extreme or very preterm (<32 weeks' gestation)	52 (17.9)	94 (16.3)	9 (5.1)	124 (17.4)	142 (37.1)	69 (29.1)	10 (2.5)	500 (27.8)[3]
Extreme or very low birth/admission weight (<1500g)	60 (20.6)	107 (17.5)	13 (3.3)	104 (14.2)	128 (33.3)	69 (29.0)	28 (6.8)	509 (28.5)[4]
Suspected sepsis	132 (45.4)	421 (68.7)	68 (37.6)	405 (53.3)	49 (12.8)	46 (19.3)	109 (26.5)	1230 (43.1)
Respiratory conditions	78 (26.8)	364 (59.4)	19 (10.5)	210 (28.7)	18 (4.7)	162 (68.1)	23 (5.6)	874 (30.7)
Birth asphyxia	48 (16.5)	189 (30.8)	20 (11.0)	190 (26.0)	41 (10.7)	44 (18.5)	55 (13.3)	587 (20.6)
Abdominal conditions	3 (1.0)	4 (0.7)	6 (3.3)	32 (4.4)	3 (0.8)	14 (5.9)	9 (2.2)	71 (2.5)

Notes:

1. Data are shown as number (%)
2. More than one condition was recorded in many infants
3. Gestation was missing for 35 infants in unit 2, 5 in unit 3, 20 in unit 4, 1 each in units 5 and 6 and 6 in unit 7
4. Birth/admission weight was missing for 2 infants in unit 2 and 1 infant in unit 7

For peer review only - http://bmjopen.bmj.com/site/about/guidelines.xhtml

Diagnostic criteria

Overall, forms were filled in for 1230 (43.1%) newborns diagnosed with suspected sepsis, 874 (30.6%) with respiratory conditions, 587 (20.6%) with birth asphyxia and 71 (2.5%) with abdominal conditions (Table 2). The frequency of these diagnoses varied considerably between NNUs including those with similar proportions of VPT or VLBW infants. Overall, across all NNUs, diagnoses were based mainly on clinical criteria and a full blood count with limited use of other laboratory assays or radiological investigations (*Fig 1a-1d*).

For the diagnosis of suspected neonatal sepsis, clinical criteria were used uniformly in all NNUs (*Fig 1a*). The exceptions were hypotension where use varied from 0% to 98% (Appendix 4a). Assessment of glucose intolerance, often using a point of care test, and parameters from a full blood count were the most used laboratory criteria. However, other laboratory parameters requiring biochemical analyses and microbiology were used much less frequently

overall.

Similarly, for respiratory conditions, clinical criteria were used uniformly across NNUs (*Fig 1b; appendix 4b*). In marked contrast to the limited use of laboratory analyses, the use of chest

Xray appeared to be good overall but with some variability between NNUs.

The diagnosis of birth asphyxia was based on clinical evidence of encephalopathy and exclusion of other aetiologies. Evidence of multiorgan dysfunction was also used although this may have been based on the clinical components (acute kidney injury, respiratory distress, circulatory collapse and disseminated intravascular coagulation) rather than laboratory analyses. In contrast, use of cranial ultrasound scan varied widely ranging from 0-84% (Fig 1c; appendix 4c).

For peer review only - http://bmjopen.bmj.com/site/about/guidelines.xhtml

For the infants with an abdominal condition, of which about half were diagnosed as NEC (36/71, 50.7%), a range of clinical criteria were used in nearly all cases in most NNUs. Basic laboratory criteria derived from a full blood count were also frequently used but use of parameters of metabolic acidosis was more variable. Overall, radiology criteria were used less frequently (*Fig 1d, appendix 4d).*

Discussion

This study enabled us to explore diagnostic pathways and identify key challenges in the diagnosis of common neonatal conditions in the context of limited resources. Clinicians were generally constrained to a symptoms-based, clinical orientated diagnostic approach. This is consistent with the widely available WHO Pocket Book which encourages a syndromic approach and is a common source of diagnostic guidelines (21). However, the Pocket Book is targeted at first level referral hospitals, whereas greater access to diagnostics is required for higher-level care. Furthermore, for some neonatal conditions such as respiratory distress syndrome, a clinical diagnosis may be appropriate; however, given the overlapping features between neonatal conditions and the potential lack of adequate clinical history for out-born patients in particular, laboratory and radiological investigations are important to support accurate diagnosis.

The consistent use of a wide range of clinical criteria across the NNUs was a positive finding suggesting uniformity in clinical practice. This is likely supported by use of national neonatal clinical guidelines although these were available in Kenya only. National paediatric diagnostic guidelines have been found to optimise practice in high-income settings; however, this may be more challenging in LMICs where diagnostic resources and skilled workforce remain inadequate making consistent adherence to clinical guidelines difficult (22, 23). This is further complicated by political instability, healthcare strikes and high staff turnover due to

For peer review only - http://bmjopen.bmj.com/site/about/guidelines.xhtml

poor working conditions which may result in gaps in training on appropriate use of diagnostic technologies when they are available (24, 25).

In marked contrast with clinical criteria, the limited use of laboratory investigations beyond a full blood count, shows that there were challenges implementing standardised diagnostic pathways. There was obvious overlap between conditions, so that investigations such as blood culture were used infrequently in both suspected sepsis and respiratory illness. Although some laboratory procedures were available in individual hospitals, in our experience, this is often short-term due to dependency on donor or research support. The limited use of laboratory investigations within the NNUs may be due to a lack of affordable and quality assured laboratory and specimen transport services, which are common barriers in LMICs (5, 26). This is likely to have implications on the accuracy of diagnoses, since a syndromic diagnostic approach has been linked to lower specificity, thereby resulting in more false positive cases (27). The lack of laboratory diagnostic support in our study would likely result in the overdiagnosis of neonatal sepsis. A direct consequence is the overuse of antibiotics in this population, which could drive antimicrobial resistance (28). Use of laboratory investigations to complement clinical assessment, as well as antibiotic stewardship programmes, would likely help (29).

Respiratory conditions such as respiratory distress syndrome were typically diagnosed using clinical criteria but also supported by chest X rays which are critically important in distinguishing the cause of respiratory distress, which includes a number of pathologies with overlapping clinical signs (30). The diagnosis of birth asphyxia was also based mainly on clinical criteria and with limited use of cranial ultrasound. In addition to diagnosis, laboratory support with determining acid-base balance and blood electrolytes is required in the

For peer review only - http://bmjopen.bmj.com/site/about/guidelines.xhtml

selection of infants for treatment approaches such as therapeutic hypothermia and monitoring treatment response (31).

In contrast to the use of X ray in respiratory conditions, abdominal Xray, a key investigation for the diagnosis of conditions such as NEC, was only used consistently in one unit. The limited use may be due to more severe illness in infants with suspected NEC and the lack of mobile X ray machines. Previous research in LMIC settings has reported that diagnosis is impaired by a lack of abdominal X rays, since even when X ray machines are available and functioning, they are often too expensive for patients and families to afford (32). This is likely here since only two out of the seven NNUs provide free of charge neonatal health services. This means that diagnosis is very difficult for conditions such as NEC where specific X ray findings are critically important (33).

Given the consistent use of clinical criteria across the NNUs, the limited use of laboratory and radiological investigations likely contributed to the marked differences between units in the frequencies of diagnoses. This would appear to be less likely due to differences in case mix, since variability in the frequency of diagnoses occurred between NNUs with similar proportions of small and preterm newborns in whom these conditions are common. These challenges highlight the need for sustainable and affordable diagnostic tests suitable for use in resource limited settings for accurate and timely diagnosis and to reliably assess the frequency of the main causes of neonatal mortality. This should include point-of-care diagnostic tests designed for use in LMICs which can provide rapid results and reduce the workload for laboratory services (34, 35).

The limitations of our study relate mainly to the generalisability of our findings to other settings. Most of the network NNUs in this study were urban and tertiary level which are often inaccessible to disadvantaged families due to higher access costs. It is therefore likely

For peer review only - http://bmjopen.bmj.com/site/about/guidelines.xhtml

that the families in our study could better afford some investigations than many in Nigeria and Kenya, limiting the generalisability of our findings. In addition, referral patterns, case mix and the experience of clinicians working within the NNUs in making diagnoses may also differ with other NNUs. Finally, although forms were completed by different cadres of clinical staff with varying levels of experience and senior review was limited to ensuring completeness of data, the use of the standardised study forms and review of data by senior clinicians may have encouraged the consistent use of clinical criteria in our study thereby improving clinical diagnoses compared with usual clinical practice.

Conclusions

Our findings demonstrate that diagnostic pathways for common conditions in NNUs in Nigeria and Kenya were hindered by the limited use of laboratory and radiological technologies. Improving capacity to diagnose common conditions rapidly and reliably in hospitalised newborns should be a key priority of future healthcare quality improvement initiatives. The development of national plans to increase the availability, accessibility, and affordability of point of care and other diagnostic investigations is paramount since the need for out-of-pocket health spending hinders compliance to clinical guidelines (5). This will be dependent on infrastructure strengthening and appropriate staff training and education. Our findings will inform the development and implementation of integrated context-relevant best practice guidelines for neonatal care in low- resource settings. Further research is necessary to examine the impact of such guidelines on neonatal health in these settings.

For peer review only

Acknowledgements

We would like to thank our colleagues who contributed to the clinical care and collection of data at the Network neonatal units. We would also like to thank colleagues at the Nigerian Society of Neonatal Medicine, the Kenya Paediatric Association and the Ministries of Health

For peer review only - http://bmjopen.bmj.com/site/about/guidelines.xhtml

in Nigeria and Kenya, who provided us with support and advice as we were setting up this study. We thank the mothers and families immensely for participating in this study with their infants. This study is published with the permission of the Director of Kenya Medical Research Institute.

Contributorship statement

Stephen J Allen conceived the study. Aimee Staunton, Stephen J Allen, Helen M Nabwera, Olukemi O Tongo, Abimbola E Akindolire, Isa Abdulkadir, V. Chinyere Ezeaka, Beatrice N Ezenwa, Iretiola B Fajolu, Zainab O Imam, Dominic D Umoru, Walter Otieno, Grace M Nalwa, Macrine Olwala, Alison W Talbert, Pauline EA Andandg'o, Martha K Mwangome, Ismaela Abubakar and Nicholas D Embleton designed the study and each read, helped to critically revise and approved the final manuscript version and agree to be accountable for the accuracy and integrity of the manuscript and to answer any questions in relation to the work. Aimee Staunton analysed the data. Aimee Staunton, Stephen J Allen and Helen M Nabwera prepared the manuscript. Aimee Staunton is the corresponding author responsible for submission, peer review and publication processes.

Open access

This is an open access article distributed in accordance with the Creative Commons Attribution 4.0 Unported (CCBY4.0) license, which permits others to copy, redistribute, remix, transform and build upon this work for any purpose, provided the original work is properly cited, a link to the licence is given, and indication of whether changes were made.

Data sharing

Further data from the heatmaps is available in appendix 4. Requests for access to further data should be addressed to the corresponding author.

For peer review only - http://bmjopen.bmj.com/site/about/guidelines.xhtml

Ethics approval

Ethical approval was granted by the Research Ethics Committees at LSTM (18–0210), Lagos

University Teaching Hospital Health REC (AMD/DCST/HREC/APP/2514), the Jaramogi Oginga Odinga Teaching and Referral Hospital (ERC.IB/VOL.1/510), University College Hospital Ibadan (UI/EC/18/0446), Massey Street Children's Hospital (LSHSC/2222/VOL.VI[B]/185), Ahmadu Bello University Teaching Hospital (ABUTH/HZ/HREC/D37/2018), Maitama District Hospital (FHREC/2018/01/108/19-09-18), the Kenya Medical Research Institute-Scientific and Ethics Review Unit (KEMRI/SERU/CGMR-C/120/3740) (10) (Appendices 3a-3b).

Funding

This project was funded by a grant from the MRC Confidence in Global Nutrition and Health Research scheme (grant reference MC_PC_MR/R019789/1); the funders had no role in the design or conduction of the study.

Conflict of Interests Statement

None to declare.

Figures

Figure 1: Heatmaps showing the diagnostic criteria used for common neonatal conditions.

For peer review only - http://bmjopen.bmj.com/site/about/guidelines.xhtml

References

1. United Nations Inter-agency Group for Child Mortality Estimation (UN IGME). Levels and Trend in Child Mortality Report. 2020.
2. Healthy Newborn Network. Explore Countries- Nigeria. healthynewbornnetwork.org.2017 [Available from: https://www.healthynewbornnetwork.org/country/nigeria/.
3. National Academies of Sciences Engineering and Medicine. Improving diagnosis in health care: National Academies Press; 2015.
4. World Health Organization. Everybody's business-strengthening health systems to improve health outcomes: WHO's framework for action. 2007.
5. Fleming K, Horton S, Wilson L, et al. The Lancet Commission on diagnostics: transforming access to diagnostics. *The Lancet*. 2021;398:1997-2050.
6. Pius S, Bello M. Neonatal Septicaemia in Poor Resource Settings. *Pediatric Infect Dis*. 2017;2:2537-0282.
7. The World Bank. World Bank Country and Lending Groups. worldbank.org.2018 [Available from: https://datahelpdesk.worldbank.org/knowledgebase/articles/906519.
8. World Health Organization. Progress Report: Reaching every newborn national 2020 milestones: country progress, plans and moving forward. 2018.
9. Boerma T, Requejo J, Victora C, et al. Countdown to 2030: tracking progress towards universal coverage for reproductive, maternal, newborn, and child health. *The Lancet*. 2018;391:1538-1548.
10. Nabwera HM, Wang D, Tongo OO, et al. Burden of disease and risk factors for mortality amongst hospitalized newborns in Nigeria and Kenya. *PloS one*. 2021;16: e0244109.
11. Bhutta ZA, Giuliani F, Haroon A, et al. Standardisation of neonatal clinical practice. *BJOG*. 2013;120:56-63.
12. Federal Ministry of Health. Nigeria Every Newborn Action Plan: A plan to end preventable newborn deaths in Nigeria. 2016.
13. Ministry of Health. Health ministry launches policy to accelerate gains in child health. 2018 [Available from: http://www.health.go.ke/health-ministry-launches-policytoaccelerate-gains-in-child-health-nairobi-kenya-november-19-2018/.
14. Ballot DE, Chirwa TF, Cooper PA. Determinants of survival in very low birth weight neonates in a public sector hospital in Johannesburg. *BMC Pediatr.* 2010;10:30.

For peer review only - http://bmjopen.bmj.com/site/about/guidelines.xhtml

15. Engel N, Wachter K, Pai M, et al. Addressing the challenges of diagnostics demand and supply: insights from an online global health discussion platform. *BMJ Glob Health*. 2016;1:e000132.
16. Welcome MO. The Nigerian health care system: Need for integrating adequate medical intelligence and surveillance systems. *J Pharm Bioallied Sci*. 2011;3:478.
17. English M, Gathara D, Nzinga J, et al. Lessons from a Health Policy and Systems Research programme exploring the quality and coverage of newborn care in Kenya. *BMJ Glob Health*. 2020;5:e001937.
18. Liverpool School of Tropical Medicine. About the Neonatal Nutrition Network [Available from: https://www.lstmed.ac.uk/research/departments/clinicalsciences/neonatal-nutritionnetwork/about-the-neonatal-nutrition.
19. Harris PA, Taylor R, Minor BL, et al. The REDCap consortium: Building an international community of software platform partners. *J Biomed Inform.* 2019;95:103208.
20. IMB Corp. IBM SPSS statistics for Windows, version 20.0. 2011.
21. World Health Organization. Pocket book of hospital care for children: guidelines for the management of common childhood illnesses. 2013.
22. Akenroye AT, Baskin MN, Samnaliev M, et al. Impact of a bronchiolitis guideline on ED resource use and cost: a segmented time-series analysis. *Pediatrics*. 2014;133:227-234.
23. Lopez AA, Aslanova R, Bridger N, et al. Antibiotic Use for Inpatient Bronchiolitis: Did National Guidelines Impact Practice at a Pediatric Hospital? *Hospital Pediatrics*. 2020;10:147-152.
24. Irimu G, Ogero M, Mbevi G, et al. Tackling health professionals' strikes: an essential part of health system strengthening in Kenya. *BMJ Glob Health*. 2018;3:e001136.
25. UNICEF. Country Office Annual Report- Kenya. unicef.org; 2018.
26. Majors CE, Smith CA, Natoli ME. Point-of-care diagnostics to improve maternal and neonatal health in low-resource settings. *Lab on a Chip*. 2017;17:3351-3387.
27. Mabey D, Vos T. Syndromic approaches to disease management. *The Lancet*. 1997;349:26-28.
28. Ellington LE, Gilman RH, Chavez MA, et al. Lung ultrasound as a diagnostic tool for radiographically-confirmed pneumonia in low resource settings. *Respiratory Medicine*. 2017;128:57-64.

For peer review only - http://bmjopen.bmj.com/site/about/guidelines.xhtml

29. Camacho-Gonzalez A, Spearman PW, Stoll BJ. Neonatal infectious diseases: evaluation of neonatal sepsis. *Pediatric Clinics*. 2013;60:367-389.

30. Gallacher DJ, Hart K, Kotecha S. Common respiratory conditions of the newborn. *Eur Respir* J. 2016;12:30-42.

31. Lemyre B, Chau V. Hypothermia for newborns with hypoxic-ischemic encephalopathy. *Paediatrics & child health*. 2018;4:285-295.

32. Filkins M, Halliday S, Daniels B, et al. Implementing Diagnostic Imaging Services in a Rural Setting of Extreme Poverty: Five Years of X-ray and Ultrasound Service Delivery in Achham, Nepal. *Journal of Global Radiology*. 2015;1:1-2.

33. Gephart SM, Gordon PV, Penn AH, et al. Changing the paradigm of defining, detecting, and diagnosing NEC: Perspectives on Bell's stages and biomarkers for NEC. *Semin Pediatr Surg*. 2018;27:3-10.

34. Schito M, Peter TF, Cavanaugh S, et al. Opportunities and Challenges for CostEfficient Implementation of New Point-of-Care Diagnostics for HIV and Tuberculosis.
J Infect Dis. 2012;205:169-180.

35. Drain PK, Hyle EP, Noubary F, et al. Diagnostic point-of-care tests in resourcelimited settings. *Lancet Infect Dis*. 2014;14:239-249.

For peer review only

For peer review only - http://bmjopen.bmj.com/site/about/guidelines.xhtml

Figure 1: Heatmaps showing the diagnostic criteria used for common neonatal conditions

Figure 1a: Suspected neonatal sepsis

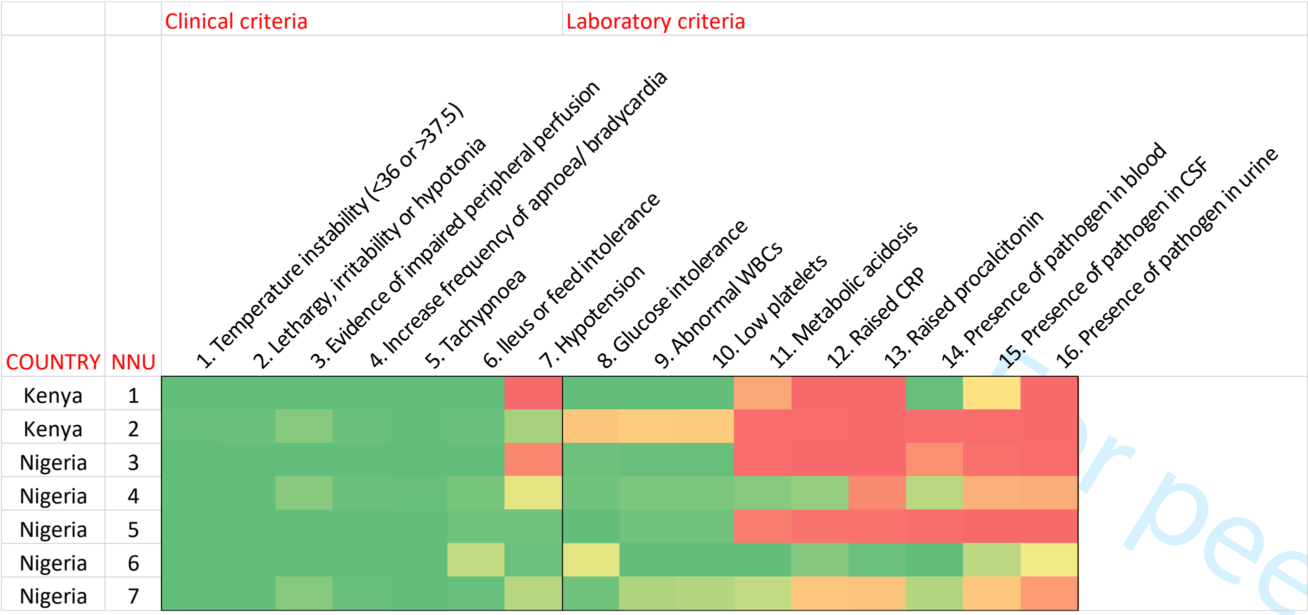

Figure 1c: Birth asphyxia

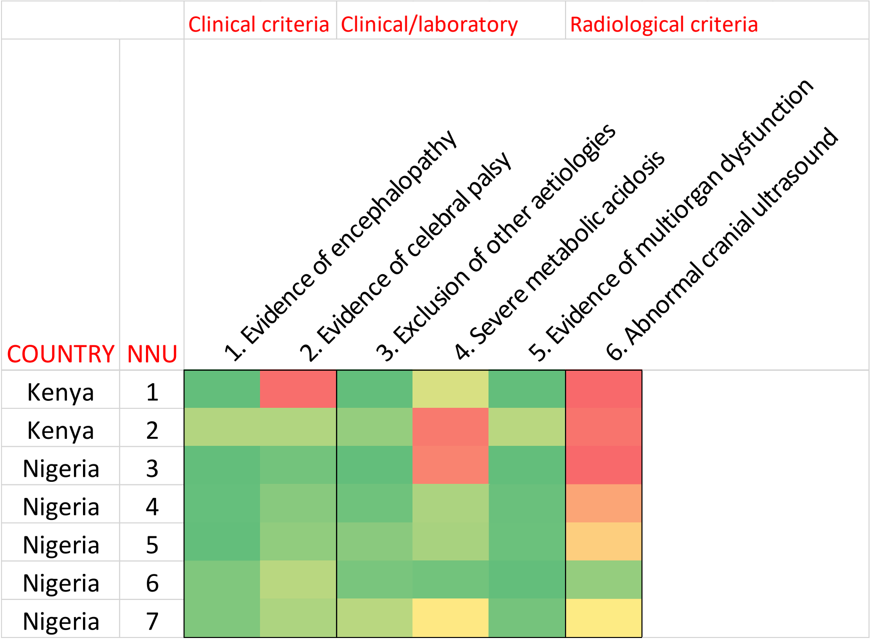

Figure 1b: Respiratory conditions

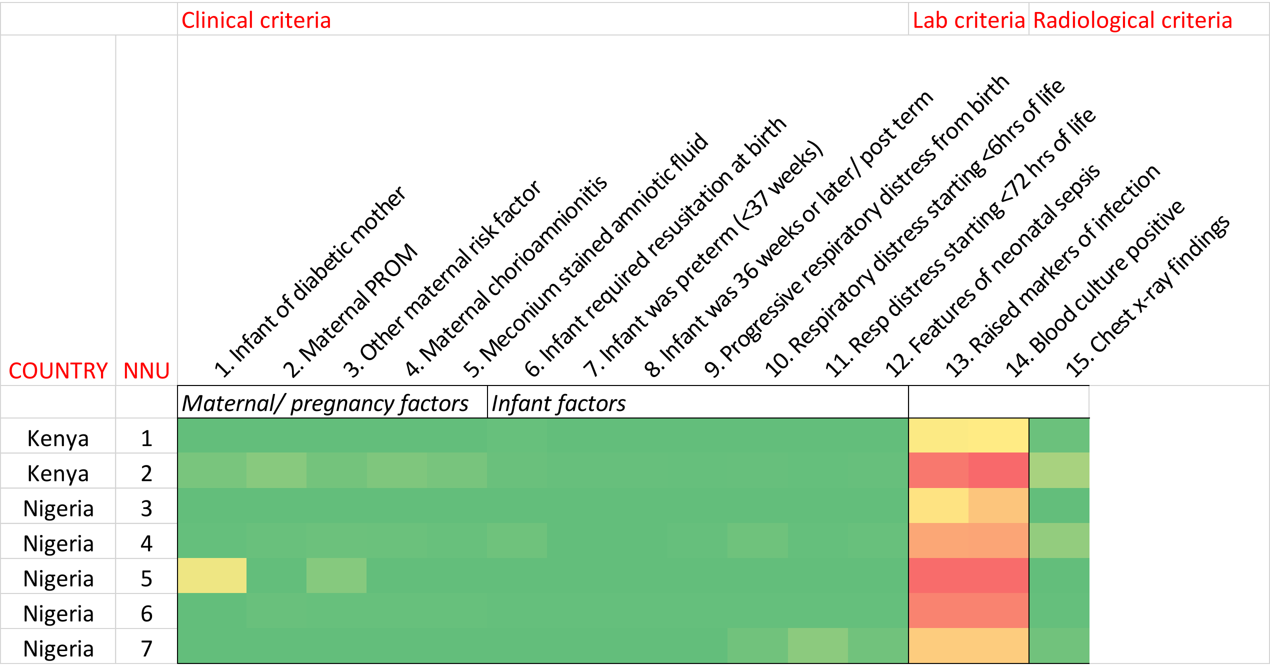

Figure 1d: Abdominal conditions

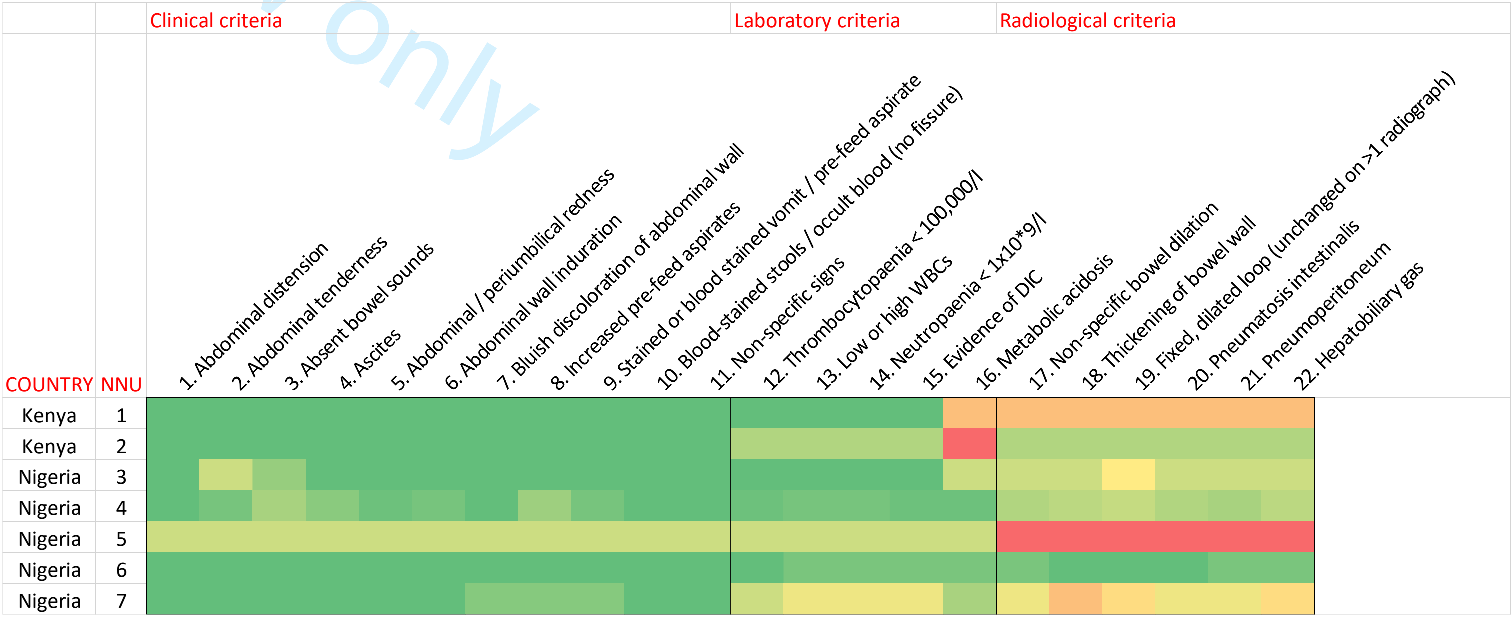

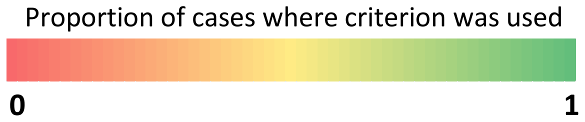

For peer review only

***Appendix 1**: Neonatal Nutrition Network Members*

Isa Abdulkadir (Ahmadu Bello University, Nigeria); Ismaela Abubakar (LSTM, UK); Abimbola E Akindolire (College of Medicine, University of Ibadan, Nigeria); Olusegun Akinyinka (College of Medicine, University of Ibadan, Nigeria); Stephen J Allen (LSTM, UK); Pauline EA Andang'o (Maseno University, Kenya); Graham Devereux (LSTM, UK); Chinyere Ezeaka (Lagos University Teaching Hospital, Nigeria); Beatrice N Ezenwa (Lagos University Teaching Hospital, Nigeria); Iretiola B Fajolu (Lagos University Teaching Hospital, Nigeria); Zainab O Imam (Lagos State University Teaching Hospital, Nigeria); Kevin Mortimer (LSTM, UK); Martha K Mwangome (KEMRI Wellcome Trust Research Programme, Kenya); Helen M Nabwera (LSTM, UK); Grace M Nalwa (Jaramogi Oginga Odinga Teaching and Referral Hospital, Kenya & Maseno University, Kenya); Walter Otieno (Jaramogi Oginga Odinga Teaching and Referral Hospital, Kenya & Maseno University, Kenya); Alison W Talbert (KEMRI Wellcome Trust Research Programme, Kenya); Nicholas D Embleton (Newcastle University, UK); Olukemi O Tongo (College of Medicine, University of Ibadan, Nigeria); Dominic D Umoru (Maitama District Hospital, Nigeria); Janneke van de Wijgert (University of Liverpool, UK); Melissa Gladstone (University of Liverpool, UK).

For peer review only

Appendix 2: Neonatal unit details

Location	Rural or urban	Secondary of tertiary level	No. of neonatal beds	Staffing
The Neonatal Unit, Lagos University Teaching Hospital (luth), Lagos, Nigeria	Urban	Tertiary	80 beds	- 4 consultant neonatologists<br>- 10 resident doctors<br>- 26 nurses<br>- 8 support staff
The Neonatal Unit of Ahmadu, Bello University Teaching Hospital Shika, Zaria, Kaduna, Nigeria	Urban	Tertiary	24 beds	- 3 neonatologists<br>- 5 senior registrars<br>- 4 registrars<br>- 18 nurses<br>- Support staff
Neonatal Unit, Maitama District Hospital, Abuja, Nigeria	Urban	Secondary	8 beds	- 3 neonatologists<br>- 2 medical Officers<br>- 2-3 nurses<br>- Support staff
Massey Street Children's Hospital, Lagos Island, Nigeria	Urban	Tertiary	56 beds	- 2 neonatologists<br>- 1 senior registrar<br>- 4 registrars<br>- 31 nursing staff
University College Hospital, Ibadan, Nigeria	Urban	Tertiary	38 beds	- 3 neonatologists<br>- 4 senior registrars<br>- 4 registrars<br>- House officers<br>- 50 nurses
Kilifi County Hospital, Kilifi Town, Kenya	Urban	Secondary	18 beds	- 2 paediatric consultants,<br>- 6 clinical officers<br>- 3-4 neonatal nurses
Jaramogi Oginga Odinga Teaching and Referral Hospital (JOOTRH), Kisumu City, Kenya	Urban	Tertiary	21 beds	- 2 paediatric consultants,<br>- 2 medical officers<br>- 1-2 medical officers<br>- 1-2 neonatal nurses

For peer review only

Appendix 3a: Ethical approval details from collaborating institutions

Institution	Protocol number
**Liverpool School of Tropical Medicine Research Ethics Committee (REC)**	18-0210
**The Lagos University Teaching Hospital Health Research Ethics Committee**	AMD/DCST/HREC/APP/2514
**The research and ethics committees at The Jaramogi Oginga Odinga Teaching and Referral Hospital**	ERC.IB/VOL.1/510
**University College Hospital Ibadan**	UI/EC/18/0446
**Massey Street Children's Hospital**	LSHSC/2222/VOL.VI^{B}/185
**Ahmadu Bello University Teaching Hospital**	ABUTH/HZ/HREC/D37/2018
**Maitama District Hospital**	FHREC/2018/01/108/19-09-18
**The Kenya Medical Research Institute-Scientific and Ethics Review Unit**	KEMRI/SERU/CGMR-C/120/3740

For peer review only

Appendix 3b: Confirmation of data access request from KEMRI- Wellcome Trust

1. REQUESTOR DETAILS

**Name**	Aimee Staunton				
**Contact details: address, phone and email**	97 Aigburth Road Liverpool L17 4JU Liverpool School of Tropical Medicine	**Phone**	07530351521	**Email**	264266@lstmed.ac.uk

2. STUDY DETAILS

**Title of Research Project**	Describing the diagnostic pathways for common neonatal conditions across seven neonatal units in sub-Saharan Africa	**Start & end dates**	June 2020- September 2020
**Ethical approval sought / planned (with dates)**	Ethics application covered by existing approved proposal:<br><br>Title of proposal: Improving the survival, growth and development of low birth weight newborns through better nutrition<br>LSTM Research Ethics Committee, reference number: 18-0210<br>PI: Prof Stephen Allen<br>Date of approval: 4th July 2018<br>End date for data collection: 31st August 2019		

3. INVESTIGATORS

Names of all investigators who will have access to data (begin with Principal Investigator)* include names of KWTRP collaborators where applicable.

**Name**	**Role**	**Institution**
Aimee Staunton	Principal Investigator (MSc student)	LSTM
Helen Nabwera	Project Supervisor	LSTM
Stephen Allen	Project Supervisor	LSTM

4. BRIEF DESCRIPTION TYPE OF DATA REQUIRED

The dataset collected by NeoNuNet to record diagnostic pathways, comprising all admissions to the seven network neonatal units over a six-month period between August 2018 and May 2019. This includes 2280 neonates in total.

Title: Improving the survival, growth and development of low birth weight newborns through better nutrition

PI: Dr Martha Mwangome

KEMRI/SERU/CGMT-C/120/3740

5. OBJECTIVES AND STUDY DETAILS INCLUDING TIME FRAME

1. To describe the population of neonates and the causes of admission to secondary and tertiary level neonatal units in sub-Saharan Africa using secondary data collected from seven neonatal units in Nigeria and Kenya by September 2020.
2. To describe how common neonatal conditions (asphyxia, respiratory disorders, abdominal conditions/ necrotizing enterocolitis and neonatal sepsis) are diagnosed in the seven neonatal units in Nigeria and Kenya by September 2020.
3. To identify any gaps or inaccuracy in the diagnostic pathways for common neonatal conditions in the seven neonatal units and explore the association with neonatal outcomes by September 2020.

6. BRIEF DESCRIPTION OF ANALYSIS PLANNED WITHIN KIDMS DATA ***specify if study specific data not KIDMS data**

Objective 1: To fulfil this objective, data will be analysed using SPSS to describe the population of neonates. The relative percentages of the different diagnoses will be calculated. Descriptive statistics for variables such as gestation, sex and birthweight will be generated and basic hypothesis testing will be carried out. This will help to describe the biodemographic characteristics of the population being studied.

Objective 2: To fulfil this objective, the diagnostic pathways that lead to the final diagnosis (birth asphyxia, respiratory disorders, abdominal conditions/ necrotizing enterocolitis (NEC) or neonatal sepsis) will be analysed. This will be

Conditions and limitations for data sharing

You understand and acknowledge that custodianship of the Data is held jointly by the Programme and the Ministry of Health in Kilifi, and will be retained by the Programme and the Ministry of Health in Kilifi in case of any data transfer to a Recipient.
You understand and acknowledge that the Data is experimental in nature, and that access to the Data is provided without any representations or warranties of any kind in relation to the Database or the Data.
You will only access the Database and use the Data for the Agreed Purpose and shall not use the Data in such a way that damage or distress is or is reasonably likely to be caused to any Data Subjects.
The Data relates directly to individual Data Subjects and is strictly confidential. Data from subjects will be shared in a fully anonymised form without access to personal identifiers, including names and/or specialised roles. Geographic identifiers for subjects will routinely be aggregated to the level of the sub location or similarly de-identified. Consideration of requests for data with greater granularity will be informed by the local importance of the research question and the ability to protect the confidentiality of individuals and communities.
You shall only disclose the Data to your Data Users. You shall take all reasonable measures to ensure that your Data Users shall not make copies of the whole or any part of the Data without your consent, and shall keep a written record of any such copies sufficient to permit you to fulfil your obligations under clauses 3 and 7 of this Agreement. You shall not transfer or disclose any part of the Data to any other person or body.
You understand and acknowledge that the Data is protected by copyright and other intellectual property rights.
You agree at all times to keep strictly confidential, and ensure that the Data Users keep confidential the information and Data pertaining to Data Subjects. In particular, You undertake not to use, or attempt to use, or permit anyone other than the Data Users to use the Data on its own or in conjunction with other data, to seek to discover the identity of any Data Subjects, to compromise or otherwise infringe the confidentiality of information on Data Subjects and their right to privacy.
You accept that the Data is protected by and subject to national and international laws, and that You are responsible for ensuring compliance with any applicable laws.
You agree that you will submit a summary report and any other information reasonably requested to the KEMRI Wellcome Trust Research Programme for the purposes of monitoring data use on the understanding that such information, data, results, and conclusions contained within will be treated as confidential information belonging to the Recipient.
This agreement is not transferable and You may not purport to assign it (in whole or in part) without the written consent of the KEMRI Wellcome Trust Research Programme.
If You commit a material breach of this Agreement or for any persistent breach of this Agreement, the KEMRI Wellcome Trust may terminate this Agreement immediately by notice in writing, without prejudice to its accrued rights and remedies.
You accept that it may be necessary for the KEMRI Wellcome Trust Research Programme to alter the terms of this Agreement from time to time in order to address new concerns. In this event, the KEMRI Wellcome Trust Research Programme will contact You to inform You of any changes and You agree that Your continued use of the Data shall be dependent on the parties entering into a variation of the Agreement.
If requested to do so by the Data Governance Committee, the KEMRI Wellcome Trust Research Programme may terminate this Agreement at any time by giving one month's notice in writing to You.
Your duty to protect the confidentiality of the Data received under this agreement shall survive termination of this Agreement and shall continue in full force and effect indefinitely.
Upon termination of this Agreement you will permanently delete all copies of the Data from your computer systems and storage devices and will ensure that all Your Data Users shall also permanently delete such copies. You may retain aggregate information on the proviso that such aggregate information does not allow any individuals or groups of individuals who are the subject of the Data to be identified with reasonable effort.
You may publish Your results arising from the use of the Data for the Agreed Purpose providing the Data itself is not disclosed. Aggregate or generic information generated from the Data may be published on the provisos that: i) such aggregate or generic information does not allow Data Subjects or groups of Data Subjects to be identified with reasonable effort; ii) no damage or distress is or is reasonably likely to be caused to any Data Subjects or groups of Data Subjects; iii) the Data will not be used in any way that could reasonably be expected to lead to ethnic stigmatisation; and iv) no attempt will be made to identify the Data Subjects.
You agree to acknowledge the KEMRI Wellcome Trust Research Programme or appropriate Programme researchers (via authorship or an acknowledgment statement) in any work based in whole or part on the Data in the following way :*"This paper/publication has used data and/or biological samples provided by the KEMRI Wellcome Trust Research programme in accordance with the consent provided by participants and approved by the KEMRI Ethics Review Committee".*
You agree that if your application is approved, information about the proposed research use can be posted on the KEMRI Wellcome Trust Research Programme's public website. The information may include Your name and institution, the title of the project, and a non-technical summary of the research question.
You recognise that nothing in this Agreement shall operate to transfer to You any intellectual property rights relating to the Data. You have the right to develop intellectual property based on comparisons with Your own data.

Definitions in the agreement above:
"Recipient" means the principal researcher named above; "Recipient Institution" means the organisation named above at which the Recipient is employed, affiliated or enrolled; "Data Users" means those officers, employees and students of the Recipient Institution, who work directly with the Recipient and have a need to use the Data for the performance of their work with respect to the Agreed Purpose, and have agreed to comply with this Agreement, as named in the application; "Database" the KIDMS database containing clinical and demographic and health surveillance data on Data Subjects; "Data Subjects" the individuals who have contributed their data to the Database; "Data" the data within the Database; "Agreed Purpose" means the medical research purpose(s) approved by the Data Governance Committee in writing; "Publications" means, without limitation, articles published in print journals, electronic journals, reviews, books, posters and other written and verbal presentations of research.

Statement of agreement: I agree to the above conditions and limitations for sharing data as requested in this application:

Name:____AIMEE STAUNTON____________________
Date:____04/06/20____________________
Signature:____________________

Signed on behalf of KEMRI-Wellcome Trust Research Programme:

....

Title:...Professor Philip Bejon..

Position:...Programme Executive Director..

Signature: ....

Date:........29/Jun/2020..

For peer review only

Appendix 4: Data from heatmaps in Figures 1-4 showing diagnostic criteria use in common neonatal conditions.

4a. Suspected sepsis

NEONATAL SEPSIS																
	CRITERIA NUMBER															
NNU	1.	2.	3.	4.	5.	6.	7.	8.	9.	10.	11.	12.	13	14.	15.	16.
1	1.00	1.00	1.00	1.00	1.00	1.00	0.00	1.00	1.00	1.00	0.24	0.00	0.00	0.99	0.46	0.01
2	0.99	0.98	0.89	0.98	1.00	0.98	0.79	0.35	0.38	0.38	0.02	0.01	0.01	0.02	0.01	0.01
3	1.00	1.00	1.00	1.00	1.00	1.00	0.12	0.97	0.99	0.99	0.01	0.00	0.00	0.15	0.03	0.01
4	1.00	1.00	0.88	0.98	0.99	0.93	0.59	0.96	0.92	0.92	0.89	0.84	0.13	0.72	0.27	0.26
5	1.00	1.00	1.00	1.00	1.00	0.96	0.96	1.00	0.96	0.96	0.08	0.04	0.04	0.02	0.00	0.00
6	1.00	1.00	1.00	1.00	1.00	0.70	0.98	0.59	1.00	1.00	1.00	0.89	0.98	1.00	0.72	0.54
7	1.00	1.00	0.89	0.99	1.00	0.99	0.73	0.96	0.76	0.74	0.70	0.36	0.35	0.75	0.37	0.19

For peer review only

4b. Respiratory conditions

**RESPIRATORY CONDITIONS**															
	CRITERIA NUMBER														
NNU	1	2.	3.	4.	5.	6.	7.	8.	9.	10.	11.	12.	13.	14.	15.
1	1.00	1.00	1.00	1.00	1.00	0.99	1.00	1.00	1.00	1.00	1.00	1.00	0.51	0.50	0.97
2	0.93	0.88	0.95	0.91	0.93	0.98	0.99	0.99	0.99	0.99	0.99	0.99	0.10	0.04	0.78
3	1.00	1.00	1.00	1.00	1.00	1.00	1.00	1.00	1.00	1.00	1.00	1.00	0.47	0.37	1.00
4	1.00	0.98	0.97	0.98	0.99	0.96	1.00	1.00	0.99	0.96	1.00	0.99	0.26	0.26	0.85
5	0.56	1.00	0.89	1.00	1.00	1.00	1.00	1.00	1.00	1.00	1.00	1.00	0.06	0.06	1.00
6	1.00	0.98	0.99	0.99	0.99	0.99	0.99	0.99	0.99	0.99	0.99	0.99	0.14	0.14	0.99
7	1.00	1.00	1.00	1.00	1.00	1.00	1.00	1.00	1.00	0.96	0.87	0.96	0.39	0.39	0.96

For peer review only

*4*c. Birth asphyxia

BIRTH ASPHYXIA						
	CRITERIA NUMBER					
NNU	1.	2.	3.	4.	5.	6.
1	1.00	0.02	1.00	0.63	1.00	0.00
2	0.75	0.75	0.84	0.07	0.72	0.04
3	1.00	0.95	1.00	0.10	1.00	0.00
4	0.99	0.88	0.96	0.77	0.98	0.23
5	1.00	0.85	0.88	0.78	0.98	0.39
6	0.91	0.73	0.93	0.95	1.00	0.84
7	0.91	0.76	0.73	0.49	0.95	0.51

For peer review only

4d. Abdominal conditions

**ABDOMINAL CONDITIONS**																						
	CRITERIA NUMBER																					
NNU	1.	2.	3.	4.	5.	6.	7.	8.	9.	10.	11.	12.	13.	14.	15.	16.	17.	18.	19.	20.	21.	22
1	1.00	1.00	1.00	1.00	1.00	1.00	1.00	1.00	1.00	1.00	1.00	1.00	1.00	1.00	1.00	0.33	0.33	0.33	0.33	0.33	0.33	0.33
2	1.00	1.00	1.00	1.00	1.00	1.00	1.00	1.00	1.00	1.00	1.00	0.75	0.75	0.75	0.75	0.00	0.75	0.75	0.75	0.75	0.75	0.75
3	1.00	0.67	0.83	1.00	1.00	1.00	1.00	1.00	1.00	1.00	1.00	1.00	1.00	1.00	1.00	0.67	0.67	0.67	0.50	0.67	0.67	0.67
4	1.00	0.94	0.78	0.88	0.97	0.94	1.00	0.81	0.94	1.00	1.00	0.97	0.94	0.94	0.97	0.97	0.75	0.72	0.69	0.75	0.78	0.72
5	0.67	0.67	0.67	0.67	0.67	0.67	0.67	0.67	0.67	0.67	0.67	0.67	0.67	0.67	0.67	0.67	0.00	0.00	0.00	0.00	0.00	0.00
6	1.00	1.00	1.00	1.00	1.00	1.00	1.00	1.00	1.00	1.00	1.00	1.00	0.93	0.93	0.93	0.93	0.93	1.00	1.00	1.00	0.93	0.93
7	1.00	1.00	1.00	1.00	1.00	1.00	0.89	0.89	0.89	1.00	1.00	0.67	0.56	0.56	0.56	0.78	0.56	0.33	0.44	0.56	0.56	0.44

1. For Appendix 4 data: 1.00 means in 100% of cases the diagnostic criterion was used by clinicians

For peer review only

The RECORD statement – checklist of items, extended from the STROBE statement, that should be reported in observational studies using routinely collected health data.

	Item No.	STROBE items	Location in manuscript where items are reported	RECORD items	Location in manuscript where items are reported
**Title and abstract**					
	1	(a) Indicate the study's design with a commonly used term in the title or the abstract (b) Provide in the abstract an informative and balanced summary of what was done and what was found	Page 1-2	RECORD 1.1: The type of data used should be specified in the title or abstract. When possible, the name of the databases used should be included.<br><br>RECORD 1.2: If applicable, the geographic region and timeframe within which the study took place should be reported in the title or abstract.<br><br>RECORD 1.3: If linkage between databases was conducted for the study, this should be clearly stated in the title or abstract.	Page 2
**Introduction**					
Background rationale	2	Explain the scientific background and rationale for the investigation being reported	Page 4-5		
Objectives	3	State specific objectives, including any prespecified hypotheses	Page 4-5		
**Methods**					
Study Design	4	Present key elements of study design early in the paper	Page 5-6		
Setting	5	Describe the setting, locations, and relevant dates, including periods of recruitment, exposure, follow-up, and data collection	Page 5-6		

For peer review only

Participants	6	*(a) Cohort study* - Give the eligibility criteria, and the sources and methods of selection of participants. Describe methods of follow-up<br>*Case-control study* - Give the eligibility criteria, and the sources and methods of case ascertainment and control selection. Give the rationale for the choice of cases and controls<br>*Cross-sectional study* - Give the eligibility criteria, and the sources and methods of selection of participants<br><br>*(b) Cohort study* - For matched studies, give matching criteria and number of exposed and unexposed<br>*Case-control study* - For matched studies, give matching criteria and the number of controls per case		RECORD 6.1: The methods of study population selection (such as codes or algorithms used to identify subjects) should be listed in detail. If this is not possible, an explanation should be provided.<br><br>RECORD 6.2: Any validation studies of the codes or algorithms used to select the population should be referenced. If validation was conducted for this study and not published elsewhere, detailed methods and results should be provided.<br><br>RECORD 6.3: If the study involved linkage of databases, consider use of a flow diagram or other graphical display to demonstrate the data linkage process, including the number of individuals with linked data at each stage.	Page 5
Variables	7	Clearly define all outcomes, exposures, predictors, potential confounders, and effect modifiers. Give diagnostic criteria, if applicable.	Page 6	RECORD 7.1: A complete list of codes and algorithms used to classify exposures, outcomes, confounders, and effect modifiers should be provided. If these cannot be reported, an explanation should be provided.	n/a
Data sources/ measurement	8	For each variable of interest, give sources of data and details of methods of assessment (measurement).<br>Describe comparability of assessment methods if there is more than one group	Page 5-6		

For peer review only

Bias	9	Describe any efforts to address potential sources of bias	Page 5		
Study size	10	Explain how the study size was arrived at	Page 5		
Quantitative variables	11	Explain how quantitative variables were handled in the analyses. If applicable, describe which groupings were chosen, and why	Page 6		
Statistical methods	12	(a) Describe all statistical methods, including those used to control for confounding<br>(b) Describe any methods used to examine subgroups and interactions<br>(c) Explain how missing data were addressed<br>(d) *Cohort study* - If applicable, explain how loss to follow-up was addressed<br>*Case-control study* - If applicable, explain how matching of cases and controls was addressed<br>*Cross-sectional study* - If applicable, describe analytical methods taking account of sampling strategy<br>(e) Describe any sensitivity analyses	Page 6		
Data access and cleaning methods		..		RECORD 12.1: Authors should describe the extent to which the investigators had access to the database population used to create the study population.	Page 5-6

For peer review only

				RECORD 12.2: Authors should provide information on the data cleaning methods used in the study.	
Linkage		..		RECORD 12.3: State whether the study included person-level, institutional-level, or other data linkage across two or more databases. The methods of linkage and methods of linkage quality evaluation should be provided.	Page 5
**Results**					
Participants	13	(a) Report the numbers of individuals at each stage of the study (*e.g.*, numbers potentially eligible, examined for eligibility, confirmed eligible, included in the study, completing follow-up, and analysed)<br>(b) Give reasons for non-participation at each stage.<br>(c) Consider use of a flow diagram	Page 6-7	RECORD 13.1: Describe in detail the selection of the persons included in the study (*i.e.*, study population selection) including filtering based on data quality, data availability and linkage. The selection of included persons can be described in the text and/or by means of the study flow diagram.	Page 5
Descriptive data	14	(a) Give characteristics of study participants (*e.g.*, demographic, clinical, social) and information on exposures and potential confounders<br>(b) Indicate the number of participants with missing data for each variable of interest<br>(c) *Cohort study* - summarise follow-up time (*e.g.*, average and total amount)	Page 6-7<br>Table 1-2		
Outcome data	15	*Cohort study* - Report numbers of outcome events or summary measures over time<br>*Case-control study* - Report numbers in each exposure	n/a		

For peer review only

		category, or summary measures of exposure *Cross-sectional study* - Report numbers of outcome events or summary measures			
Main results	16	(a) Give unadjusted estimates and, if applicable, confounder-adjusted estimates and their precision (e.g., 95% confidence interval). Make clear which confounders were adjusted for and why they were included (b) Report category boundaries when continuous variables were categorized (c) If relevant, consider translating estimates of relative risk into absolute risk for a meaningful time period	n/a		
Other analyses	17	Report other analyses done—e.g., analyses of subgroups and interactions, and sensitivity analyses	Page 6-8		
**Discussion**					
Key results	18	Summarise key results with reference to study objectives	Page 8-10		
Limitations	19	Discuss limitations of the study, taking into account sources of potential bias or imprecision. Discuss both direction and magnitude of any potential bias	Page 10	RECORD 19.1: Discuss the implications of using data that were not created or collected to answer the specific research question(s). Include discussion of misclassification bias, unmeasured confounding, missing data, and changing eligibility over time, as they pertain to the study being reported.	n/a
Interpretation	20	Give a cautious overall interpretation of results considering objectives,	Page 11		

For peer review only

		limitations, multiplicity of analyses, results from similar studies, and other relevant evidence			
Generalisability	21	Discuss the generalisability (external validity) of the study results	Page 10		
**Other Information**					
Funding	22	Give the source of funding and the role of the funders for the present study and, if applicable, for the original study on which the present article is based	Page 6		
Accessibility of protocol, raw data, and programming code		..		RECORD 22.1: Authors should provide information on how to access any supplemental information such as the study protocol, raw data, or programming code.	Appendices Page 12

*Reference: Benchimol EI, Smeeth L, Guttmann A, Harron K, Moher D, Petersen I, Sørensen HT, von Elm E, Langan SM, the RECORD Working Committee. The REporting of studies Conducted using Observational Routinely-collected health Data (RECORD) Statement. *PLoS Medicine* 2015; in press.

*Checklist is protected under Creative Commons Attribution (CC BY) license.

For peer review only
